# Supplementary material for: In vivo single‐cell transcriptomics reveal Klebsiella pneumoniae skews lung macrophages to promote infection
Source: EMBO Mol Med. 2022 Nov 7;14(12):e16888. doi: 10.15252/emmm.202216888 (PMC9727930; doi:10.15252/emmm.202216888)
Supplement: Supplementary file 1 — Appendix [file EMMM-14-e16888-s006.docx]

**Appendix: In vivo single cell transcriptomics reveal *K. pneumoniae* skews lung macrophages to promote infection**

Amy Dumigan, Oisin Cappa, Brenda Morris, Joana Sá Pessoa, Ricardo Calderon-Gonzalez, Grant Mills, Rebecca Lancaster, David Simpson, Adrien Kissenpfennig, Jose A. Bengoechea

**Table of contents.**

Appendix Figure S1. Flow cytometric analysis of myeloid subsets in lungs of *K. pneumoniae*-infected mice…………………………………………………………………………………………2

Appendix Figure S2. Changes over pseudotime of selected genes within modules 3, 4 and 6……...4

Appendix Figure S3. *K. pneumoniae* induces M(Kp) polarisation in immortalized BMDMs………5

Appendix Figure S4. *K. pneumoniae*-induced M(Kp) polarization is STAT6 dependent…………...7

Appendix Figure S5. *K. pneumoniae*-induced M (Kp) polarisation is dependent on TLR signalling and the TLR adaptors MyD88, TRAM and TRIF……………………………………………………9

Appendix Figure S6. *K. pneumoniae*-induced M(Kp) polarisation is dependent on IL10……..…..11

Appendix Figure S7. Effect of inhibition of host metabolism on *K. pneumoniae*-macrophage interface………………………………………………………………………………………..……13

**Appendix Figure S1**. **Flow cytometric analysis of myeloid subsets in lungs of *K. pneumoniae*-infected mice.**

**A** and **B.** Gating strategy utilised to identify CD11b+CD11c-SiglecF- monocytes (MN), CD11b+CD11c+SiglecF- interstitial macrophages (IMs) and CD11b-CD11c+SiglecF+ tissue resident alveolar macrophages (AMs). Kp52145 was tagged with mCherry to allow the identification of macrophages with associated bacteria and bystander cells. This gating strategy was utilised also for FACS sorting of these populations for scRNAseq.

**C.** Absolute numbers of IMs in C57BL/6 mice treated with control liposomes (PBS) or clodronate ones intranasally (n= 6 per group).

**D.** Absolute numbers of AMs in C57BL/6 mice treated with control liposomes (PBS) or clodronate ones intranasally (n= 6 per group).

**E.** Absolute numbers of neutrophils in C57BL/6 mice treated with control liposomes (PBS) or clodronate ones intranasally (n= 6 per group).

**F.** Absolute numbers of IMs in C57BL/6 mice treated with control liposomes (PBS) or clodronate ones intravenously (n= 6 per group).

**G.** Absolute numbers of AMs in C57BL/6 mice treated with control liposomes (PBS) or clodronate ones intravenously (n= 6 per group).

**H.** Absolute numbers of neutrophils in C57BL/6 mice treated with control liposomes (PBS) or clodronate ones intravenously (n= 6 per group).

Data information: In C-H each dot represents one animal. Values are presented as the mean ± SEM. ** P ≤ 0.01; * P ≤ 0.05; ns, P > 0.05 for the indicated comparisons determined using unpaired t test.

**Appendix Figure S2. Changes over pseudotime of selected genes within modules 3, 4 and 6.**

Top selected genes from Dataset EV2.

# Appendix Figure S3. *K. pneumoniae* induces M(Kp) polarisation in immortalized BMDMs.

# A. *arg1* mRNA levels were assessed by qPCR in wild-type iBMDMs non-infected (ni) or infected with Kp52145 for 1, 3 or 5 h.

# B. Immunoblot analysis of Arg1 and tubulin levels in lysates from non-infected (ni) and infected wild-type cells with Kp52145 for 60 or 120 min.

# C. *fizz1* mRNA levels were assessed by qPCR in wild-type iBMDMs non-infected (ni) or infected with Kp52145 for 1, 3 or 5 h.

# D. Immunoblot analysis of Fizz1 and tubulin levels in lysates from non-infected (ni) and infected wild-type cells with Kp52145 for 60 or 120 min.

# E. *pparg* mRNA levels were assessed by qPCR in wild-type iBMDMs non-infected (ni) or infected with Kp52145 for 1, 3 or 5 h.

# F. *nos2* mRNA levels were assessed by qPCR in wild-type iBMDMs non-infected (ni) or infected with Kp52145 for 1, 3 or 5 h.

# G. *il12* mRNA levels were assessed by qPCR in wild-type iBMDMs non-infected (ni) or infected with Kp52145 for 1, 3 or 5 h.

# H. *il6* mRNA levels were assessed by qPCR in wild-type iBMDMs non-infected (ni) or infected with Kp52145 for 1, 3 or 5 h.

# I. *tnfa* mRNA levels were assessed by qPCR in wild-type iBMDMs non-infected (ni) or infected with Kp52145 for 1, 3 or 5 h.

# J. *il10* mRNA levels were assessed by qPCR in wild-type iBMDMs non-infected (ni) or infected with Kp52145 for 1, 3 or 5 h.

# K. Immunoblot analysis of phospho-STAT3 (pSTAT3) and tubulin levels in lysates from non-infected (ni) and infected wild-type cells with Kp52145 for 60 or 120 min.

Data information: For all infections, after 1 h contact, medium replaced with medium containing gentamycin (100 µg/ml) to kill extracellular bacteria. Error bars are presented as the mean ± SEM of three independent experiments in duplicate. Images are representative of three independent experiments. Statistical analysis were carried out using one-way ANOVA with Bonferroni contrast for multiple comparisons test. ****P ≤ 0.0001; **P≤ 0.01; *P ≤ 0.05 for the indicated comparisons.

**Appendix Figure S4. *K. pneumoniae*-induced M(Kp) polarization is STAT6 dependent.**

**A.** *klf4* mRNA levels were assessed by qPCR in wild-type (WT) and *stat6^-/-^* iBMDMs non-infected (ni) or infected with Kp52145 for 1, 3 or 5 h.

**B.** *pparg* mRNA levels were assessed by qPCR in wild-type (WT) and *stat6^-/-^* iBMDMs non-infected (ni) or infected with Kp52145 for 1, 3 or 5 h.

**C.** *fzz1* mRNA levels were assessed by qPCR in wild-type (WT) and *stat6^-/-^* iBMDMs non-infected (ni) or infected with Kp52145 for 1, 3 or 5 h.

**D.** *nos2* mRNA levels were assessed by qPCR in wild-type (WT) and *stat6^-/-^* iBMDMs non-infected (ni) or infected with Kp52145 for 1, 3 or 5 h.

**E.** *tnfa* mRNA levels were assessed by qPCR in wild-type (WT) and *stat6^-/-^* iBMDMs non-infected (ni) or infected with Kp52145 for 1, 3 or 5 h.

**F.** *il12* mRNA levels were assessed by qPCR in wild-type (WT) and *stat6^-/-^* iBMDMs non-infected (ni) or infected with Kp52145 for 1, 3 or 5 h.

**G.** *il6* mRNA levels were assessed by qPCR in wild-type (WT) and *stat6^-/-^* iBMDMs non-infected (ni) or infected with Kp52145 for 1, 3 or 5 h.

**H.** *isg15* mRNA levels were assessed by qPCR in wild-type (WT) and *stat6^-/-^* iBMDMs non-infected (ni) or infected with Kp52145 for 1, 3 or 5 h.

**I.** Percentage of wild-type (WT) and *stat6^-/-^* iBMDMs with and without associated Kp52145 positive for Arg1 1, 3 or 5 h post infection. Kp52145 was tagged with mCherry.

**J.** Percentage of wild-type (WT) and *stat6^-/-^* iBMDMs with and without associated Kp52145 positive for CD206 1, 3 or 5 h post infection. Kp52145 was tagged with mCherry.

**K.** Percentage of wild-type (WT) and *stat6^-/-^* iBMDMs with and without associated Kp52145 positive for MHC-II 1, 3 or 5 h post infection. Kp52145 was tagged with mCherry.

Data information: For all infections, after 1 h contact, medium replaced with medium containing gentamycin (100 µg/ml) to kill extracellular bacteria. qPCR and flow cytometry values are presented as the mean ± SEM of three independent experiments measured in duplicate. ****P ≤ 0.0001; ***P ≤ 0.001; **P≤ 0.01; ns, P > 0.05 for the indicated comparisons using one way-ANOVA with Bonferroni contrast for multiple comparisons test.

**Appendix Figure S5. *K. pneumoniae*-induced M (Kp) polarisation is dependent on TLR signalling and the TLR adaptors MyD88, TRAM and TRIF.**

**A.** *nos2* mRNA levels were assessed by qPCR in wild-type (WT) and *tlr2^-/-^*, *tlr4^-/-^* and *tlr2/4^-/-^* iBMDMs non-infected (ni) or infected with Kp52145 for 1, 3 or 5 h.

**B.** *isg15* mRNA levels were assessed by qPCR in wild-type (WT) and *tlr2^-/-^*, *tlr4^-/-^* and *tlr2/4^-/-^* iBMDMs non-infected (ni) or infected with Kp52145 for 1, 3 or 5 h.

**C.** *mx1* mRNA levels were assessed by qPCR in wild-type (WT) and *tlr2^-/-^*, *tlr4^-/-^* and *tlr2/4^-/-^* iBMDMs non-infected (ni) or infected with Kp52145 for 1, 3 or 5 h.

**D.** *pparg* mRNA levels were assessed by qPCR in wild-type (WT) and *tlr2^-/-^*, *tlr4^-/-^* and *tlr2/4^-/-^* iBMDMs non-infected (ni) or infected with Kp52145 for 1, 3 or 5 h.

**E.** *pparg* mRNA levels were assessed by qPCR in wild-type (WT), *myd88^-/-^*, *tram/trif^-/-^* non-infected (ni) or infected with Kp52145 for 1, 3 or 5 h.

**F.** *isg15* mRNA levels were assessed by qPCR in wild-type (WT), *myd88^-/-^*, *tram/trif^-/-^* non-infected (ni) or infected with Kp52145 for 1, 3 or 5 h.

**G.** *mx1* mRNA levels were assessed by qPCR in wild-type (WT), *myd88^-/-^*, *tram/trif^-/-^* non-infected (ni) or infected with Kp52145 for 1, 3 or 5 h.

**H.** *nos2* mRNA levels were assessed by qPCR in wild-type (WT), *myd88^-/-^*, *tram/trif^-/-^* non-infected (ni) or infected with Kp52145 for 1, 3 or 5 h.

Data information: For all infections, after 1 h contact, medium replaced with medium containing gentamycin (100 µg/ml) to kill extracellular bacteria. Error bars are presented as the mean ± SEM of three independent experiments in duplicate. ****P ≤ 0.0001; ***P ≤ 0.001; **P≤ 0.01; *P ≤ 0.05; ns, P > 0.05 for the indicated comparisons using one way-ANOVA with Bonferroni contrast for multiple comparisons test.

**Appendix Figure S6. *K. pneumoniae*-induced M(Kp) polarisation is dependent on IL10.**

**A.** *nos2* mRNA levels were assessed by qPCR in wild-type (WT), *il10^-/-^* non-infected (ni) or infected with Kp52145 for 1, 3 or 5 h.

**B.** *tnfa* mRNA levels were assessed by qPCR in wild-type (WT), *il10^-/-^* non-infected (ni) or infected with Kp52145 for 1, 3 or 5 h.

**C.** *mx1* mRNA levels were assessed by qPCR in wild-type (WT), *il10^-/-^* non-infected (ni) or infected with Kp52145 for 1, 3 or 5 h.

**D.** *isg15* mRNA levels were assessed by qPCR in wild-type (WT), *il10^-/-^* non-infected (ni) or infected with Kp52145 for 1, 3 or 5 h.

**E.** Percentage of wild-type (WT) and *il10^-/-^* iBMDMs with and without associated Kp52145 positive for Arg1 1, 3 or 5 h post infection. Kp52145 was tagged with mCherry.

**F.** Percentage of wild-type (WT) and *il10^-/-^* iBMDMs with and without associated Kp52145 positive for CD206 1, 3 or 5 h post infection. Kp52145 was tagged with mCherry.

**G.** Percentage of wild-type (WT) and *il10^-/-^* iBMDMs with and without associated Kp52145 positive for MHCII 1, 3 or 5 h post infection. Kp52145 was tagged with mCherry.

Data information: For all infections, after 1 h contact, medium replaced with medium containing gentamycin (100 µg/ml) to kill extracellular bacteria. Error bars are presented as the mean ± SEM of three independent experiments in duplicate. Images are representative of three independent experiments. ****P ≤ 0.0001; **P≤ 0.01; ns, P > 0.05 for the indicated comparisons using one way-ANOVA with Bonferroni contrast for multiple comparisons test.

#

# Appendix Figure S7. Effect of inhibition of host metabolism on *K. pneumoniae*-macrophage interface.

**A.** Growth kinetics of Kp52145 cultures in LB containing the glycolysis inhibitor 2-doxyglucose (2DG, 3 μM), the FAO inhibitors oligomycin (1 μM) or etomoxir (50 μM), or DMSO vehicle control. Values are presented as the mean ± SEM of three independent experiments measured in triplicate

**B.** Adhesion of Kp52145 to iBMDMs treated with DMSO vehicle control or the glycolysis inhibitor 2-doxyglucose (2DG, 3 μM), the FAO inhibitors oligomycin (1 μM) or etomoxir (50 μM). Inhibitors were added 2 h before and maintained throught.

**C.** Phagocytosis of Kp52145 by iBMDMs treated with DMSO vehicle control or the glycolysis inhibitor 2-doxyglucose (2DG, 3 μM), the FAO inhibitors oligomycin (1 μM) or etomoxir (50 μM). Inhibitors were added 2 h before and maintained throughout.

**D.** Activation of NF-κB signalling measured by quantifying SEAP secreted to the supernatants of Raw-Blue cells (InvivoGen) following infection with Kp52145. Cells treated with DMSO, 2DG (3 μM), oligomycin (1 μM) or etomoxir (50 μM) 2 h before infection and maintained throughout experiment.

**E.** Activation of Irf3 measured by quantifying secreted Lucia luciferase to the supernatants of Raw-Lucia ISG cells (InvivoGen) following infection with Kp52145. Cells treated with DMSO, 2DG (3 μM), oligomycin (1 μM) or etomoxir (50 μM) 2 h before infection and maintained throughout experiment.

Data information: For all infections, after 1 h contact, medium replaced with medium containing gentamycin (100 µg/ml) to kill extracellular bacteria. Error bars are presented as the mean ± SEM of three independent experiments in duplicate. Statistical analysis were carried out using one-way ANOVA with Bonferroni contrast for multiple comparisons test. ****P ≤ 0.0001; ns, *P ≤ 0.05; n.s. P > 0.05 for the indicated comparisons.
