# Supplementary material for: In vivo single‐cell transcriptomics reveal Klebsiella pneumoniae skews lung macrophages to promote infection
Source: EMBO Mol Med. 2022 Nov 7;14(12):e16888. doi: 10.15252/emmm.202216888 (PMC9727930; doi:10.15252/emmm.202216888)
Supplement: Supplementary file 2 — Expanded View Figures PDF [file EMMM-14-e16888-s001.pdf]

Expanded View Figures

Figure EV1. Analysis of *Klebsiella pneumoniae*-induced transcriptome in IMs.

A Diagram of the experimental approach to generate the different IMs and AMs samples for single-cell RNA sequencing (scRNAseq). C57BL/6 mice ($n = 17$ per group) were infected intranasally with mCherry tagged Kp52145, after 24 h, lungs were excised, and processed for cell sorting. From pooled samples AM and IM populations were sorted from PBS controls and infected mice. In the latter group, cells were sorted to separate bystander cells and cells with associated bacteria. The viability of each of the samples was determined to be higher than 95% before carrying out 10× genomics single-cell RNA sequencing.

B Marker gene detection and differential expression testing was performed in Seurat using the MAST package. Higher resolution clustering using uniform manifold approximation and projection (UMAP) dimensionality reduction analysis showing selected genes, *cx3cr1*, IM marker and *siglecF*, AM marker.

C UMAP of clustering within cells from PBS mock-infected mice (control), bystander and Kp52145-associated IMs and AM populations.

D Network enrichment mapping generated from significantly upregulated genes of IMs with associated bacteria. Analysis was performed using the g:SCS method for multiple testing correction (gProflier), the Reactome database as a data source and the default settings for the other parameters in gProflier. Results were exported to Cytoscape and visualised using the AutoAnnotate plug.

E Network enrichment mapping generated from significantly upregulated genes of bystander IMs. Analysis was performed using the g:SCS method for multiple testing correction (gProflier), the Reactome database as a data source and the default settings for the other parameters in gProflier. Results were exported to Cytoscape and visualised using the AutoAnnoate application.

F Monocle analysis to determine the temporal pattern of gene expression over pseudotime in bystander and Kp52145-associated IMs from infected animals compared to PBS controls. Monocle analysis revealed 7 modules of genes showing similar pattern of expression.

G Heat map showing relative expression of the 7 modules found in IMs.

H Pathway analysis of modules 3 and 4 corresponding to Kp52145-infected IMs. Analysis was performed using the g:SCS method for multiple testing correction, the Reactome database as a data source and the default settings for the other parameters in G:profiler.

I STRING database was used to predict protein–protein interactions using the clustering algorithm MCL with default parameters using as data source the genes within modules 3 and 4.

J Pathway analysis of module 6 corresponding to bystander IMs. Analysis was performed using the g:SCS method for multiple testing correction, the Reactome database as a data source and the default settings for the other parameters in G:profiler.

©2022 The Authors

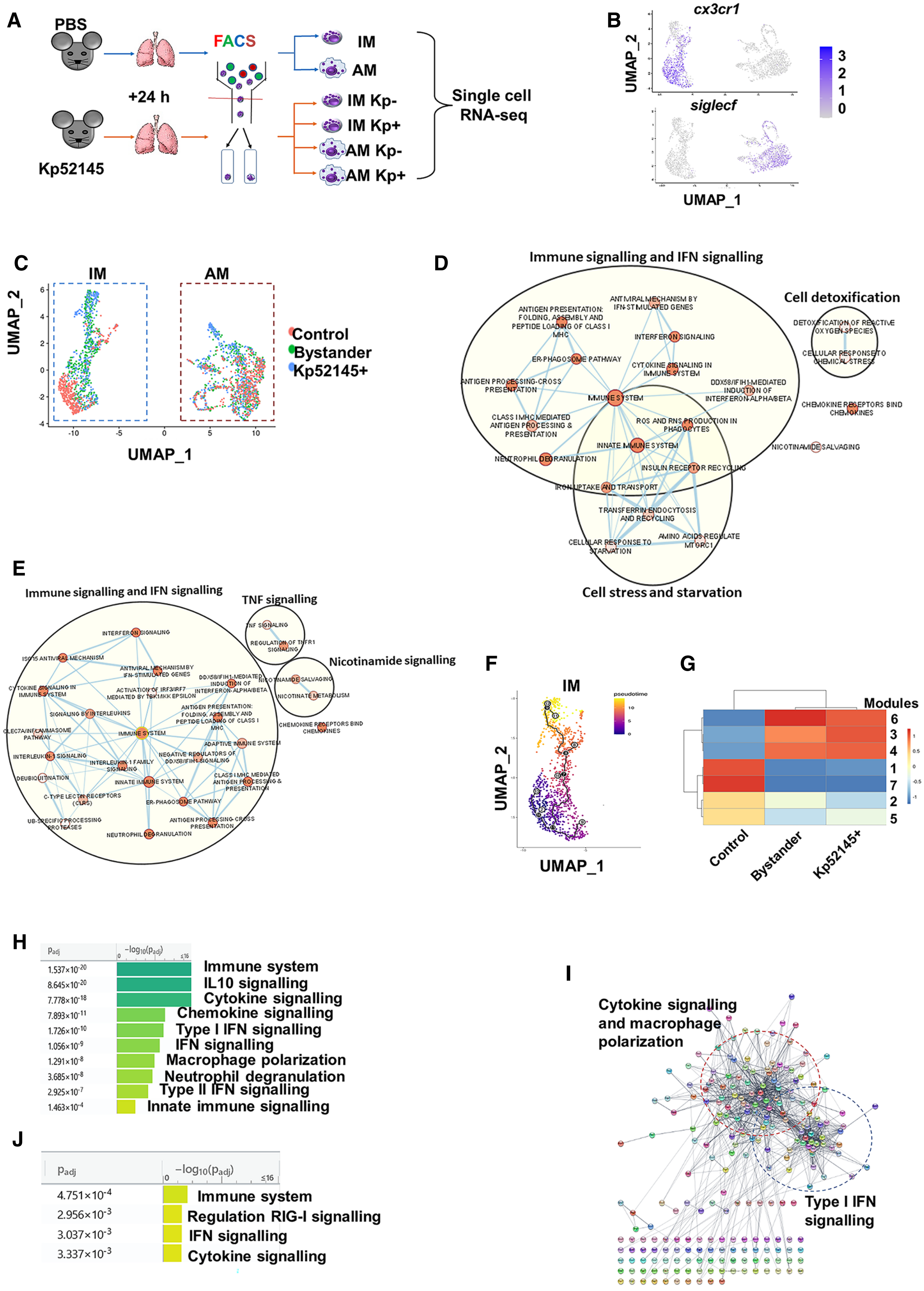

Figure EV1.

 ©2022 The Authors

Figure EV2. ***Klebsiella pneumoniae*** **skews macrophage polarisation towards a singular state termed M(Kp).**

A Heat map presents relative expression of the indicated genes between IMs from non-infected mice (control), and bystander and Kp52145-associated IMs from infected mice. Selected genes are related to M1 and M2 macrophage polarisation.

B Expression of M1 and M2 genes (shown in panel A) calculated as the average log-normalised expression. Each dot represents a cell, and the graph shows the mean and SEM per group of M1 and M2 genes. Statistical analysis were carried out using unpaired *t* test. The *P* values are indicated in the figure.

C–E Analysis by flow cytometry of the levels of M(Kp) markers expressed by cells from PBS mock infected mice (black dots), and by cells from infected mice (blue dots) with and without associated Kp52145. (C) Percentage of positive cells for Arg1. (D) Percentage of positive cells for Fizz1. (E) Percentage of positive cells for CD163.

Data information: Values in panel C-E are presented as the mean ± SEM whereby each dot represents an individual animal. ****$P \leq 0.0001$; **$P \leq 0.01$; ns, $P > 0.05$ for the indicated comparisons using one way-ANOVA with Bonferroni contrast for multiple comparisons test.

©2022 The Authors

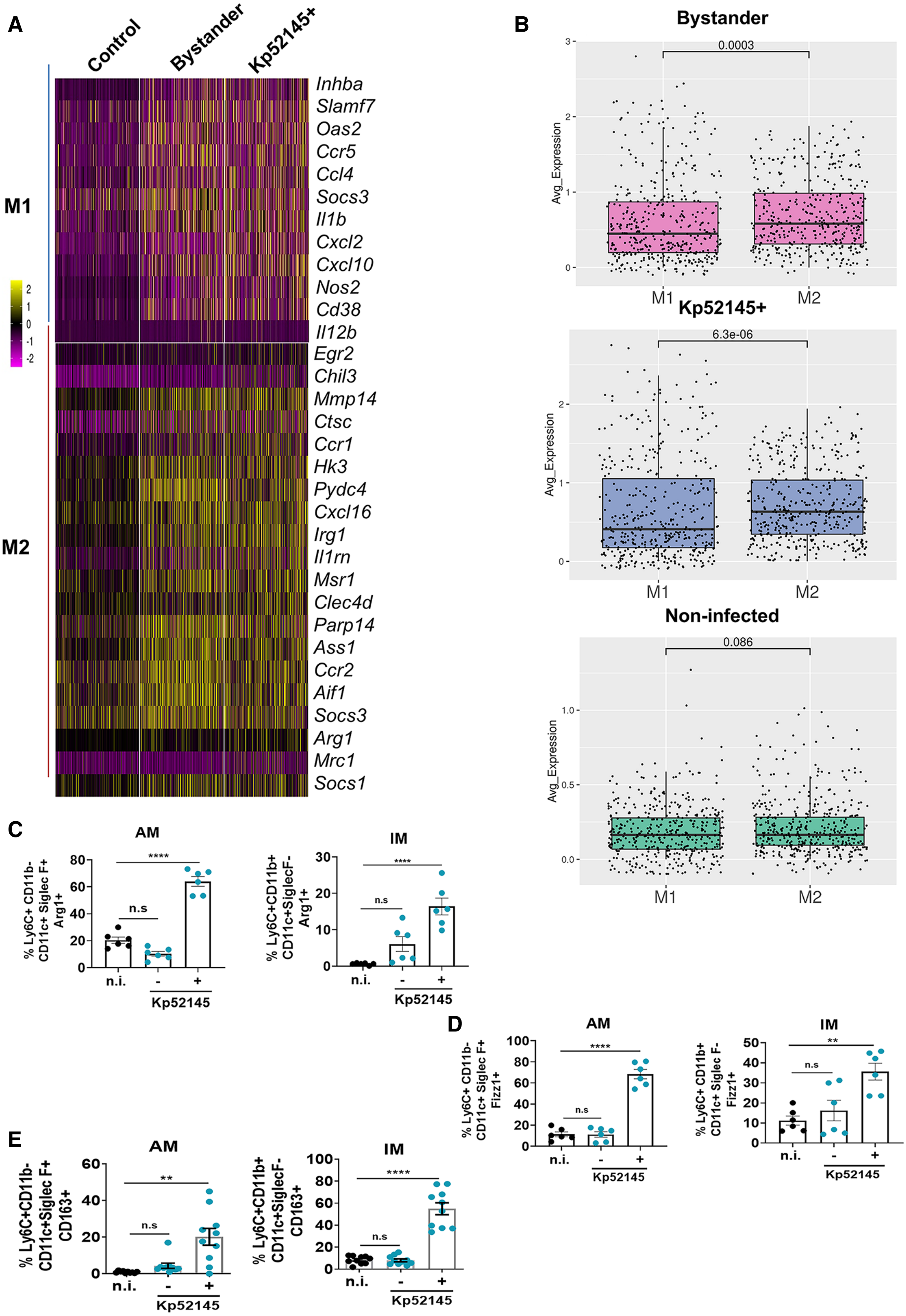

Figure EV2.

 ©2022 The Authors

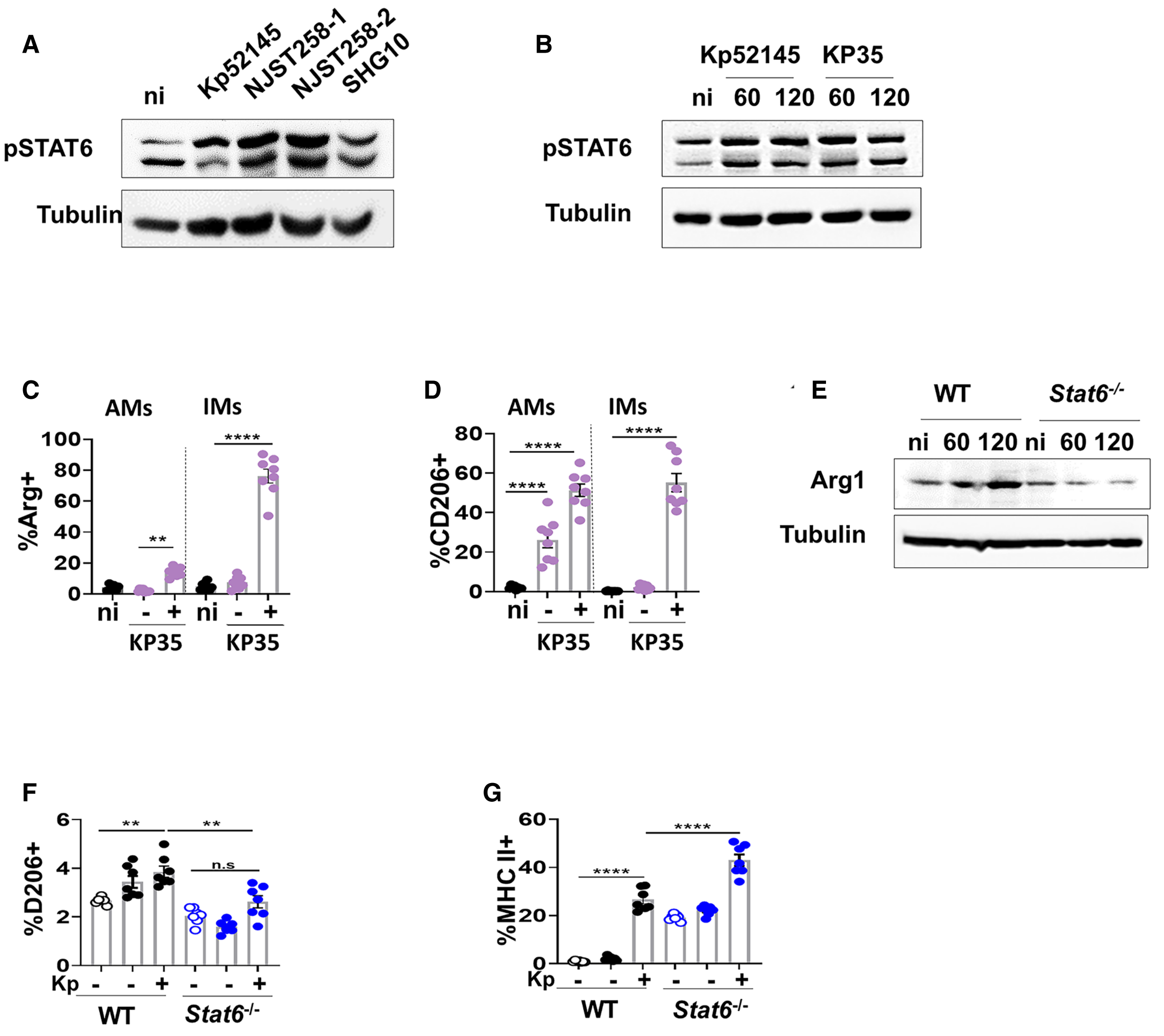

Figure EV3. ST258 *Klebsiella pneumoniae* strains induced M(Kp) polarisation.

A Immunoblot analysis of phospho-STAT6 (pSTAT6) and tubulin levels in lysates from non-infected (ni) and infected with different *K. pneumoniae* strains, Kp52145, NJST258-1, NJST258-2 or SHG10, for 60 min.

B Immunoblot analysis of phospho-STAT6 (pSTAT6) and tubulin levels in lysates from non-infected (ni) and infected with Kp52145 or KP35 for 60 or 120 min.

C, D Analysis by flow cytometry of the levels of M(Kp) markers expressed by IMs and AMs from PBS-mock-infected mice (black dots), and by IMs and AMs from infected mice (purple dots) with and without associated KP35 24 h post infection. (C) Percentage of positive cells for Arg1. (D) Percentage of positive cells for CD206.

E Immunoblot analysis of Arg1 and tubulin levels in lysates from wild-type and *stat6*$^{-/-}$ iBMDMs non-infected (ni) and infected with KP35 for 60 min or 120 min.

F Percentage of wild-type (WT) and *stat6*$^{-/-}$ iBMDMs with and without associated KP35 positive for CD206 5 h post infection. KP35 was tagged with mCherry.

G Percentage of wild-type (WT) and *stat6*$^{-/-}$ iBMDMs with and without associated KP35 positive for MCH-II 5 h post infection. KP35 was tagged with mCherry.

Data information: For all infections, after 1 h contact, medium replaced with medium containing gentamycin (100 μg/ml) to kill extracellular bacteria. Images are representative of three independent experiments. Error bars are presented as the mean ± SEM of three independent experiments in duplicate. Statistical analysis were carried out using one-way ANOVA with Bonferroni contrast for multiple comparisons test. ****$P \leq 0.0001$; **$P \leq 0.01$; ns, $P > 0.05$ for the indicated comparisons.

Figure EV4. Glycolysis characterises *Klebsiella pneumoniae*-induced M(Kp) polarisation.

A Dot Plot analysis of the expression levels of genes related to fatty acid oxidation (FAO) and glycolysis from the scRNAseq data set of PBS-infected IMs (control), and bystander and Kp52145-associated IMs. Dot size reflects percentage of cells in a cluster expressing each gene; dot colour intensity reflects expression level as indicated on legend.

B Extracellular acidification rate (ECAR, in mpH/min) of non-infected (ni) and Kp52145-infected iBMDMS (Kp52145) measured using Mito-stress test kit and the Seahorse XF analyser. When indicated oligomycin (2.5 μM), FCCP (2 μM), antimycin and roteanone (0.5 μM) were added to the cells.

C Oxygen consumption rates (OCR, in pMoles/min) of non-infected (ni) and Kp52145-infected iBMDMS (Kp52145) measured using Mito-stress test kit and the Seahorse XF analyser. When indicated oligomycin (2.5 μM), FCCP (2 μM), antimycin and roteanone (0.5 μM) were added to the cells.

D Basal respiration of non-infected (ni) and Kp52145-infected iBMDMs.

E Maximal respiration of non-infected (ni) and Kp52145-infected iBMDMs.

F Spare respiratory capacity of non-infected (ni) and Kp52145-infected iBMDMs.

G ATP production by non-infected (ni) and Kp52145-infected iBMDMs.

H Non mitochondrial O_2 consumption by non-infected (ni) and Kp52145-infected iBMDMs.

I Kp52145 intracellular survival in wild-type iBMDMs 5 h after addition of gentamycin (30 min of contact). Results are expressed as % of survival (CFUs at 5 h versus 30 min in *stat6*$^{-/-}$ cells normalised to the results obtained in wild-type macrophages set to 100%). Cells were treated with DMSO vehicle, or 2-deoxyglucose (2DG, 3 μM), oligomycin (3 μM), etomoxir (50 μM) 2 h before infection and maintained thought.

J Immunofluorescence confocal microscopy of the co-localisation of Kp52145 harbouring pFPV25.1Cm, and cresyl violet dye in wild-type macrophages treated with DMSO vehicle solution (control) or 2DG. The images were taken 90 min post infection. Images are representative of duplicate coverslips of three independent experiments.

K Percentage of Kp52145 harbouring pFPV25.1Cm co-localisation with cresyl violet over a time course. Wild-type iBMDMs treated with DMSO vehicle solution (control) or 2DG. were infected; coverslips were fixed and stained at the indicated times. Values are given as mean percentage of Kp52145 co-localising with the marker ± SEM. The number of infected cells counted per time in three independent experiments are indicated in the figure.

Data information: Error bars are presented as the mean ± SEM of three independent experiments in duplicate. Images are representative of three independent experiments. In panels (D–H) and (K), unpaired *t* test was used to determine statistical significance. In all the other panels, statistical analysis were carried out using one-way ANOVA with Bonferroni contrast for multiple comparisons test. ****$P \leq 0.0001$; ***$P \leq 0.001$; **$P \leq 0.01$; ns, $P > 0.05$ for the indicated comparisons.

©2022 The Authors

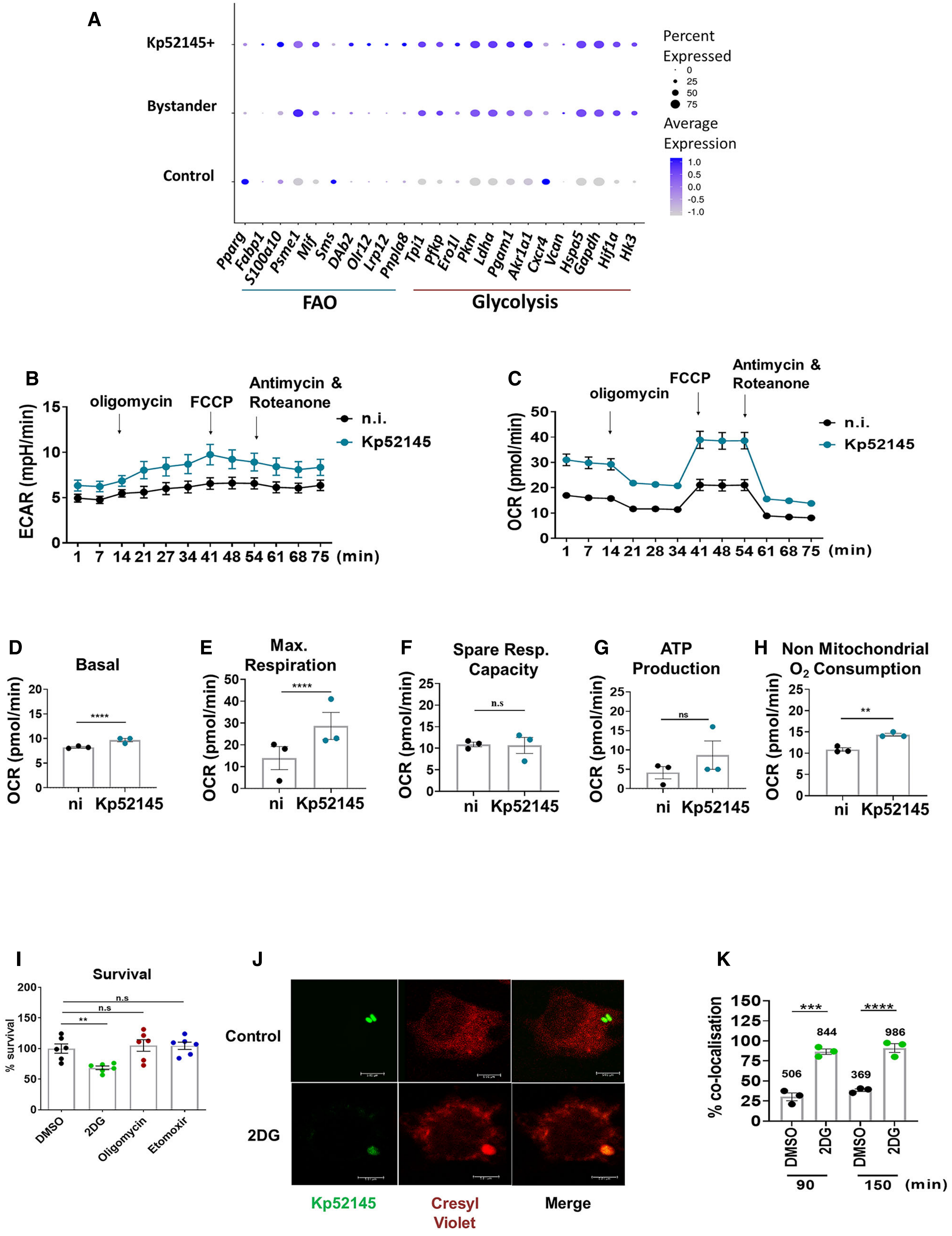

A
Kp52145+
Bystander
Control
Percent Expressed
0
25
50
75
Average Expression
1.0
0.5
0.0
-0.5
-1.0
Pparg
Fabp1
S100a10
Psme1
Mif
Sms
DAb2
Olr12
Lrp12
Pnpla8
Tpi1
Pfkp
Ero1l
Pkm
Ldha
Pgam1
Akr1a1
Cxcr4
Vcan
Hspa5
Gapdh
Hif1a
Hk3
FAO
Glycolysis
B
oligomycin
FCCP
Antimycin & Roteanone
ECAR (mpH/min)
n.i.
Kp52145
(min)
C
OCR (pmol/min)
D
Basal
E
Max. Respiration
F
Spare Resp. Capacity
G
ATP Production
H
Non Mitochondrial O_2 Consumption
ni
Kp52145
****
n.s
ns
**
I
Survival
% survival
DMSO
2DG
Oligomycin
Etomoxir
J
Control
2DG
Kp52145
Cresyl Violet
Merge
K
% co-localisation
506
844
369
986
***
90
150
(min)

Figure EV4.

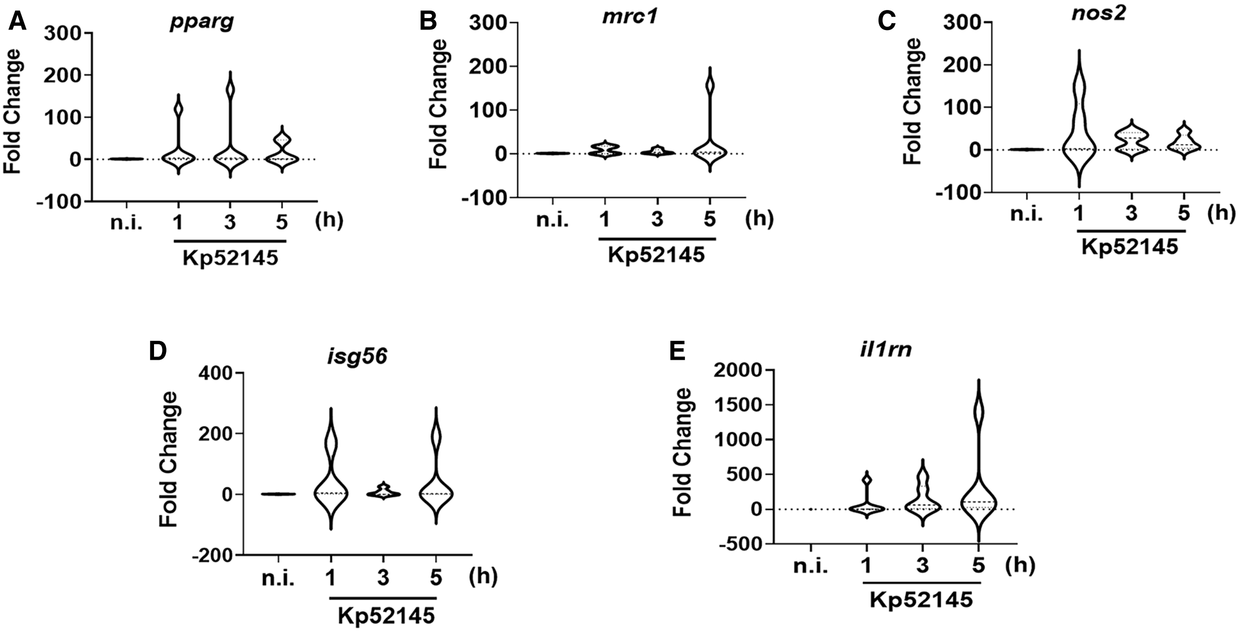

Figure EV5. ***Klebsiella pneumoniae*** **induces M(Kp) polarisation in human macrophages.**

A *pparg* mRNA levels were assessed by qPCR in hM-CSF-treated PBMCs from 6 donors non-infected (ni) or infected Kp52145 for 1, 3 or 5 h.

B *mrc1* mRNA levels were assessed by qPCR in hM-CSF-treated PBMCs from 6 donors non-infected (ni) or infected Kp52145 for 1, 3 or 5 h.

C *nos2* mRNA levels were assessed by qPCR in hM-CSF-treated PBMCs from 6 donors non-infected (ni) or infected Kp52145 for 1, 3 or 5 h.

D *isg56* mRNA levels were assessed by qPCR in hM-CSF-treated PBMCs from 6 donors non-infected (ni) or infected Kp52145 for 1, 3 or 5 h.

E *il1rn* mRNA levels were assessed by qPCR in hM-CSF-treated PBMCs from 6 donors non-infected (ni) or infected Kp52145 for 1, 3 or 5 h.

Data information: For all infections, after 1 h contact, medium replaced with medium containing gentamycin (100 μg/ml) to kill extracellular bacteria. Error bars are presented as the mean ± SEM of three independent experiments in duplicate.

©2022 The Authors
