# Supplementary material for: In vivo single‐cell transcriptomics reveal Klebsiella pneumoniae skews lung macrophages to promote infection
Source: EMBO Mol Med. 2022 Nov 7;14(12):e16888. doi: 10.15252/emmm.202216888 (PMC9727930; doi:10.15252/emmm.202216888)
Supplement: Supplementary file 6 — PDF+ [file EMMM-14-e16888-s007.pdf]

Article

TRANSPARENT PROCESS OPEN ACCESS

EMBO Molecular Medicine

In vivo single-cell transcriptomics reveal *Klebsiella pneumoniae* skews lung macrophages to promote infection

Amy Dumigan, Oisin Cappa, Brenda Morris, Joana Sá Pessoa, Ricardo Calderon-Gonzalez, Grant Mills, Rebecca Lancaster, David Simpson, Adrien Kissenpfennig & Jose A Bengoechea*

Abstract

The strategies deployed by antibiotic-resistant bacteria to counteract host defences are poorly understood. Here, we elucidate a novel host–pathogen interaction resulting in skewing lung macrophage polarisation by the human pathogen *Klebsiella pneumoniae*. We identify interstitial macrophages (IMs) as the main population of lung macrophages associated with *Klebsiella*. Single-cell transcriptomics and trajectory analysis of cells reveal type I IFN and IL10 signalling, and macrophage polarisation are characteristic of infected IMs, whereas Toll-like receptor (TLR) and Nod-like receptor signalling are features of infected alveolar macrophages. *Klebsiella*-induced macrophage polarisation is a singular M2-type we termed M(Kp). To rewire macrophages, *Klebsiella* hijacks a TLR-type I IFN-IL10-STAT6 axis. Absence of STAT6 limits *Klebsiella* intracellular survival and facilitates the clearance of the pathogen *in vivo*. Glycolysis characterises M(Kp) metabolism, and inhibition of glycolysis results in clearance of intracellular *Klebsiella*. Capsule polysaccharide governs M(Kp). *Klebsiella* also skews human macrophage polarisation towards M(Kp) in a type I IFN-IL10-STAT6-dependent manner. *Klebsiella* induction of M(Kp) represents a novel strategy to overcome host restriction, and identifies STAT6 as target to boost defences against *Klebsiella*.

Keywords IL10; *Klebsiella*; macrophage polarisation; STAT6; type I IFN
Subject Categories Immunology; Microbiology, Virology & Host Pathogen Interaction
DOI 10.15252/emmm.202216888 | Received 14 September 2022 | Revised 18 October 2022 | Accepted 19 October 2022 | Published online 7 November 2022
EMBO Mol Med (2022) 14: e16888

Introduction

Antibiotic resistance is a pandemic claiming more than 750,000 deaths per year. *Klebsiella pneumoniae* exemplifies the threat of this pandemic by the increasing number of strains resistant to fluoroquinolones, third-generation cephalosporins, aminoglycosides and even carbapenems (Penalva *et al*, 2019). These infections are associated with high mortality rates and prolonged hospitalisation (Giske *et al*, 2008). Not surprisingly, the World Health Organisation includes *K. pneumoniae* in the 'critical' group of pathogens for which new therapeutics are urgently needed.

Less obvious but critical for pathogenesis are *K. pneumoniae* adaptations to the human immune system allowing the pathogen to flourish in human tissues such as the airways. This is an aspect often overlooked because *K. pneumoniae* is not considered a pathogen able to manipulate the host cells because it does not encode type III or IV secretion systems known to deliver effectors into immune cells, or any of the toxins affecting cell biology. Of particular interest is to uncover whether *K. pneumoniae* deploys any strategy to manipulate macrophage function. These cells are crucial in host defence against infection by eliminating the invading pathogen via phagocytosis and the subsequent degradation in a phagolysosomal compartment, and by producing cytokines and chemokines following recognition of the pathogen to orchestrate the activation of other immune cells.

Macrophages show a remarkable plasticity allowing them to adapt to different microenvironments. Depending on the stimuli, macrophages broadly differentiate into type M1, pro-inflammatory showing potent microbicidal activity, M2 with immunomodulatory role to limit tissue damage and to control inflammation, M3 or 'switch' state (Malyshev & Lyamina, 2014) and M4 which is mediated by CXCL4 and observed in differentiated atherosclerotic plaque-associated macrophages (Gleissner *et al*, 2010). Different subsets of M2 macrophages have been identified, from M2a to M2d; all of them have in common the high-level expression of IL10 compared to M1 macrophages (Murray *et al*, 2014). Interestingly, increasing the number of M2 macrophages in the lungs as result of alcohol abuse or trauma is associated with increased susceptibility to *K. pneumoniae* infections (Dolgachev *et al*, 2012; Ohama *et al*, 2015; Tsuchimoto *et al*, 2015). On the contrary, there is an improvement in *K. pneumoniae* clearance when the M2 macrophage population is eliminated (Dolgachev *et al*, 2012; Ohama *et al*, 2015; Tsuchimoto *et al*, 2015), or after skewing macrophages towards an M1 state (Standiford *et al*, 2012). These clinical observations suggest

Wellcome-Wolfson Institute for Experimental Medicine, School of Medicine, Dentistry and Biomedical Sciences, Queen's University Belfast, Belfast, UK
*Corresponding author. Tel: +44 (0) 28 9097 6357; E-mail: j.bengoechea@qub.ac.uk

©2022 The Authors. Published under the terms of the CC BY 4.0 license.

a role for macrophage polarisation in *K. pneumoniae* infection biology, although this has not been investigated yet.

This work was designed to provide a comprehensive understanding of the *K. pneumoniae*–macrophage interface *in vivo*. We identify interstitial macrophages (IMs) as the main population of lung macrophages associated with *K. pneumoniae*. Single-cell transcriptomics uncover the programme induced by *K. pneumoniae* in infected and bystander alveolar macrophages (AMs) and IMs. Pathway analysis reveal a network involved in macrophage polarisation, and mechanistic studies demonstrate that *K. pneumoniae* exploits the immune effectors IL10 and type I IFNs to trigger a singular macrophage polarisation governed by the signal transducer and activator of transcription (STAT6) following the activation of TLR signalling. Inhibition of this pathway results in clearance of *K. pneumoniae*, illustrating the importance of macrophage polarisation in *K. pneumoniae* infection biology. Altogether, our work describes a new polarisation state induced by a human pathogen to overcome host restriction during pneumonia.

Results

Klebsiella pneumoniae is associated with interstitial macrophages and alveolar macrophages

In the mouse and human lungs, different populations of lung macrophages can be found including resident AMs and IMs. The latter includes two subsets populating distinct niches (Gibbings *et al*, 2017; Chakarov *et al*, 2019; Schyns *et al*, 2019). In response to insults and infection, monocytes can be recruited to the lung where the microenvironment shapes them to generate so-called tissue-resident alveolar macrophages (Misharin *et al*, 2017; Aegerter *et al*, 2020; Arafa *et al*, 2022). To determine to which main macrophage populations *K. pneumoniae* associates in the lungs of infected mice, C57BL/6 mice were infected intranasally with the clinical isolate *K. pneumoniae* CIP52.145 (hereafter Kp52145) (Nassif *et al*, 1989). This strain clusters within the KpI group that includes the strains associated with human infections (Lery *et al*, 2014; Holt *et al*, 2015). Moreover, Kp52145 encodes all the loci found in those strains associated with invasive community-acquired infections (Lery *et al*, 2014; Holt *et al*, 2015). To facilitate the detection of *K. pneumoniae in vivo*, Kp52145 was tagged with mCherry. The bacterial burden was 72-fold higher at 48 h post infection ($3.9 \times 10^7 \pm 7.0 \times 10^4$; 7 mice) than at 24 h post infection ($5.5 \times 10^4 \pm 2.9 \times 10^3$; 7 mice; $P < 0.0006$). Lungs were processed and stained for cytometric analysis as shown in Appendix Fig S1A and B. We excluded debris, doublets and dead cells. Monocytes (MNs) were identified as $Ly6C^+CD11b^+CD11c^-$, and AMs as $Ly6C^+CD11b^-CD11c^+$Siglec $F^{+,}$ which includes AMs whether of embryonic or monocytic origin. IMs were defined as $Ly6C^+CD11b^+CD11c^-SiglecF^-$, including all the subsets of IMs. The number of MNs, AMs and IMs did not change over time in PBS-treated mice (Fig 1A–C). The number of MNs was higher in Kp52145-infected mice at 48 h post infection than at 24 h (Fig 1A). The number of AMs was not significantly different between non-infected and infected mice (Fig 1B) which is consistent with previous findings (Ivin *et al*, 2017). In contrast, the number of IMs was significantly higher in infected mice than in PBS control animals (Fig 1C). The number of IMs was not significantly different at 48 and 24 h post infection (Fig 1C). Flow cytometric detection of mCherry-tagged Kp52145 showed that at 24 h post infection, 31% of IMs were associated with Kp52145, whereas 1% of MNs and 5% AMs were positive (Fig 1D). At 48 h post infection, MNs remained negative for *Klebsiella* whereas there was a significant increase in the percentage of AMs associated with Kp52145 reaching 15% (Fig 1D). The percentage of IMs associated with Kp52145 at 48 h was not significantly different to that at 24 h post infection (Fig 1D). Collectively, these results demonstrate that *in vivo K. pneumoniae* is associated with two distinct macrophage lineages, namely IMs and AMs.

To establish whether AMs and IMs are permissive or restrictive for *K. pneumoniae* survival, we reduced the numbers of each of them using clodronate-loaded liposomes (van Rooijen & Hendrikx, 2010), and determined the bacterial burden in the lungs. Intranasal instillation of liposomes reduced the number of AMs while increasing the number of IMs and neutrophils (Appendix Fig S1C–E). In these mice, the bacterial burden increased 3-log 24 h post infection (Fig 1E). In a parallel experiment, IMs were reduced using clodronate-loaded liposomes delivered intravenously with no impact on the AMs and neutrophils numbers (Appendix Fig S1F–H). Strikingly, the bacterial burden was reduced 17-fold in these mice (Fig 1F). These results show that AMs and IMs are differentially permissive to *K. pneumoniae* survival *in vivo*.

Single-cell RNA-seq reveals distinct transcriptomes in IM and AM populations during *Klebsiella pneumoniae* infection *in vivo*

We sought to characterise the transcriptomes of infected IMs and AMs in comparison to those of bystander IMs and AMs, with no bacteria associated, from infected mice, and to those of IMs and AMs from PBS-mock-infected mice. Mice were infected with mCherry-tagged Kp52145, and the IMs and AMs populations with and without associated bacteria were separated by FACS sorting (Fig EV1A). The gating strategy for cell sorting is outlined in Appendix Fig S1A and B. Single-cell mRNA sequencing (scRNA-seq) technology using the 10× Genomics platform was utilised to determine the transcriptome of the different populations of macrophages. The resulting dataset was curated using the Immgen20 open-source reference database (Benoist, 2016) to remove any non-macrophage cells from the data set, resulting in a total of 7,462 macrophages. 512 AMs and 1,113 IMs from PBS-treated mice, 3,080 AMs from infected mice, 2,281 with associated Kp52145 and 758 bystander AMs, and 2,797 IMs from infected mice, 2,273 with associated *Klebsiella* and 524 bystander cells. After normalisation of the samples, uniform manifold approximation and projection (UMAP) dimensionality reduction analysis of the combined samples revealed two clusters. One of the clusters comprised the IMs, characterised by the expression of the IM marker *cx3cr1* and being *siglecF* negative, whereas the other comprised the AMs, characterised by the expression of the marker *siglecF* (Fig EV1B). In the case of the IM cluster, it was possible to distinguish the cluster of cells from non-infected mice from those from infected mice which were further separated between bystander cells and those with associated bacteria (Fig EV1C). In contrast, there was no clear separation between the different populations of macrophages in the AM cluster (Fig EV1C).

Differential gene expression analysis revealed that 1,083 genes were differentially expressed in IMs from infected mice versus PBS-

 ©2022 The Authors

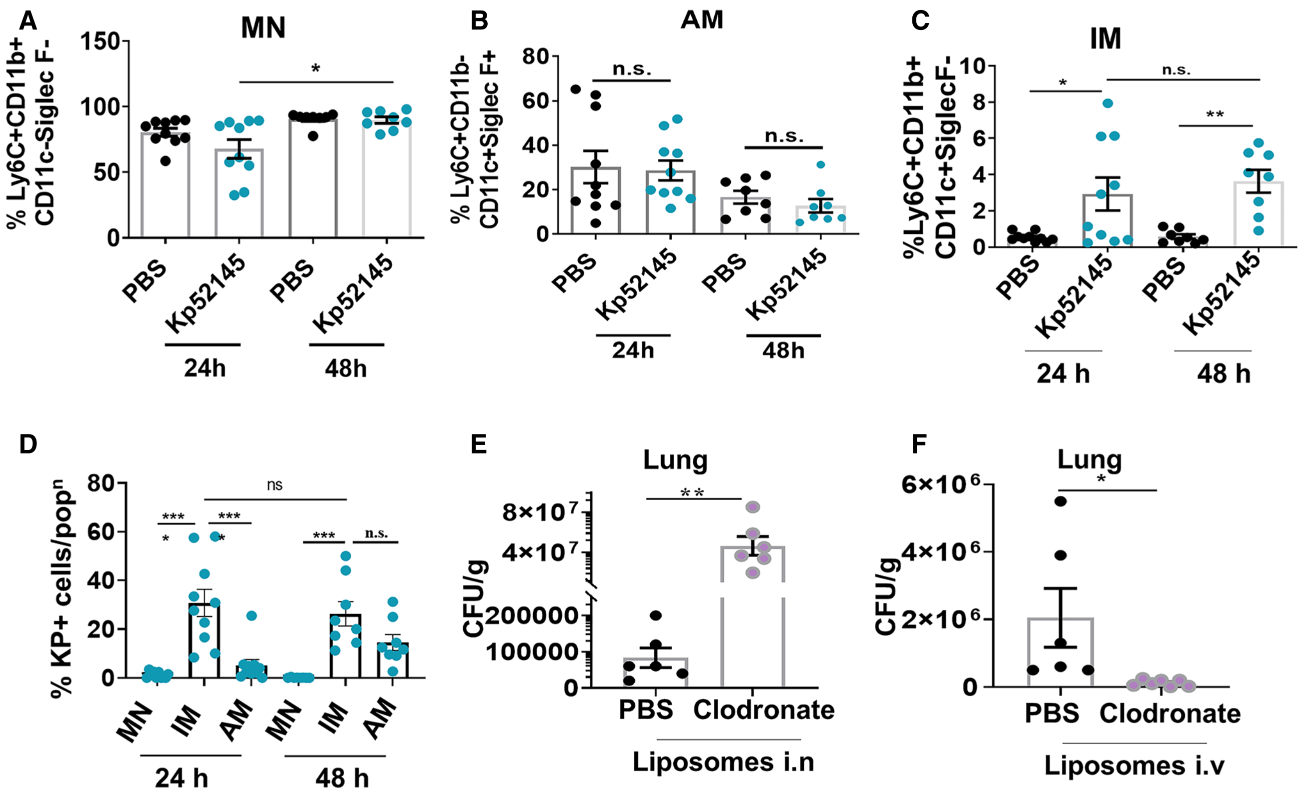

Figure 1. ***Klebsiella pneumoniae*** **is associated with interstitial macrophages and alveolar macrophages** ***in vivo*****.**

A–F Ten to twelve-week-old age- and sex-matched C57BL/6 mice were infected intranasally with mCherry tagged Kp52145. After 24 h or 48 h post-infection ($n = 10$/condition) lungs were harvested and processed for flow cytometric analysis to assess macrophages populations. (A) % Ly6C$^+$CD11b$^+$CD11c$^-$SiglecF$^-$ monocyte (MNs), (B) % Ly6C$^+$CD11b$^-$CD11c$^+$SiglecF$^+$ alveolar macrophage (AMs) and (C) % Ly6C$^+$CD11b$^+$CD11c$^+$SiglecF$^-$ interstitial macrophage (IMs) in Kp52145-infected animals compared to PBS controls. (D) Percentage of MN, AM and IM associated with Kp52145 from infected individual mice. (E) C57BL/6 mice were treated with control liposomes (PBS) or clodronate ones intranasally ($n = 6$ per group), and then infected with Kp52145. Bacterial burden in the lungs 24 h post infection was established by serial dilutions of lung homogenates on *Salmonella-Shigella* agar. (F) Liposomes were administered intravenously ($n = 6$ per group), and bacterial burden determined by plating 24 h post infection.

Data information: In all panels, each dot represents one animal. Values are presented as the mean ± SEM of two independent experiments. ****$P \leq 0.0001$; ***$P \leq 0.001$; **$P \leq 0.01$; *$P \leq 0.05$; ns, $P > 0.05$ for the indicated comparisons determined using One-way ANOVA.

mock infected mice. Of those, 393 genes were common between bystander and infected IMs, whereas 126 and 171 were only found in bystander cells and infected IMs, respectively. We compared the transcriptome of the different populations of IMs to identify signatures of infected and bystander cells. Eight hundred and ninety and 979 genes were differentially expressed in bystander and infected IMs versus PBS-mock infected IMs, respectively (Dataset EV1). Of them, 518 and 564 genes were upregulated, whereas 372 and 415 were down regulated in bystander and infected IMs versus PBS-mock infected IMs, respectively (Dataset EV1). To acquire insights into the biological processes of significance that characterise infected IMs and bystander cells, we performed gene set enrichment analysis (gProfiler) and then constructed network enrichment maps. When considering the downregulated genes, bystander and Kp52145-infected IMs shared an enrichment of networks related to translation (EBI BioStudies accession number S-BSST892). Pathways related to TGFβ signalling and Erbb4-Notch signalling were specific of bystander and Kp52145-infected IMs, respectively (EBI BioStudies accession number S-BSST892). In the case of the upregulated genes, there was an enrichment of pathways related to immune signalling in IMs from infected mice (Fig EV1D and E). It is notable the over representation of gene networks related to interferon signalling in bystander and infected IMs (Fig EV1D and E). This finding is consistent with an enrichment of motifs for transcriptional factors of the Irf family, and STAT1 in the promoter regions of the upregulated genes of IMs from infected mice as detected, interrogating the TRANSFAC database (Wingender *et al*, 1996). Only in Kp52145-infected IMs, we found gene networks involved in response to oxidative stress, starvation, iron uptake and macrophage polarisation, (*nos2*, *arg1*, *mrc1*/*cd206*; Fig EV1D). Interestingly, TRANSFAC-based analysis revealed an enrichment of the motif recognised by the transcriptional factor GKLF/Klf4 only in infected IMs. This transcriptional factor regulates M2 macrophage polarisation (Liao *et al*, 2011). Networks found only in bystander IMs included those related to TNF signalling, inflammasome activation and IL1 signalling, and C-type lectin receptors (Fig EV1E). Altogether, these results uncover that upregulation of IFN signalling and downregulation of translation are features of IMs following *K. pneumoniae* infection. The signature of infected IMs is the activation of networks connected to cellular stress and macrophage polarisation, whereas

networks connected to antimicrobial defence and sensing of infections are specific of bystander IMs.

Within the AM population, 218 genes were differentially expressed in Kp52145-associated AMs versus PBS-mock infected, whereas only 63 genes were differentially expressed in bystander AMs. (Dataset EV1). No networks were enriched within the downregulated genes of Kp52145-infected AMs, whereas four clusters were identified within the upregulated genes, receptor signalling cluster having the most nodes (EBI BioStudies accession number S-BSST892). Gene networks within this cluster are related to Toll-like receptor (TLR) and Nod-like receptor (NLR) signalling. TRANSFAC-based analysis showed that the motif recognised by the NF-κB transcriptional factor is enriched in the promoter region of the genes within this cluster. Connected to this cluster are the clusters of TNF signalling, and antigen presentation. Networks related to immune signalling include cytokines and chemokines, *il1b*, *tnfa*, *cxclc2*, *cxcl3* and calcium-dependent inflammatory proteins, *s100A9*, *s100A8*. No pathways were enriched in bystander AMs. Collectively, these results demonstrate a reduced activation of AMs following infection compared to IMs. The transcriptional pattern of infected AMs is related to TLR and NLR signalling-governed inflammation in an NF-κB-dependent manner.

To establish whether it is possible to construct a trajectory from non-infected to infected cells, we utilised Monocle analysis to determine the temporal pattern of gene expression over pseudotime. Whereas no distinct trajectory was observed in AMs (EBI BioStudies accession number S-BSST892), Monocle analysis revealed a clear trajectory in IMs from non-infected cells to bystander cells to Kp52145-infected cells (Fig EV1F). Seven modules of genes showing similar pattern of expression were identified (Dataset EV2). Genes in modules 3 and 4 were characteristic of Kp52145-infected IMs (Fig EV1G). Pathway analysis of modules 3 and 4, revealed an enrichment in pathways related to immune signalling, particularly type I IFN-stimulated genes (ISGs; *isg15*, *cxc10*, *ifit1*, *usp18*, *irgm1*, *irg1*) and IL10 signalling (*ptgs2*, *csf1*, *ptafr*), and IL4 signalling and macrophage polarisation (*nos2*, *arg1*, *lcn2*, *socs1*, *socs3*; Fig EV1H). STRING analysis seeking functional interactions between the 234 genes revealed two clusters (Fig EV1I). One cluster includes 39 genes associated with IFN signalling, and the other one encompasses 74 genes related to cytokine signalling and macrophage polarisation (Fig EV1I). TRANSFAC analysis showed that the regulatory regions of genes within modules 3 and 4 are characterised by motifs for transcriptional factors of the Irf family, and for the p65 subunit of NF-κB. Module 6, characteristic of bystander cells (Fig EV1G), is enriched with genes related to immune signalling, and IFN signalling (Fig EV1J). This module is characterised by the presence of binding motifs for Irf5 and Irf8 in the regulatory regions of the genes. Appendix Fig S2 shows the changes over pseudotime of selected top expressed genes within the modules 3, 4 and 6. Data demonstrates the increase in transcription of ISGs IFN (*isg15*, *cxcl10*, *irg1*, *ifi203*, *mndA*, *ifi205*), IL10 signalling (*ptgs2*, *ptafr*), IL4 signalling and macrophage polarisation (*nos2*, *arg1*, *lcn2*, *socs1*, *socs3*) from non-infected to bystander to infected cells. In contrast, there is a decrease in transcription from non-infected cells to bystander to infected cells of genes related to translation (*rnas6*, *rpsA*, *rps21*) and some others related to migration of immune cells and activation (*pparg*, *ifngr1*, *cytip*, *lsp1*).

Genes in modules 1 and 7 were upregulated in IMs from non-infected mice (Fig EV1G). Enrichment map analysis of modules 1 and 7 using gProfiler revealed one cluster related to regulation of translation (EBI BioStudies accession number S-BSST892).

Altogether, this analysis revealed that IFN and IL10 signalling governed by Irf and NF-κB transcriptional factors is characteristic of *K. pneumoniae*-infected IMs, whereas Irf-controlled IFN signalling is characteristic of bystander IMs. Furthermore, our data suggested that *K. pneumoniae* infection skews macrophage polarisation.

Klebsiella pneumoniae induces a singular polarisation state in interstitial and alveolar macrophages

The fact that one of the features of Kp52145-infected macrophages was the upregulation of genes associated with macrophage polarisation led us to interrogate further the scRNA-seq data set to assess the expression of genes associated with macrophage polarisation. Heat map analysis revealed an upregulation of several M1-related genes in bystander and infected IMs including *il1b*, *il12b*, *cd38*, *cxcl10*, *cxcl2 and nos2* (Fig EV2A). However, the expression of M2-related genes was higher than that of M1 genes (Fig EV2A). M2 upregulated genes included *msr1*, *cxcl16*, *egr2*, *arg1*, *il1rn*, *mmp14*, *ccr1*, *ccr2*, *clec4b* and *parp14* (Fig EV2A). Quantitative expression analysis showed that the expression of M2 genes was significantly higher than that of M1 genes in bystander and infected IMs (Fig EV2B). No differences were observed in the expression of M1 and M2 genes in IMs from mock-infected mice (Fig EV2B). Kp52145-induced polarisation has features of several of M2 subsets. *Arg1*, *il1rn*, *il10* and *fizz1*are found in M2a cells, *il10*, *tnfa*, *il6* and *il12* are typical of M2b cells, *cd163*, *cd206*, *il10* and *arg1* are characteristic of M2c macrophages, whereas *il10* and *nos2* are found in M2d cells (Murray *et al*, 2014). A similar picture was observed in AMs (EBI BioStudies accession number S-BSST892) although the number of upregulated genes was reduced compared to IMs.

Next, we carried out flow cytometry experiments interrogating infected and bystander IMs and AMs to confirm the scRNA-seq observations. The M2 markers Arg1, Fizz1 and CD163 were upregulated in Kp52145-infected IMs and AMs but not in bystander cells (Fig EV2C–E), uncovering the importance of macrophage-*Klebsiella* contact for macrophage polarisation.

Altogether, these results suggest that *K. pneumoniae* induces a singular polarisation in IMs and AMs consistent with M2 polarisation state. Following international guidelines on macrophage polarisation (Murray *et al*, 2014), we term this macrophage polarisation as M(Kp) because it cannot be ascribed to any of the known M2 subsets. scRNA-seq data, including pathway analysis and pseudotime data, and the flow cytometry experiments showed that M(Kp) is characterised by the increased expression of Arg1, Fizz1, iNOS, CD163, *cd206*, type I IFN and IL10 signalling-regulated genes, and the decreased expression of *pparγ*, and inflammatory markers.

Klebsiella pneumoniae-induced M(Kp) polarisation is STAT6 dependent

We next sought to provide mechanistic insights into the signalling pathway(s) governing M(Kp) polarisation. To facilitate this research, we questioned whether *K. pneumoniae* triggers M(Kp) polarisation in immortalised bone marrow-derived macrophages (iBMDMs). These cells are widely used to investigate immune signalling and macrophage polarisation. Consistent with the *in vivo*

 ©2022 The Authors

scRNA-seq results, infection of iBMDMs resulted in the upregulation of *arg1* and Arg1 (Appendix Fig S3A and B), *fizz1* and Fizz1 (Appendix Fig S3C and D), *pparγ*, *nos2*, *il12*, *il6* and *tnfa* (Appendix Fig S3F–I). *Il10* levels were also upregulated in infected iBMDMs (Appendix Fig S3J). The increased expression of *il10* was consistent with the increased phosphorylation of the IL10-governed transcriptional factor STAT3 in Kp52145-infected macrophages (Appendix Fig S3K). We have previously demonstrated the upregulation of type I IFN-dependent genes in *Klebsiella*-infected iBMDMs (Ivin *et al*, 2017). Altogether, these results demonstrate that infection of iBMDMs recapitulates the *in vivo K. pneumoniae*-induced macrophage polarisation.

The transcriptional factor STAT6 controls the transcription of M2-specific genes (Pauleau *et al*, 2004; Szanto *et al*, 2010). Therefore, we sought to determine whether STAT6 governs *K. pneumoniae*-induced M(Kp) polarisation. We first investigated whether Kp52145 induced the phosphorylation of STAT6 because this is a prerequisite for nuclear localisation and DNA binding of STAT6 (Goenka & Kaplan, 2011). Immunoblotting experiments confirmed that Kp52145 induced STAT6 phosphorylation (Fig 2A). STAT6 cooperates with KLF4 to regulate M2 macrophage (Liao *et al*, 2011). Kp52145 also increased the expression of *klf4* and KLF4 in macrophages (Fig 2B and C). To connect mechanistically STAT6 activation and *K. pneumoniae*-induced M(Kp) polarisation, we infected *stat6*$^{-/-}$ mice and assessed the expression of M(Kp) markers. Arg1 and Fizz1 were not upregulated in IMs with associated bacteria from *stat6*$^{-/-}$-infected mice (Fig 2D and E), whereas the levels of iNOS were upregulated in infected IMs from *stat6*$^{-/-}$ mice (Fig 2F).

To sustain further that STAT6 governs *K. pneumoniae*-induced M(Kp) polarisation, we infected *stat6*$^{-/-}$ macrophages and assessed the expression of M(Kp) markers. Fig 2G and H show that *arg1* and Arg1 levels were decreased in infected *stat6*$^{-/-}$ macrophages compared to infected wild-type cells. Furthermore, Kp52145 did not upregulate the expression of *il10*, *klf4*, *pparg* and *fizz1* in *stat6*$^{-/-}$ macrophages (Fig 2I; Appendix Fig S4A–C). In contrast, the expressions of *nos2*, *tnfa*, *il12*, *il6* were higher in infected *stat6*$^{-/-}$ macrophages than in wild-type cells (Appendix Fig S4D–G). The levels of *isg15* were not significantly between infected wild-type and *stat6*$^{-/-}$ macrophages (Appendix Fig S4H). Flow cytometry experiments using mCherry-tagged Kp52145 demonstrated that neither Arg1 nor CD206 were upregulated in infected *stat6*$^{-/-}$ macrophages in contrast to wild-type cells with associated bacteria (Appendix Fig S4I and J). In contrast, the levels of MHC-II, a well-established M1 marker, were significantly upregulated in *stat6*$^{-/-}$-infected macrophages compared to wild-type cells (Appendix Fig S4K). Similar results were obtained when *K. pneumoniae*-induced STAT6 activation was supressed using the STAT6 inhibitor AS1517499 (Nagashima *et al*, 2007). When infections were performed in the presence of AS1517499, Kp52145 did not upregulate the expression of *arg1*, *il10* and *fizz1* (EBI BioStudies accession number S-BSST892). In contrast, the expression of *nos2*, and *il12* were upregulated following infection (EBI BioStudies accession number S-BSST892). AS1517499 did not affect *Klebsiella*-induced *isg15* (EBI BioStudies accession number S-BSST892) in line with *stat6*$^{-/-}$-infected cells. Collectively, these results demonstrate that STAT6 acts as a key regulator of *K. pneumoniae*-induced M(Kp) polarisation *in vitro* and *in vivo*. Furthermore, in the absence of STAT6 *K. pneumoniae* induces a macrophage phenotype consistent with M1 polarisation.

We next sought to establish whether *K. pneumoniae* multidrug-resistant strains also trigger M(Kp) macrophage polarisation. *K. pneumoniae* strains NJST258-1, NJST258-2, KP35 and SHG10 induced the phosphorylation of STAT6 (Fig EV3A and B), indicating that *K. pneumoniae* activation of STAT6 is not strain dependent. NJST258-1, NJST258-2 and KP35 cluster within the epidemic clonal group ST258 producing the *K. pneumoniae* carbapenemase, and SGH10 belongs to the clonal group CG23 causing liver abscesses (Deleo *et al*, 2014; Ahn *et al*, 2016; Lam *et al*, 2018). Experiments infecting wild-type mice with mCherry-tagged bacteria showed that KP35 upregulated the expression of the M(Kp) markers Arg1 and CD206 in AMs and IMs with associated bacteria, and CD206 also in bystander AMs (Fig EV3C and D). *In vitro*, KP35 did not increase the levels of Arg1 in *stat6*$^{-/-}$ macrophages (Fig EV3E). Flow cytometry experiments using mCherry-tagged KP35 demonstrated that CD206 was not upregulated in infected *stat6*$^{-/-}$ macrophages (Fig EV3F), whereas the levels of MHC-II were upregulated (Fig EV3G). Altogether, these findings establish that the induction of M(Kp) is also a feature of multidrug-resistant *K. pneumoniae* strains.

Klebsiella pneumoniae **activation of STAT6 promotes infection**

To establish the importance of *K. pneumoniae*-induced activation of STAT6 on *K. pneumoniae* infection biology, we first investigated whether STAT6 contributes to *K. pneumoniae* subversion of cell-intrinsic immunity. We asked whether absence of STAT6 impairs *K. pneumoniae* intracellular survival. While no differences were observed in the adhesion of Kp52145 to *stat6*$^{-/-}$ and wild-type macrophages (Fig 3A), the phagocytosis of Kp52145 was reduced in *stat6*$^{-/-}$ macrophages compared to wild-type cells (Fig 3B). Time course experiments showed that the intracellular survival of Kp52145 was diminished in *stat6*$^{-/-}$ macrophages (Fig 3C). Previously, we have demonstrated that *K. pneumoniae* manipulates the traffic of the phagosome following phagocytosis to create a vacuole that does not fuse with lysosomes, the KCV, allowing the intracellular survival of *Klebsiella* (Cano *et al*, 2015). We then sought to determine whether the reduced intracellular survival observed in *stat6*$^{-/-}$ cells was due to an increase co-localisation of lysosomes with the KCV. Lysosomes were labelled with the membrane-permeant fluorophore cresyl violet (Ostrowski *et al*, 2016), and cells were infected with GFP-labelled Kp52145 to assess the KCV at the single-cell level by immunofluorescence. Confocal microscopy experiments revealed that the majority of the KCVs from wild-type macrophages did not co-localise with cresyl violet (Fig 3D and E), corroborating our previous work (Cano *et al*, 2015). In contrast, there was an increase co-localisation of the KCV from *stat6*$^{-/-}$ macrophages with the marker cresyl violet (Fig 3D and E), demonstrating that the absence of STAT6 results in the fusion of the KCV with lysosomes with a concomitant reduction in the numbers of intracellular bacteria.

To obtain a global view of the role of STAT6 in *K. pneumoniae* infection biology, we examined the ability of *stat6*$^{-/-}$ mice to control *K. pneumoniae* infection. At 24 h post infection, the bacterial loads in the lungs of *stat6*$^{-/-}$ mice were 28-fold lower than those of wild-type mice (Fig 3F), and there was limited dissemination to the spleens (Fig 3G). We obtained similar results when we tested the

©2022 The Authors

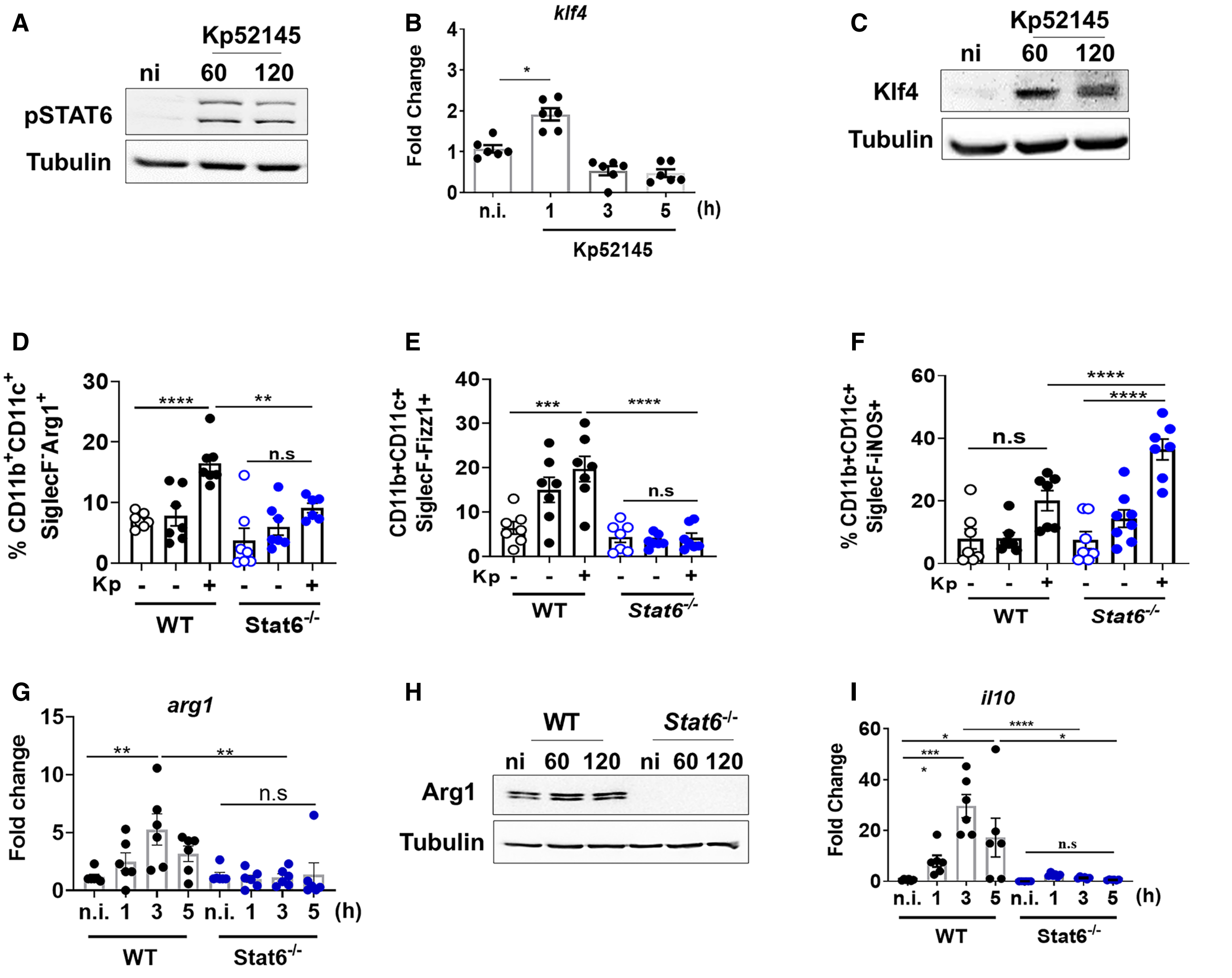

Figure 2. ***Klebsiella pneumoniae*-induced M(Kp) polarisation is STAT6 dependent.**

A Immunoblot analysis of the levels of the indicated proteins in lysates from non-infected (ni) and infected wild-type iBMDMs for 60 or 120 min.

B *klf4* mRNA levels were assessed by qPCR in wild-type iBMDMs infected with Kp52145 for 1, 3 or 5 h.

C Immunoblot analysis of Klf4 and tubulin levels in lysates from non-infected (ni) and infected wild-type iBMDMs for 60 or 120 min.

D–F Analysis by flow cytometry of the levels of M(Kp) markers expressed by IMs from PBS-mock-infected mice (black dots), and by IMs from infected mice (blue dots) with and without associated Kp52145. (D) Percentage of positive cells for Arg1. (E) Percentage of positive cells for Fizz1. (F) Percentage of positive cells for iNOS.

G *arg1* mRNA levels were assessed by qPCR in wild-type and *stat6*$^{-/-}$ iBMDMs non-infected (ni) or infected with Kp52145 for 1, 3 or 5 h.

H Immunoblot analysis of Arg1 and tubulin levels in lysates from non-infected (ni) and infected wild-type and *stat6*$^{-/-}$ iBMDMs for 60 or 120 min.

I *il10* mRNA levels were assessed by qPCR in wild-type (WT) and *stat6*$^{-/-}$ iBMDMs non-infected (ni) or infected with Kp52145 for 1, 3 or 5 h.

Data information: For all infections, after 1 h contact, medium replaced with medium containing gentamycin (100 µg/ml) to kill extracellular bacteria. qPCR and flow cytometry values are presented as the mean ± SEM of three independent experiments measured in duplicate. Images are representative of three independent experiments. ****$P \leq 0.0001$; ***$P \leq 0.001$; **$P \leq 0.01$; *$P \leq 0.05$; ns, $P > 0.05$ for the indicated comparisons using one way-ANOVA with Bonferroni contrast for multiple comparisons test.

effect of the STAT6 inhibitor AS1517499 on the ability of wild-type mice to control *K. pneumoniae* infection. The bacterial loads in the lungs of mice pre-treated with AS1517499 were significantly lower than those of mice pre-treated with the vehicle solution (Fig 3H). Bacterial loads in the spleens were not significantly different between the two groups of mice (Fig 3I). Altogether, these results establish the importance of STAT6 activation for *K. pneumoniae* survival *in vivo*.

Klebsiella pneumoniae-induced M(Kp) polarisation is governed by TLR2 and TLR4 signalling

We next sought to identify the signalling pathway(s) utilised by *K. pneumoniae* to activate STAT6 to induce M(Kp) polarisation. Work of our laboratory demonstrates that *K. pneumoniae* manipulates pattern recognition receptors (PRRs) as a virulence strategy to control inflammation (Sa-Pessoa *et al*, 2020). We then asked whether *K.*

 ©2022 The Authors

pneumoniae may exploit TLR signalling to activate STAT6 to induce M(Kp). Kp52145 did not induce the phosphorylation of STAT6 in *tlr2*$^{-/-}$, *tlr4*$^{-/-}$ and *tlr2/tlr4*$^{-/-}$ macrophages (Fig 4A). As expected, Kp52145 did not increase the levels of Arg1 (Fig 4B), *arg1* (Fig 4C) and *fizz1* (Fig 4D) in *tlr2*$^{-/-}$, *tlr4*$^{-/-}$ and *tlr2/4*$^{-/-}$ macrophages. The expressions of *il10*, *nos2* and the ISGs *isg15*, and *mx1* were only upregulated in *tlr2*$^{-/-}$ macrophages following infection (Fig 4E, and Appendix Fig S5A–C), indicating that TLR4 controls the levels of these M(Kp) markers. In contrast, TLR2 controls the expression of *pparγ* because Kp52145 did not upregulate *pparγ* in *tlr2*$^{-/-}$ and *tlr2/*

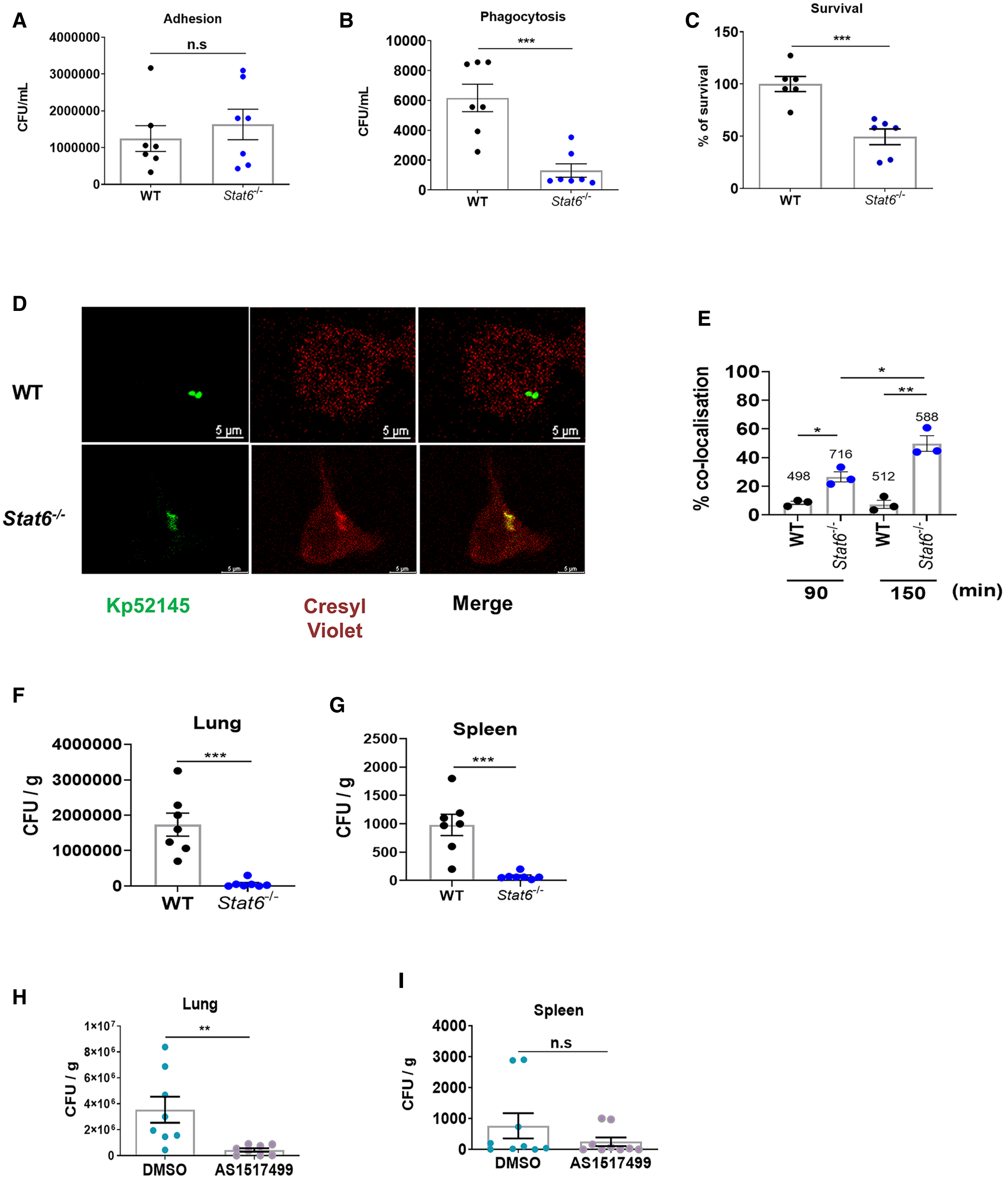

Figure 3.

Figure 3. STAT6 promotes *Klebsiella pneumoniae* infection.

A Kp52145 adhesion to wild-type (WT) and *stat6*$^{-/-}$ iBMDMs. Cells were infected with Kp52145 for 30 min, washed, cell lysed with saponin and bacteria quantified after serial dilution followed by plating on LB agar plates.

B Phagocytosis of Kp52145 by wild-type (WT) and *stat6*$^{-/-}$ iBMDMs. Cells were infected for 30 min, wells were washed, and it was added medium containing gentamicin (100 µg/ml) to kill extracellular bacteria. After 30 min, cells were washed, cell lysed with saponin and bacteria quantified after serial dilution followed by plating on LB agar plates.

C Kp52145 intracellular survival in wild-type (W)T and *stat6*$^{-/-}$ 5 h after addition of gentamycin (30 min of contact). Results are expressed as % of survival (CFUs at 5 h versus 30 min in *stat6*$^{-/-}$ cells normalised to the results obtained in wild-type macrophages set to 100%).

D Immunofluorescence confocal microscopy of the co-localisation of Kp52145 harbouring pFPV25.1Cm, and cresyl violet dye in wild-type (WT) and *stat6*$^{-/-}$ macrophages. The images were taken 90 min post infection. Images are representative of duplicate coverslips of three independent experiments.

E Percentage of Kp52145 harbouring pFPV25.1Cm co-localisation with cresyl violet over a time course. Values are given as mean percentage of Kp52145 co-localising with the marker ± SEM. The number of infected cells counted per time in three independent experiments are indicated in the figure.

F Bacterial burden in the lungs of Kp52145-infected wild-type and *stat6*$^{-/-}$ mice ($n = 7$ per group) 24 h post infection. Each dot represents one animal.

G Bacterial dissemination assessed by quantifying CFUs in the spleens of the same mice as in F.

H C56BL/6 mice were treated 24 h prior to infection with the STAT6 inhibitor AS1517499 (10 mg/kg in 200 µl volume by i.p) and 6 h post infection (5 mg/kg in 30 µl volume intranasally) or vehicle control DMSO ($n = 8$ per group). Bacterial burden was established by serial dilutions of lung homogenates on *Salmonella-Shigella* agar. Each dot represents one animal.

I Bacterial dissemination assessed by quantifying CFUs in the spleens from the same infected mice treated with AS1517499 or vehicle control DMSO.

Data information: Values are presented as the mean ± SEM of three independent experiments measured in duplicate. ***$P \leq 0.001$; **$P \leq 0.01$; *$P \leq 0.05$; ns, $P > 0.05$ for the indicated comparisons determined using unpaired *t* test.

tlr4$^{-/-}$ macrophages (Appendix Fig S5D). Altogether, these results demonstrate that *K. pneumoniae*-induced M(Kp) polarisation is TLR2 and TLR4 dependent.

The TLR adaptors MyD88, TRAM and TRIF govern *Klebsiella pneumoniae*-induced M(Kp) polarisation

TLR signalling involves a series of different adaptors. Myeloid-Differentiation factor-88 (MyD88) is a universal adaptor used by all TLRs except TLR3, Toll/IL-1R domain-containing adaptor-inducing IFN-β (TRIF) is used by TLR3 and TLR4, whereas TRIF-related adaptor molecule (TRAM) is recruited by endosomal located TLR and TLR4 (O'Neill & Bowie, 2007). Therefore, we asked whether MyD88, TRAM and TRIF are required to induce the M(Kp) polarisation. Phosphorylation of STAT6 was not detected in infected *myd88*$^{-/-}$, and *tram/trif*$^{-/-}$ macrophages (Fig 4F). As anticipated Arg1 (Fig 4G), *arg1* (Fig 4H) and *fizz1* (Fig 4I) were not upregulated in infected *myd88*$^{-/-}$, and *tram/trif*$^{-/-}$ macrophages. Kp52145 induction of *il10* was MyD88-dependent because Kp52145 upregulated *il10* only in *tram/trif*$^{-/-}$ macrophages (Fig 4J). The expressions of *pparg* was abrogated in the absence of MyD88 and TRAM/TRIF (Appendix Fig S5E). In addition, *isg15* and *mx1* were not upregulated in infected *tram/trif*$^{-/-}$ macrophages (Appendix Fig S5F and G), which is consistent with our recent evidence demonstrating that *K. pneumoniae* induces type I IFNs and ISGs in a TRAM-TRIF-dependent manner (Ivin *et al*, 2017). Kp52145 did not upregulate *nos2* in *tram/trif*$^{-/-}$ macrophages (Appendix Fig S5H), which is in agreement with *nos2* being an ISG (Farlik *et al*, 2010). In contrast, *nos2* levels were upregulated in infected *myd88*$^{-/-}$ macrophages (Appendix Fig S5H). Altogether, these findings establish that *K. pneumoniae*-induced M(Kp) is MyD88, TRAM and TRIF dependent.

Klebsiella pneumoniae exploits type I IFN signalling to induce M (Kp) polarisation

Given that TLR4-TRAM-TRIF pathway is essential for M(Kp) activation, that this pathway controls type I IFN signalling in *K. pneumoniae* infections (Ivin *et al*, 2017), and that type I IFN signalling is a feature of IMs following *Klebsiella* infection, we decided to elucidate whether *K. pneumoniae* exploits type I IFN signalling to induce M(Kp) polarisation. To address this question, we infected type I IFN receptor-deficient (*ifnar1*$^{-/-}$) macrophages and assessed M(Kp) markers. Kp52145 did not induce the phosphorylation of STAT6 in *ifnar1*$^{-/-}$ macrophages (Fig 5A). As expected, Arg1 and *arg1* were not upregulated in Kp52145-infected *ifnar1*$^{-/-}$ cells (Fig 5B and C). Similar result was observed for *nos2*, *pparγ* and *fizz1* (Fig 5D–F). In contrast, Kp52145 still upregulated *il10* in *ifnar1*$^{-/-}$ macrophages (Fig 5G). Flow cytometry experiments showed that Kp52145 did not upregulate the expression of Arg1 (Fig 5H), and CD206 in *ifnar1*$^{-/-}$ cells (Fig 5I), whereas the expression of MHC-II was higher in *ifnar1*$^{-/-}$ macrophages than in wild-type cells following infection (Fig 5J). Type I IFN stimulation alone did neither induce STAT6 phosphorylation nor upregulate Arg1 in wild-type cells (EBI BioStudies accession number S-BSST892).

Because Irf3 controls type I IFN production in *K. pneumoniae in vitro* and *in vivo* (Ivin *et al*, 2017), we postulated that Irf3 is required for *K. pneumoniae* induction of M(Kp). Indeed, Kp52145 did not phosphorylate STAT6 or induce Arg1 in *irf3*$^{-/-}$ macrophages (Fig 5K and L). As anticipated, *arg1* (Fig 5B), *pparγ* (Fig 5E) and *fizz1* (Fig 5F) levels were not increased in *irf3*$^{-/-}$ cells following infection, whereas the levels of *il10* were similar that those found in infected wild-type cells (Fig 5G). The fact that *nos2* levels were upregulated in infected *irf3*$^{-/-}$macrophages (Fig 5D) indicates that Irf3 does not control the transcription of this gene.

Collectively, these experiments demonstrate that *K. pneumoniae* leverages the immunomodulatory properties of type I IFN to induce M(Kp) polarisation.

IL10 is required for *Klebsiella pneumoniae*-governed M(Kp)

Our findings indicate that IL10 is one of the signatures of M(Kp). Therefore, we sought to identify the signalling pathways governing *K. pneumoniae*-induction of IL10. Our previous results revealed that TLR4-MyD88 signalling controls IL10 production following Kp52145

 ©2022 The Authors

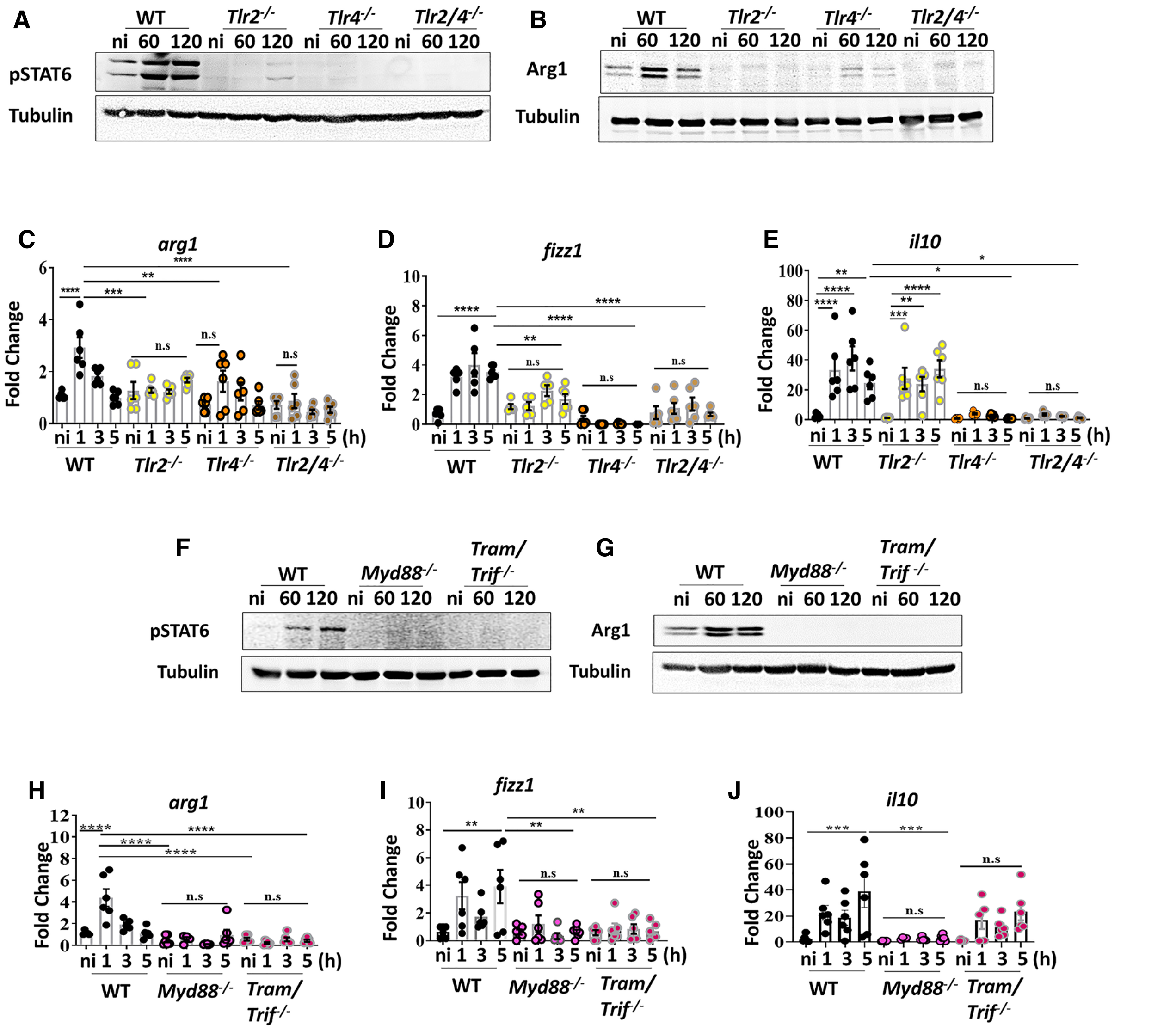

Figure 4. ***Klebsiella pneumoniae*-induced M (Kp) polarisation is dependent on TLR signalling and the TLR adaptors MyD88, TRAM and TRIF.**

A Immunoblot analysis of phospho-STAT6 (pSTAT6) and tubulin levels in lysates from non-infected (ni) and infected wild-type (WT), *tlr2*$^{-/-}$, *tlr4*$^{-/-}$ and *tlr2/4*$^{-/-}$ iBMDMs for 60 or 120 min.

B Immunoblot analysis of Arg1and tubulin levels in lysates from non-infected (ni) and infected wild-type (WT), *tlr2*$^{-/-}$, *tlr4*$^{-/-}$ and *tlr2/4*$^{-/-}$ iBMDMs for 60 or 120 min.

C *arg1* mRNA levels were assessed by qPCR in wild-type (WT) and *tlr2*$^{-/-}$, *tlr4*$^{-/-}$ and *tlr2/4*$^{-/-}$ iBMDMs non-infected (ni) or infected with Kp52145 for 1, 3 or 5 h.

D *fizz1* mRNA levels were assessed by qPCR in wild-type (WT) and *tlr2*$^{-/-}$, *tlr4*$^{-/-}$ and *tlr2/4*$^{-/-}$ iBMDMs non-infected (ni) or infected with Kp52145 for 1, 3 or 5 h.

E *il10* mRNA levels were assessed by qPCR in wild-type (WT) and *tlr2*$^{-/-}$, *tlr4*$^{-/-}$ and *tlr2/4*$^{-/-}$ iBMDMs non-infected (ni) or infected with Kp52145 for 1, 3 or 5 h.

F Immunoblot analysis of phospho-STAT6 (pSTAT6) and tubulin levels in lysates from non-infected (ni) and infected wild-type (WT), *myd88*$^{-/-}$, *tram/trif*$^{-/-}$ for 60 or 120 min.

G Immunoblot analysis of Arg1 and tubulin levels in lysates from non-infected (ni) and infected wild-type (WT), *myd88*$^{-/-}$, *tram/trif*$^{-/-}$ for 60 or 120 min.

H *arg1* mRNA levels were assessed by qPCR in wild-type (WT), *myd88*$^{-/-}$, *tram/trif*$^{-/-}$ non-infected (ni) or infected with Kp52145 for 1, 3 or 5 h.

I *fizz1* mRNA levels were assessed by qPCR in wild-type (WT), *myd88*$^{-/-}$, *tram/trif*$^{-/-}$ non-infected (ni) or infected with Kp52145 for 1, 3 or 5 h.

J *il10* mRNA levels were assessed by qPCR in wild-type (WT), *myd88*$^{-/-}$, *tram/trif*$^{-/-}$ non-infected (ni) or infected with Kp52145 for 1, 3 or 5 h.

Data information: For all infections, after 1 h contact, medium replaced with medium containing gentamycin (100 μg/ml) to kill extracellular bacteria. Error bars are presented as the mean ± SEM of three independent experiments in duplicate. Images are representative of three independent experiments. ****$P \leq 0.0001$; ***$P \leq 0.001$; **$P \leq 0.01$; *$P \leq 0.05$; ns, $P > 0.05$ for the indicated comparisons using one way-ANOVA with Bonferroni contrast for multiple comparisons test.

infection. The fact that the transcriptional factor CREB controls IL10 production in macrophages following TLR stimulation (Saraiva & O'Garra, 2010) led us to ascertain whether CREB governs *K. pneumonia*-induced IL10 production. The phosphorylation of CREB is a key event regulating its transcriptional activity (Mayr & Montminy, 2001). Immunoblotting experiments confirmed that

Kp52145 triggered the phosphorylation of CREB in wild-type macrophages (Fig 6A and B). However, CREB phosphorylation was reduced in infected *tlr4*$^{-/-}$ (Fig 6A) and *myd88*$^{-/-}$ (Fig 6B) macrophages. To connect CREB activation and IL10 production in *K. pneumoniae*-infected cells, we used a siRNA-based approach to knockdown CREB in macrophages. The efficiency of CREB knockdown in wild-type macrophages is shown in EBI BioStudies accession number S-BSST892. Kp52145 induction of *il10* was abrogated in CREB knockdown macrophages (Fig 6C), demonstrating the role of CREB activation in *K. pneumoniae* induction of IL10. Collectively, these experiments uncover that a TLR4-MyD88-CREB signalling pathway mediates *K. pneumoniae* induction of IL10.

To determine whether *K. pneumoniae* exploits the immunomodulatory properties of IL10 to skew macrophage polarisation, we infected *il10*$^{-/-}$ macrophages and assessed different M(Kp) markers. We did not detect the phosphorylation of STAT6 in infected *il10*$^{-/-}$ macrophages (Fig 6D). The levels of *arg1*, Arg1 were not upregulated in infected *il10*$^{-/-}$ macrophages (Fig 6E and F). The expressions of *pparγ* (Fig 6G) and *fizz1* (Fig 6H) were not upregulated in infected *il10*$^{-/-}$ macrophages. In contrast, the levels of *nos2*, *tnfα*, *mx1* and *isg15* were significantly increased in infected *il10*$^{-/-}$ macrophages compared to wild-type controls (Appendix Fig S6A–D). Flow cytometric analysis showed that Kp52145 did not increase Arg1 (Appendix Fig S6E) and CD206 (Appendix Fig S6F) in *il10*$^{-/-}$

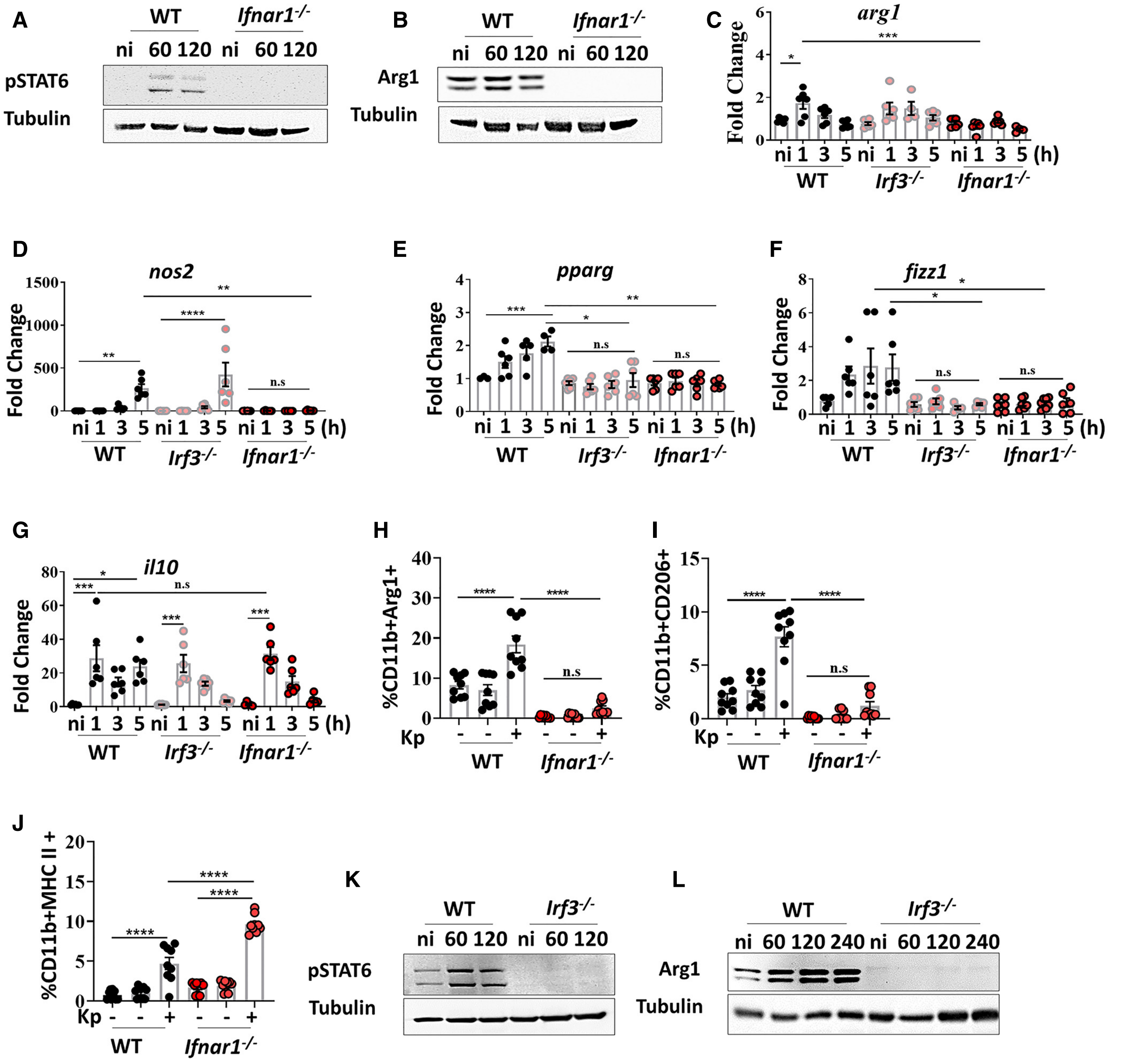

Figure 5.

 ©2022 The Authors

Figure 5. *Klebsiella pneumoniae* exploits type I IFN signalling to induce M(Kp) polarisation.

A Immunoblot analysis of phospho-STAT6 (pSTAT6) and tubulin levels in lysates from non-infected (ni) and infected wild-type (WT), or *ifnar1*$^{-/-}$ for 60 or 120 min.
B Immunoblot analysis of Arg1 and tubulin levels in lysates from non-infected (ni) and infected wild-type (WT), or *ifnar1*$^{-/-}$ for 60 or 120 min.
C *arg1* mRNA levels were assessed by qPCR in wild-type (WT), *irf3*$^{-/-}$, *ifnar1*$^{-/-}$ non-infected (ni) or infected with Kp52145 for 1, 3 or 5 h.
D *nos2* mRNA levels were assessed by qPCR in wild-type (WT), *irf3*$^{-/-}$, *ifnar1*$^{-/-}$ non-infected (ni) or infected with Kp52145 for 1, 3 or 5 h.
E *pparg* mRNA levels were assessed by qPCR in wild-type (WT), *irf3*$^{-/-}$, *ifnar1*$^{-/-}$ non-infected (ni) or infected with Kp52145 for 1, 3 or 5 h.
F *fizz1* mRNA levels were assessed by qPCR in wild-type (WT), *irf3*$^{-/-}$, *ifnar1*$^{-/-}$ non-infected (ni) or infected with Kp52145 for 1, 3 or 5 h.
G *il10* mRNA levels were assessed by qPCR in wild-type (WT), *irf3*$^{-/-}$, *ifnar1*$^{-/-}$ non-infected (ni) or infected with Kp52145 for 1, 3 or 5 h.
H Percentage of wild-type (WT) and *ifnar1*$^{-/-}$ iBMDMs with and without associated Kp52145 positive for Arg1 1, 3 or 5 h post infection. Kp52145 was tagged with mCherry.
I Percentage of wild-type (WT) and *ifnar1*$^{-/-}$ iBMDMs with and without associated Kp52145 positive for CD206 1, 3 or 5 h post infection. Kp52145 was tagged with mCherry.
J Percentage of wild-type (WT) and *ifnar1*$^{-/-}$ iBMDMs with and without associated Kp52145 positive for MHCII 1, 3 or 5 h post infection. Kp52145 was tagged with mCherry.
K Immunoblot analysis of phospho-STAT6 (pSTAT6) and tubulin levels in lysates from non-infected (ni) and infected wild-type (WT), or *irf3*$^{-/-}$ for 60 or 120 min.
L Immunoblot analysis of Arg1 and tubulin levels in lysates from non-infected (ni) and infected wild-type (WT), or *irf31*$^{-/-}$ for 60 or 120 min.

Data information: For all infections, after 1 h contact, medium replaced with medium containing gentamycin (100 μg/ml) to kill extracellular bacteria. Error bars are presented as the mean ± SEM of three independent experiments in duplicate. Images are representative of three independent experiments. ****$P \leq 0.0001$; ***$P \leq 0.001$; **$P \leq 0.01$; *$P \leq 0.05$; ns, $P > 0.05$ for the indicated comparisons using one way-ANOVA with Bonferroni contrast for multiple comparisons test.

macrophages, whereas the levels of MHC-II were higher in *il10*$^{-/-}$ macrophages, with and without bacteria, than in the wild-type ones (Appendix Fig S6G). Recombinant IL10 neither alone nor in combination with type I IFN induced the phosphorylation of STAT6 or the upregulation of Arg1 in wild-type macrophages (EBI BioStudies accession number S-BSST892). Altogether, these data indicate that IL10 is necessary for *K. pneumoniae*-induction of M(Kp).

Given the importance of IL10 in *K. pneumoniae*-macrophage interplay, we asked whether IL10 is required for *K. pneumoniae* intracellular survival. No differences were observed in the adhesion (Fig 6I) and phagocytosis (Fig 6J) of Kp52145 between wild-type and *il10*$^{-/-}$ macrophages. Assessment of the numbers of intracellular bacteria over time showed a 50% decrease of Kp52145 survival in *il10*$^{-/-}$ macrophages (Fig 6K). Confocal microscopy experiments showed an increase in the co-localisation of the KCV from *il10*$^{-/-}$ macrophages with cresyl violet (Fig 6L and M), demonstrating that the lack of IL10 results in the fusion of the KCV with lysosomes with a concomitant reduction in the numbers of intracellular bacteria.

In summary, our data demonstrates that *K. pneumoniae* exploits IL10 following the activation of a TLR4-MyD88-CREB pathway to induce M(Kp) polarisation. IL10 is crucial for *K. pneumoniae*-governed control of the phagosome maturation to survive inside macrophages.

Glycolysis characterises the M(Kp) metabolism

Metabolic reprogramming is a key aspect in the regulation of macrophage polarisation and function (Biswas & Mantovani, 2012; Jha

Figure 6. *Klebsiella pneumoniae*-induced M(Kp) polarisation is dependent on IL10.

A Immunoblot analysis of phospho-CREB (pCREB) and tubulin levels in lysates from non-infected (ni) and infected wild-type (WT), or *tlr4*$^{-/-}$ for 60 or 120 min.
B Immunoblot analysis of phospho-CREB (pCREB) and tubulin levels in lysates from non-infected (ni) and infected wild-type (WT), or *myd88*$^{-/-}$ for 60 or 120 min.
C *il0* mRNA levels were assessed by qPCR in iBMDMs transfected with All Stars siRNA control (AS), or CREB siRNA (CREBsi) non-infected (ni) or infected with Kp52145 for 3 h.
D Immunoblot analysis of phospho-STAT6 (pSTAT6) and tubulin levels in lysates from non-infected (ni) and infected wild-type (WT), or *il10*$^{-/-}$ for 60 or 120 min.
E *arg1* mRNA levels were assessed by qPCR in wild-type (WT), *il10*$^{-/-}$ non-infected (ni) or infected with Kp52145 for 1, 3 or 5 h.
F Immunoblot analysis of Arg1 and tubulin levels in lysates from non-infected (ni) and infected wild-type (WT), or *il10*$^{-/-}$ for 60 or 120 min.
G *pparg* mRNA levels were assessed by qPCR in wild-type (WT), *il10*$^{-/-}$ non-infected (ni) or infected with Kp52145 for 1, 3 or 5 h.
H *fizz1* mRNA levels were assessed by qPCR in wild-type (WT), *il10*$^{-/-}$ non-infected (ni) or infected with Kp52145 for 1, 3 or 5 h.
I Kp52145 adhesion to wild-type (WT) and *il10*$^{-/-}$ iBMDMs. Cells were infected with Kp52145 for 30 min, washed, cell lysed with saponin, and bacteria quantified after serial dilution followed by plating on LB agar plates.
J Phagocytosis of Kp52145 by wild-type (WT) and *il10*$^{-/-}$ iBMDMs. Cells were infected for 30 min, wells were washed, and it was added medium containing gentamicin (100 μg/ml) to kill extracellular bacteria. After 30 min, cells were washed, cell lysed with saponin and bacteria quantified after serial dilution followed by plating on LB agar plates.
K Kp52145 intracellular survival in wild-type (W)T and *il10*$^{-/-}$ 5 h after addition of gentamycin (30 min of contact). Results are expressed as % of survival (CFUs at 5 h versus 30 min in *stat6*$^{-/-}$ cells normalised to the results obtained in wild-type macrophages set to 100%).
L Immunofluorescence confocal microscopy of the co-localisation of Kp52145 harbouring pFPV25.1Cm, and cresyl violet dye in wild-type (WT) and *il10*$^{-/-}$ macrophages. The images were taken 90 min post infection. Images are representative of duplicate coverslips of three independent experiments.
M Percentage of Kp52145 harbouring pFPV25.1Cm co-localisation with cresyl violet over a time course in wild-type (WT) and *il10*$^{-/-}$ macrophages. Values are given as mean percentage of Kp52145 co-localising with the marker ± SEM. The number of infected cells counted per time in three independent experiments are indicated in the figure.

Data information: For all infections, after 1 h contact, medium replaced with medium containing gentamycin (100 μg/ml) to kill extracellular bacteria. Error bars are presented as the mean ± SEM of three independent experiments in duplicate. Images are representative of three independent experiments. In panels (C, P, Q and R) unpaired *t* test was used to determine statistical significance. In all the other panels, statistical analysis were carried out using one-way ANOVA with Bonferroni contrast for multiple comparisons test. ****$P \leq 0.0001$; ***$P \leq 0.001$; **$P \leq 0.01$; *$P \leq 0.05$; ns, $P > 0.05$ for the indicated comparisons.

©2022 The Authors

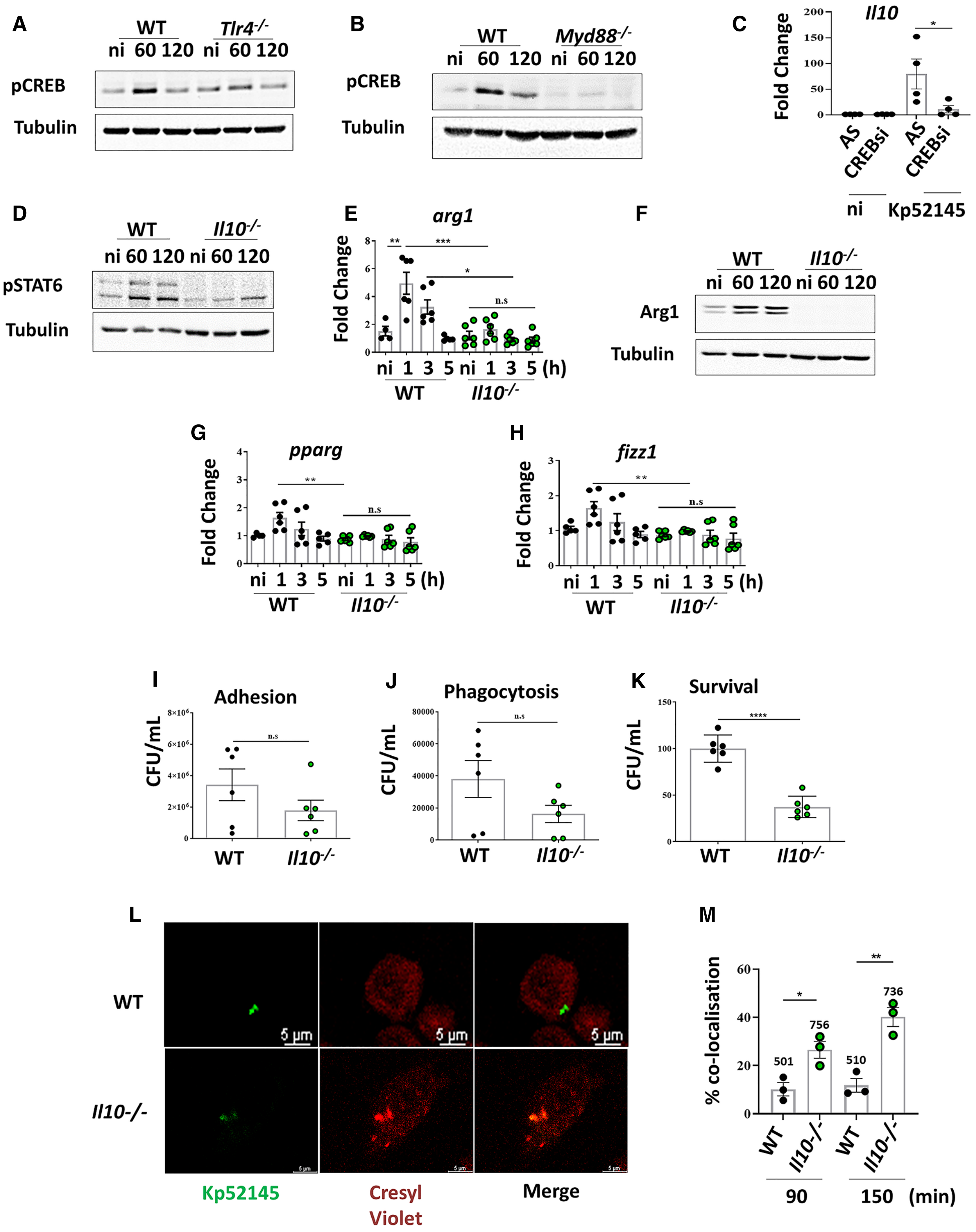

Figure 6.

et al, 2015). Therefore, we sought to characterise the metabolism associated with *K. pneumoniae*-induced M(Kp) polarisation. Depending on the stimuli received, macrophages can switch between an aerobic metabolism, based on oxidative phosphorylation, to an anaerobic one, based on glycolysis (Biswas & Mantovani, 2012). We interrogated the scRNA-seq data to reveal whether

©2022 The Authors

K. pneumonaie infection is associated with changes in the expression of metabolic genes. It has been established that metabolic changes in macrophages are associated with changes in the transcription of genes governing the different metabolic pathways (Jha *et al*, 2015). Dot plot analysis showed an upregulation of genes related to glycolysis in Kp52145-associated and bystander IMs including *Pfkp*, *Hif1a*, *HK3*, *gapdh* and *Ldha* (Fig EV4A). In contrast, the expression of genes related to FAO and OXPHOS was downregulated (Fig EV4A). In AMs, the glycolysis related genes *pgamI*, *HK3* and *hif1a* were also upregulated in bystander and infected cells (EBI BioStudies accession number S-BSST892). Taken together, these data suggest that glycolysis may characterise *K. pneumoniae*-triggered M(Kp) polarisation.

To provide experimental evidence on the metabolism linked to M(Kp), we monitored glycolysis parameters (glycolysis and glycolytic reserve) and mitochondrial function characteristics (basal mitochondrial respiration, ATP production, maximal respiration and spare respiratory capacity) in infected macrophages by measuring the extracellular acidification rate (ECAR) and the oxygen consumption rate (OCR) using a Seahorse XFe96 analyser. The metabolic profile of Kp52145 in response to glucose revealed a relative increase in ECAR and OCR (EBI BioStudies accession number S-BSST892). These observations are similar to those reported on the metabolic profile of the ST258 strain KP35 (Ahn *et al*, 2021). These changes in bacterial metabolism did not significantly alter the metabolic activity measured in infected macrophages (Fig EV4B and C). Infected macrophages showed an increase in ECAR (Fig EV4B), representing the glycolysis rate. Inhibition of the mitochondrial F_1F_O-ATPase with oligomycin resulted in a modest increase in ECAR (Fig EV4B). The lack of increase in ECAR following the addition of the ionophore FCCP, that uncouples mitochondrial respiration by increasing H+ transport across the inner mitochondrial membrane, and rotenone and antimycin A, inhibitors of mitochondrial complex I and III, respectively, indicates that the maximal glycolytic capacity was already reached (Fig EV4B). This result suggests that ATP production is mostly dependent on glycolysis in Kp52145-infected macrophages because carbon flux is coupled to glycolytic ATP production. The increase of OCR following Kp52145 infection reflected an increase in mitochondrial basal respiration (Fig EV4C and D). Addition of oligomycin triggered a decrease of cellular OCR; however, the OCR was still significantly higher in Kp52145-infected cells compared to non-infected ones indicating that not all oxygen consumption is used for ATP production in *K. pneumoniae*-infected cells (Fig EV4C). Subsequent addition of FCCP, which stimulates respiration, showed that the maximal respiration capacity was higher in Kp52145-infected macrophages than in non-infected ones (Fig EV4C and E). However, the spare respiratory capacity was not significantly different between Kp52145-infected macrophages and non-infected ones (Fig EV4F), indicating that *K. pneumoniae* infection does not deplete the cellular energy via an increased OXPHOS. No differences were found in ATP production between non-infected and Kp52145-infected cells (Fig EV4G). The fact that OCR was higher in Kp52145-infected cells as compared to non-infected ones after the addition of rotenone and antimycin A indicates that the non-mitochondrial respiration is increased in *K. pneumoniae*-infected macrophages (Fig EV4H). When ECAR and OCR analysis following infection were done in the presence of the STAT6 inhibitor AS1517499, we observed a reduction in both measurements (EBI BioStudies accession number S-BSST892), connecting *Klebsiella*-induced metabolism with STAT6 activation. Altogether, these results are consistent with the model in which glycolysis is characteristic of *K. pneumoniae*-induced M(Kp) without impairment of mitochondrial bioenergetics.

To determine the effect of *K. pneumoniae*-induced metabolism on *Klebsiella*-macrophage interface, we used 2-doxyglucose (2DG) to inhibit glycolysis, and oligomycin and etomoxir to inhibit OXPHOS. Control experiments showed that the drugs did not affect the growth of Kp52145 (Appendix Fig S7A). The drugs did not affect the adhesion of Kp52145 to macrophages (Appendix Fig S7B). Oligomycin and etomoxir pre-treatments had no effect on the phagocytosis of Kp52145, whereas inhibition of glycolysis using 2DG resulted in an increase of phagocytosis (Appendix Fig S7C). Time course experiments revealed that oligomycin and etomoxir pre-treatments did not impair Kp52145 intracellular survival (Fig 6I). In stark contrast, 2DG pre-treatment significantly reduced the survival of Kp52145 (Fig 6I). Moreover, 2DG pre-treatment increased the co-localisation of the KCV with cresyl violet, indicating that inhibition of glycolysis results in an increased fusion of the KCV with lysosomes (Fig 6J and K). Interestingly, oligomycin and etomoxir pre-treatments had no effect on the activation of NF-κB and Irf3 following infection whereas 2DG pre-treatment resulted in a significant decrease in NF-κB and Irf3 activation after Kp52145 infection (Appendix Fig S7D and E). The latter result is consistent with the importance of glycolysis to mount inflammatory responses following infection (Pålsson-McDermott & O'Neill, 2020).

Altogether, these experiments demonstrate that glycolysis is associated with *K. pneumoniae*-induced M(Kp) polarisation. Moreover, our results highlight the importance of host cell glycolysis for *K. pneumonaie* survival while OXPHOS seems dispensable.

Klebsiella pneumoniae-governed M(Kp) is dependent on the capsule polysaccharide

We next sought to identify the *K. pneumoniae* factor(s) governing M (Kp) polarisation. Given that TLR4 governs M(Kp) and that the *K. pneumoniae* capsule polysaccharide (CPS) and the LPS O-polysaccharide are recognised by TLR4 (Regueiro *et al*, 2009; Yang *et al*, 2011), we asked whether these polysaccharides mediate *K. pneumoniae* induction of the M(Kp) polarisation. The LPS-O-polysaccharide mutant, strain 52145-Δ*glf* (Sa-Pessoa *et al*, 2020), induced the phosphorylation of STAT6 (Fig 7A). The mutant also upregulated the levels of the M(Kp) marker CD206 to the same levels as Kp52145-infected macrophages (Fig 7B). In contrast, the *cps* mutant, strain 52145-Δwca_{K2} (Llobet *et al*, 2008), did not trigger the phosphorylation of STAT6 (Fig 7C), and did not induce *arg1* (Fig 7D). Single-cell experiments using flow cytometry demonstrated that the levels of Arg1, and CD206 were significantly lower in macrophages infected with the *cps* mutant than in those infected with the wild-type strain (Fig 7E and F). The opposite was found for the M1 marker MHC-II (Fig 7G). Complementation of the *cps* mutant restored the expression levels of M(Kp) markers to those induced by the wild-type strain (Fig 7D–G). Taken together, this evidence supports the notion that *K. pneumoniae* CPS controls M(Kp) polarisation.

©2022 The Authors

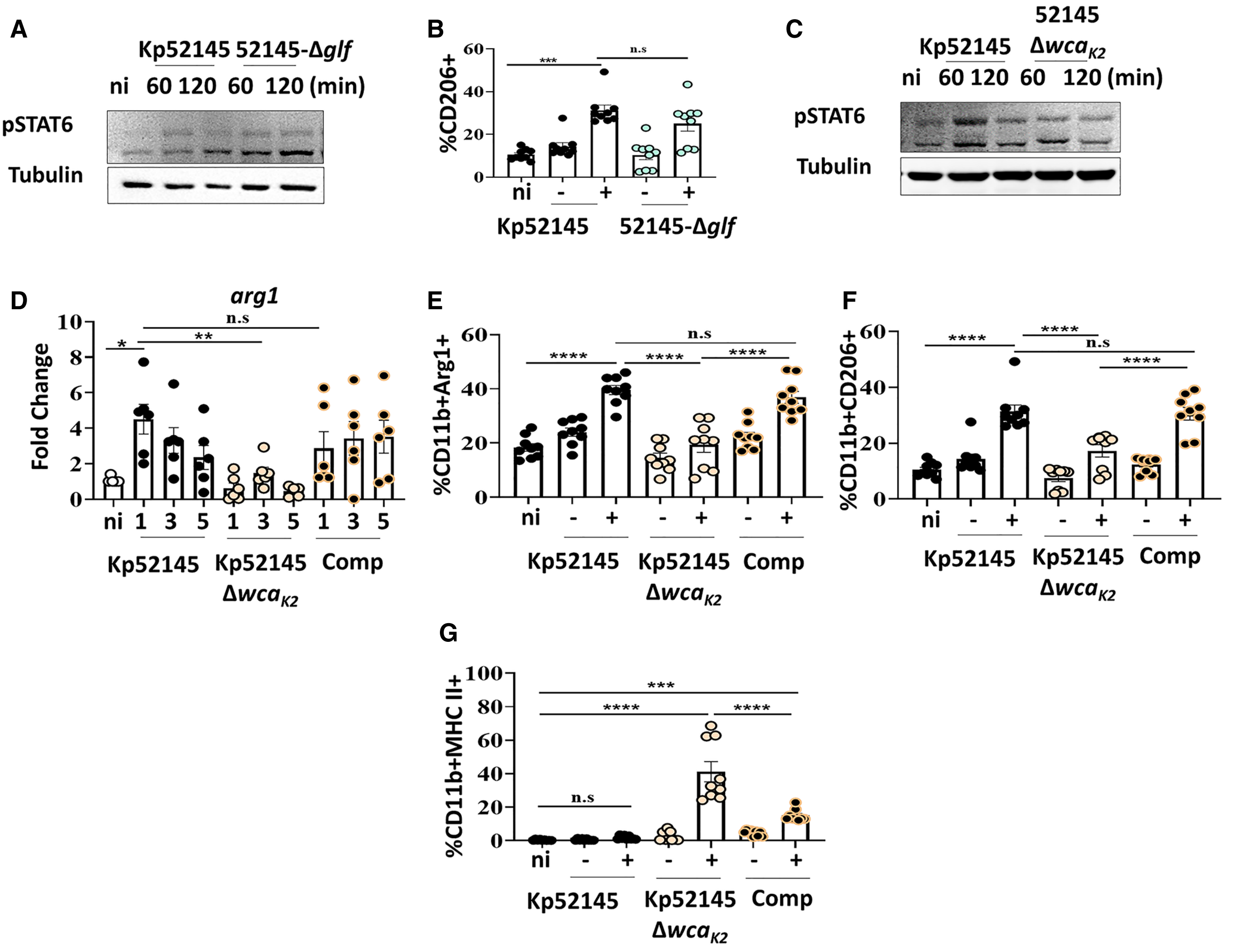

Figure 7. ***Klebsiella pneumoniae*-governed M(Kp) is dependent on the capsule polysaccharide.**

A Immunoblot analysis of phospho-STAT6 (pSTAT6) and tubulin levels in lysates from wild-type macrophages non-infected (ni), or infected with Kp52145 or the LPS O-polysaccharide mutant, strain 52145-Δ*glf*, for 60 or 120 min.

B Percentage of wild-type macrophages with and without associated Kp52145 or 52145-Δ*glf* positive for CD206 5 h post infection. Bacteria were tagged with mCherry.

C Immunoblot analysis of phospho-STAT6 (pSTAT6) and tubulin levels in lysates from wild-type macrophages non-infected (ni), or infected with Kp52145 or the CPS mutant, strain 52145-Δwca_{K2}, for 60 or 120 min.

D *arg1* mRNA levels were assessed by qPCR in wild-type macrophages non-infected (ni) or infected Kp52145, the CPS mutant, strain 52145-Δwca_{K2}, the complemented strain, 52145-Δwca_{K2}/pGEMTman (Comp) for 1, 3 or 5 h.

E Percentage of wild-type macrophages with and without associated Kp52145, 52145-Δwca_{K2} or the complemented strain positive for Arg1 5 h post infection. Bacteria were tagged with mCherry.

F Percentage of wild-type macrophages with and without associated Kp52145, 52145-Δwca_{K2} or the complemented strain positive for CD206 5 h post infection. Bacteria were tagged with mCherry.

G Percentage of wild-type macrophages with and without associated Kp52145, 52145-Δwca_{K2} or the complemented strain positive for MHCII 5 h post infection. Bacteria were tagged with mCherry.

Data information: For all infections, after 1 h contact, medium replaced with medium containing gentamycin (100 μg/ml) to kill extracellular bacteria. Error bars are presented as the mean ± SEM of three independent experiments in duplicate or triplicate. Images are representative of three independent experiments. Statistical analysis were carried out using one-way ANOVA with Bonferroni contrast for multiple comparisons test. ****$P \le 0.0001$; ***$P \le 0.001$; **$P \le 0.01$*$P \le 0.05$; ns, $P > 0.05$ for the indicated comparisons.

Klebsiella pneumoniae induces M(Kp) polarisation in human macrophages

We next focused to extend the work performed in mice to humans to determine whether *K. pneumoniae* also triggers M(Kp) polarisation in human macrophages. To address this question, we infected macrophages generated from human PBMCs from six different healthy donors. Violin plots show that Kp52145 infection increased the levels of the M(Kp) markers *arg1*, *il10*, *chi3l1*, *ido* (Fig 8A–D), *pparγ*, *mrc1*, *nos2*, *isg56* and *il1rn* (Fig EV5A–E). *il1rn*

 ©2022 The Authors

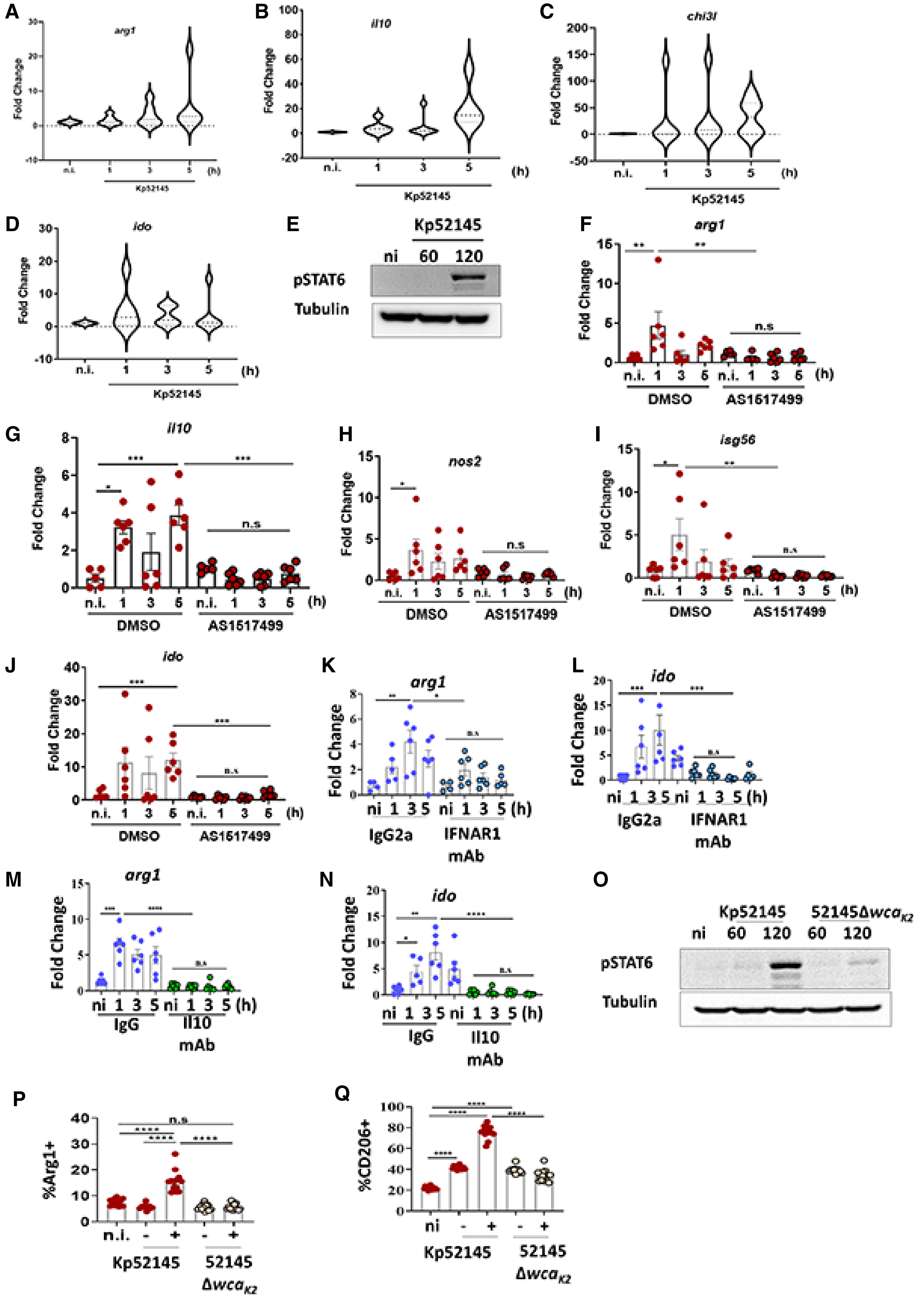
A
arg1
Fold Change
Kp52145
B
il10
Fold Change
n.i.
1
3
5
(h)
Kp52145
C
chi3l
Fold Change
Kp52145
D
ido
Fold Change
Kp52145
E
Kp52145
ni 60 120
pSTAT6
Tubulin
F
arg1
Fold Change
DMSO
AS1517499
G
il10
Fold Change
DMSO
AS1517499
H
nos2
Fold Change
DMSO
AS1517499
I
isg56
Fold Change
DMSO
AS1517499
J
ido
Fold Change
DMSO
AS1517499
K
arg1
Fold Change
IgG2a
IFNAR1 mAb
L
ido
Fold Change
IgG2a
IFNAR1 mAb
M
arg1
Fold Change
IgG
Il10 mAb
N
ido
Fold Change
IgG
Il10 mAb
O
Kp52145
52145Δwca$_{K2}$
ni 60 120 60 120
pSTAT6
Tubulin
P
%Arg1+
n.i.
Kp52145
52145 Δwca$_{K2}$
Q
%CD206+
ni
Kp52145
52145 Δwca$_{K2}$

Figure 8.

Figure 8. *Klebsiella pneumoniae* induces M(Kp) polarisation in human macrophages.

A *arg1* mRNA levels were assessed by qPCR in hM-CSF-treated PBMCs from 6 donors non-infected (ni) or infected Kp52145 for 1, 3 or 5 h.
B *il10* mRNA levels were assessed by qPCR in M-CSF-treated PBMCs from 6 donors non-infected (ni) or infected Kp52145 for 1, 3 or 5 h.
C *chi3l* mRNA levels were assessed by qPCR in hM-CSF-treated PBMCs from 6 donors non-infected (ni) or infected Kp52145 for 1, 3 or 5 h.
D *ido* mRNA levels were assessed by qPCR in hM-CSF-treated PBMCs from 6 donors non-infected (ni) or infected Kp52145 for 1, 3 or 5 h.
E Immunoblot analysis of phospho-STAT6 (pSTAT6) and tubulin levels in lysates from PMA-treated THP-1 macrophages non-infected (ni), or infected with Kp52145 for 60 or 120 min.
F *arg1* mRNA levels were assessed by qPCR in PMA-treated THP-1 macrophages non-infected (ni) or infected Kp52145 for 1, 3 or 5 h and treated with the STAT6 inhibitor AS1517499 or DMSO vehicle control.
G *il10* mRNA levels were assessed by qPCR in PMA-treated THP-1 macrophages non-infected (ni) or infected Kp52145 for 1, 3 or 5 h and treated with the STAT6 inhibitor AS1517499 or DMSO vehicle control.
H *nos2* mRNA levels were assessed by qPCR in PMA-treated THP-1 macrophages non-infected (ni) or infected Kp52145 for 1, 3 or 5 h and treated with the STAT6 inhibitor AS1517499 or DMSO vehicle control.
I *isg56* mRNA levels were assessed by qPCR in PMA-treated THP-1 macrophages non-infected (ni) or infected Kp52145 for 1, 3 or 5 h and treated with the STAT6 inhibitor AS1517499 or DMSO vehicle control.
J *ido* mRNA levels were assessed by qPCR in PMA-treated THP-1 macrophages non-infected (ni) or infected Kp52145 for 1, 3 or 5 h and treated with the STAT6 inhibitor AS1517499 or DMSO vehicle control.
K *arg1* mRNA levels were assessed by qPCR in PMA-treated THP-1 macrophages non-infected (ni) or infected Kp52145 for 1, 3 or 5 h and treated with IFNAR1 blocking antibody or isotype control.
L *ido* mRNA levels were assessed by qPCR in PMA-treated THP-1 macrophages non-infected (ni) or infected Kp52145 for 1, 3 or 5 h and treated with IFNAR1 blocking antibody or isotype control.
M *arg1* mRNA levels were assessed by qPCR in PMA-treated THP-1 macrophages non-infected (ni) or infected Kp52145 for 1, 3 or 5 h and treated with IL10 blocking antibody or isotype control.
N *ido* mRNA levels were assessed by qPCR in PMA-treated THP-1 macrophages non-infected (ni) or infected Kp52145 for 1, 3 or 5 h and treated with IL10 blocking antibody or isotype control.
O Immunoblot analysis of phospho-STAT6 (pSTAT6) and tubulin levels in lysates from PMA-treated THP-1 macrophages non-infected (ni), or infected with Kp52145 or the CPS mutant, strain 52145-Δwca_{K2}, for 60 or 120 min.
P Percentage of PMA-treated THP-1 macrophages with and without associated Kp52145 or 52145-Δwca_{K2} positive for Arg1 5 h post infection. Bacteria were tagged with mCherry.
Q Percentage of PMA-treated THP-1 macrophages with and without associated Kp52145 or 52145-Δwca_{K2} positive for CD206 5 h post infection. Bacteria were tagged with mCherry.

Data information: For all infections, after 1 h contact, medium replaced with medium containing gentamycin (100 μg/ml) to kill extracellular bacteria. Error bars are presented as the mean ± SEM of three independent experiments in duplicate. Images are representative of three independent experiments. Statistical analysis were carried out using one-way ANOVA with Bonferroni contrast for multiple comparisons test. ****$P \leq 0.0001$; ***$P \leq 0.001$; **$P \leq 0.01$; *$P \leq 0.05$; ns, $P > 0.05$ for the indicated comparisons.

and *ido* are two of the markers often associated with M2 polarisation in human macrophages (Martinez *et al*, 2006). These results suggest that *K. pneumonaie* also skews macrophage polarisation in human macrophages towards M(Kp).

To ascertain whether *K. pneumoniae* exploits the same molecular mechanisms in human macrophages than in mouse macrophages to induce M(Kp) polarisation, we switched to PMA-differentiated THP-1 human macrophages. This cell line derived from a patient with acute monocytic leukaemia and it is used commonly to model the activation of human macrophages. Immunoblotting experiments confirmed that Kp52145 induced the phosphorylation of STAT6 (Fig 8E). RT-qPCR experiments showed that Kp52145 infection increased the levels of the M(Kp) markers *arg1*, *il10*, *nos2*, the ISG *isg56* and *ido* in THP-1 cells in a STAT6-dependent manner because the STAT6 inhibitor AS1517499 abrogated *Klebsiella*-mediated upregulation of these M(Kp) markers (Fig 8F–J). Collectively, these results demonstrate that *K. pneumoniae* triggers M(Kp) polarisation in THP-1 cells in a STAT6-dependent manner.

To determine whether *K. pneumoniae*-induced type I IFN and IL10 would also govern the induction of M(Kp) polarisation in human macrophages, cells were infected in the presence of blocking antibodies against human IFNAR1 receptor, and IL10. Fig 8K and L show that Kp52145 did not upregulate the expression *of arg1* and *ido* when infections were done in the presence of IFNAR1 blocking antibody. Similar results were obtained following IL10 suppression (Fig 8M and N). Together, these results demonstrate that type I IFN and IL10 signalling are crucial for *K. pneumoniae* induction of M(Kp) polarisation in human macrophages.

To establish whether *K. pneumoniae* CPS also controls M(Kp) polarisation in human macrophages, PMA-differentiated THP-1 cells were infected with the *cps* mutant, strain Kp52145 Δwca_{K2}. Immunoblotting experiments showed that the *cps* mutant did not induce the phosphorylation of STAT6 in THP-1 cells (Fig 8O). As anticipated, single-cell analysis by flow cytometry revealed that infection with the *cps* mutant did not increase the levels of Arg1 (Fig 8P) and CD206 (Fig 8Q). Taken together, these results demonstrate that *K. pneumoniae* CPS also governs M(Kp) polarisation in human macrophages.

Discussion

Klebsiella pneumoniae is one of the pathogens sweeping the World in the antibiotic- resistant pandemic. Although, there is a wealth of knowledge on how *K. pneumoniae* develops resistance to different antibiotics, we still lack a complete understanding of what makes *K. pneumoniae* a successful pathogen. Of particular interest is to uncover whether *K. pneumoniae* has evolved strategies to overcome macrophages. These cells are an integral component of the tissue immune surveillance, response to infection and the resolution of inflammation. Therefore, pathogens such as *Klebsiella* cannot avoid innate immune responses if they are not able to overcome

 ©2022 The Authors

macrophages. In this work, we demonstrate that *K. pneumoniae* induces a singular polarisation state, termed M(Kp), in distinct populations of macrophages, AMs and IMs. Our findings demonstrate the central role of STAT6 in *K. pneumoniae*-governed M(Kp). Mechanistic studies revealed that *K. pneumoniae* hijacks the immune effectors type I IFN and IL10 following the activation of TLR-controlled pathways to activate STAT6-controlled M(Kp). These results illustrate how the co-evolution of *K. pneumoniae* with the immune system has resulted in the pathogen exploiting immune effectors and receptors sensing infections to thwart innate immune defences. We establish that STAT6 is necessary for *K. pneumoniae* intracellular survival, whereas absence of STAT6 *in vivo* facilitates the clearance of the pathogen, revealing that STAT6-governed macrophage polarisation plays an integral role in *K. pneumoniae* infection biology.

Our knowledge of the interface between macrophages and bacterial pathogens still relies on interrogating cellular models *in vitro*. Therefore, we have a poor understanding of the *in vivo* molecular dynamics of the interface between pathogens and the populations of tissue-resident macrophages. Our results establish that lung IMs are the main target of *K. pneumoniae* although we cannot delineate whether there are differences on the interaction of *Klebsiella* with any of the subsets of known IMs. IMs show marked upregulation of gene sets related to reactive oxygen species (ROS) biosynthesis, high levels of nitric oxide, iron sequestration and inflammatory responses (Gibbings *et al*, 2017; Chakarov *et al*, 2019). Therefore, IMs are considered to show increased microbicidal activity. Consistent with this role, infection triggers the recruitment of IMs (this work and Xiong *et al*, 2015; Xiong *et al*, 2016) and, for example, they control *Mycobacterium tuberculosis in vivo* (Huang *et al*, 2018). In contrast, our clodronate-loaded liposomes-based experiments suggest that IMs are essential for *Klebsiella* survival *in vivo*. This is consistent with the upregulation of networks related to M(Kp), an M2-like anti-inflammatory polarisation state that it cannot be ascribed to any of the M2 subtypes (Murray *et al*, 2014). The markers characteristic of *K. pneumoniae*-controlled macrophage polarisation were Arg1, Fizz1, iNOS, CD163, *cd206*, type I IFN and IL10 signalling-regulated genes, and the decreased expression of *ppar*γ, and inflammatory markers. On the other hand, depletion of AMs resulted in a marked increase of *K. pneumoniae* survival, indicating that these macrophages play a pivotal role controlling *K. pneumoniae* infection as it has been postulated (Broug-Holub *et al*, 1997; Xiong *et al*, 2015). It is worth noting that in our analysis we cannot differentiate between alveolar macrophages and monocyte-derived alveolar macrophages although the latter are recruited to the lungs within 3–7 days (Misharin *et al*, 2017; Aegerter *et al*, 2020; Arafa *et al*, 2022) and our experiments were done 24 h post infection. The role of AMs in defence against *Klebsiella* is consistent with the scRNA-seq analysis revealing an enrichment in gene networks related to TLR and NLR signalling, and inflammation in infected AMs. The fact that similar networks were found in AMs infected with *M. tuberculosis* (Pisu *et al*, 2021) suggests that this transcription programme is part of a common AM response to bacterial pathogens.

Our experiments illustrating the permissive (IMs) and restrictive (AMs) macrophage populations to *K. pneumoniae in vivo* relied on reducing their numbers by clodronate-loaded liposomes. This experimental strategy is widely used to investigate macrophages in tissues (van Rooijen & Hendrikx, 2010), and has also been used to investigate the role of macrophage populations in *M. tuberculosis* infection (Huang *et al*, 2018). AMs and the precursors of IMs, blood monocytes, were reduced by intranasal administration and intravenous injection, respectively. Although we are confident that our findings are directly dependent on these macrophage populations, we cannot rule out that other cell types such as dendritic cells might also contribute to the observed phenotype even if they represent a minor proportion of the lung immune cells.

To the best of our knowledge, *K. pneumoniae* is the first pathogen skewing the polarisation of lung macrophages. Our results are consistent with a scenario in which rewiring macrophage polarisation is a *K. pneumoniae* virulence strategy to survive in the lungs. Interestingly, *K. pneumoniae*-induced macrophage polarisation is species independent because similar findings were obtained in mouse, human and porcine macrophages (this work and (Dumigan *et al*, 2019)). The fact that there are significant differences between macrophages from different species (Fairbairn *et al*, 2011) suggests that *K. pneumoniae* cellular targets should be conserved throughout evolution. Indeed, our results demonstrate that *K. pneumoniae* targets STAT6 across species to induce M(Kp) polarisation (this work and (Dumigan *et al*, 2019)). This transcriptional factor arose early during the evolution of vertebrates (Wang & Levy, 2012) and belongs to the STAT family which is conserved through evolution from the first single-cell organisms (Wang & Levy, 2012). Remarkably, and despite the role of STAT6 governing macrophage polarisation, this transcriptional factor is not a common target of pathogens to control macrophage biology, being *Klebsiella* the first bacterial pathogen doing so.

It was intriguing to determine how *K. pneumoniae* manipulates the polarisation of macrophages because it does not encode the known secretion systems or toxins affecting cell biology. Our work demonstrates that *K. pneumoniae*-controlled STAT6 activation was dependent on type I IFN and IL10 following the activation of TLR4-governed signalling pathway. Type I IFNs, IL10 and TLR4 also appeared early during the evolution of vertebrates (Piazzon *et al*, 2016; Liu *et al*, 2020). Therefore, *K. pneumoniae* has evolved to manipulate an innate axis conserved during evolution formed by TLR4-type I IFN-IL10-STAT6 to rewire macrophages. This is a previously unknown axis exploited by a human pathogen to manipulate immunity. This underappreciated anti-immune strategy is radically different from that employed by other pathogens, such as *Listeria*, *Salmonella*, *Shigella* or *Mycobacterium*, which deliver bacterial proteins into host cells to manipulate the host. However, we believe that indeed it is an emerging theme in the infection biology of bacterial pathogens. Providing further support to this notion, TLR-controlled signalling is exploited by *S. Typhimurium* to survive in macrophages (Arpaia *et al*, 2011), and *L. monocytogenes* leverages type I IFN for intracellular survival (Frantz *et al*, 2019; Pagliuso *et al*, 2019).

Our work sheds new light into the role of IL10 on *K. pneumoniae* infection biology. The importance of IL10 *in vivo* is marked by the fact that neutralisation of the cytokine enhances the clearance of the *K. pneumoniae* (Greenberger *et al*, 1995). However, it remained an open question the exact role of IL10 in *K. pneumoniae*–host interaction beyond the well-known role of IL10 to downregulate inflammation. In this work, we establish that IL10 is essential to skew macrophage polarisation and, in fact, IL10 is one of the signatures of M(Kp). In addition, our results demonstrate that IL10 is essential

for the intracellular survival of *Klebsiella*. How *K. pneumoniae* induces IL10 was unknown. Our results demonstrate that *K. pneumoniae*-induced IL10 is controlled by TLR4-MyD88-CREB signalling pathway. This is a well-established pathway governing the expression of IL10 (Saraiva & O'Garra, 2010). The facts that CREB affects the activation of host defence responses independently of IL10, and that CREB regulates T cells (Wen *et al*, 2010) suggest that *K. pneumoniae* may leverage the immunomodulatory roles of CREB beyond IL10 production. Current efforts of the laboratory are devoted to investigate the role of CREB during *K. pneumoniae* infection.

Another novel finding of our work is the importance of glycolysis in *Klebsiella*–macrophage interface. Although reports indicate that OXPHOS is the metabolic signature of M2 macrophages (Biswas & Mantovani, 2012), our results demonstrate that glycolysis characterises M(Kp). This finding is not totally unprecedented and, for example, the M2 tumour-associated macrophages are metabolically distinct from conventional M2 polarised subset in prioritising usage of glycolysis as key metabolic pathway (Puthenveetil & Dubey, 2020). Notably, glycolysis is required for optimal survival of *K. pneumoniae* in macrophages. At present, we can only speculate why glycolysis benefits *K. pneumoniae* survival. It is possible that glycolysis yields metabolites that *Klebsiella* needs as nutrients when residing in the KCV. These metabolites may also result in the regulation of the virulence factors governing the intracellular lifestyle of *Klebsiella*. Supporting this possibility, inhibition of glycolysis resulted in an increased co-localisation of the KCV with lysosomes. Future studies are warranted to ascertain the effect of glycolysis and glycolysis-derived metabolites on *K. pneumonaie* virulence. Intriguingly, recent evidence suggest that this could be a general phenotype as Rosenberg *et al* (2021) have shown that the glycolysis metabolite succinate activates *Salmonella* virulence during intracellular infection (Rosenberg *et al*, 2021).

We were keen to identify the *K. pneumoniae* factor(s) governing the STAT6-mediated M(Kp) polarisation. We first focused on the CPS and the LPS- O-polysaccharide because both polysaccharides are sensed by TLR4 that governs *Klebsiella*-induced activation of STAT6. Our results establish that only the CPS induced the activation of STAT6-controlled M(Kp). Importantly, the CPS is essential for *K. pneumoniae* survival in mice (pneumonia model; Cortes *et al*, 2002), underlining the importance of M(Kp) induction as a *K. pneumoniae* virulence trait since this process is abrogated in this mutant strain. Previous studies from the laboratory and others demonstrate the role of the CPS limiting the engulfment by phagocytes (Regueiro *et al*, 2006; Evrard *et al*, 2010; Pan *et al*, 2011; March *et al*, 2013), illustrating the contribution of the CPS to *K. pneumoniae* stealth behaviour (Bengoechea & Sa Pessoa, 2019). However, the results of this work and our study demonstrating that the CPS activates an EGF receptor pathway to blunt inflammatory responses in epithelial cells (Frank *et al*, 2013) support that *K. pneumoniae* CPS is also one of the virulence factors of *K. pneumoniae* devoted to manipulate cell signalling.

Klebsiella pneumoniae exemplifies the global threat posed by antibiotic-resistant bacteria. *K. pneumoniae*-triggered pulmonary infection has a high mortality rate reaching 50% even with antimicrobial therapy and may approach 100% for patients with alcoholism and diabetes. Our findings explain why the accumulation of M2-like macrophages due to alcoholism or trauma favours *K. pneumoniae* infections as these factors will facilitate the rewire of macrophages by the pathogen. Nonetheless, our results probing the pneumonia mouse pre-clinical translational model sustain that *K. pneumoniae* will rewire macrophages even in the case of immunocompetent subjects, enabling infection. Absence of STAT6 resulted in macrophages consistent with M1 polarisation with increased ability to clear intracellular *Klebsiella*. *In vivo* experiments probing a pre-clinical pneumonia mouse model demonstrated increased clearance of *K. pneumoniae* following the inhibition of STAT6. Altogether, these results strongly support that STAT6 is a target to boost human defence mechanisms against *K. pneumoniae*. Host-directed therapeutics aiming to interfere with host factors required by pathogens to counter the immune system are emerging as untapped opportunities that are urgently needed in the face of the global pandemic of antibiotic-resistant infections. There is research to develop STAT6 inhibitors due to the implication of STAT6 signalling in colorectal cancer, melanoma and allergic lung diseases. Based on our novel results, we propose that these drugs shall show a beneficial effect to treat *K. pneumoniae* infections alone or as a synergistic add-on to antibiotic treatment. Future studies shall confirm whether this is the case.

Materials and Methods

Study approval

All animal procedures were performed in compliance with the UK Home Office and approved by the Queen's University Belfast Animal Welfare and Ethical Review Body (AWERB). The work described in this article was carried out under project licences PPL2778 and PPL2910.

Ethical approval for the use of blood from healthy volunteers was approved by the Research Ethics Committee of the Faculty of Medicine, Health and Life Sciences of Queen's University Belfast (approval reference MHLS 20_136). Whole blood was obtained from the Northern Ireland Blood Transfusion Service. Informed consent was obtained from all blood donors, and the experiments conformed to the principles set out in the WMA Declaration of Helsinki and the Department of Health and Human Services Belmont Report.

Animals and infection model

C57BL/6 mice were purchased from Charles River Laboratories. *Ifnar1*$^{-/-}$ and *stat6*$^{-/-}$ animals were used to generate iBMDM cell lines in this study. *Ifnar1*$^{-/-}$ mice are maintained in Queen's University Belfast animal facility, whereas *stat6*$^{-/-}$ animals were purchased from The Jackson Laboratory (Stock reference 005977). Mice were housed under standard laboratory conditions (12/12 h light/dark cycle with a room temperature of 21°C and water and food available *ad libitum*). Mice used for experiments were aged between 8 and 12 weeks old and sex matched. Animals were randomised between groups, but due to ethical regulations the researchers were not blinded to the treatments administered. No samples or animals were excluded from the analysis.

For *in vivo* infections, bacteria in the stationary phase, were subcultured and grown at 37°C with agitation to reach mid log phase. Subsequently, bacteria were harvested by centrifugation (20 min, 2,500 *g*, 24°C), resuspended in PBS and adjusted to 5×10^4–1×10^5

 ©2022 The Authors

colony-forming units (CFUs) per 30 μl as determined by plating serial dilutions on LB plates. Mice were anaesthetised with isoflurane, and infected intranasally with *K. pneumoniae* in 30 μl of PBS or mock-infected with PBS. In the experiments probing the STAT6 inhibitor AS1517499, mice were treated 24 h prior to infection intraperitoneally with 10 mg/kg AS1517499 (AXON) or DMSO vehicle control in 200 μl volume. Six hours post-infection, mice received intranasally an additional dose of AS1517499 or DMSO (5 mg/kg in 30 μl volume). After 24 h post infection, mice were euthanized and lungs, and spleen isolated for assessment of bacterial load (CFU), or lungs processed for flow cytometry or RNA. CFUs were determined by homogenising organs in 1 ml of sterile PBS and plating serial dilutions on *Salmonella-Shigella* agar plates (Sigma-Aldrich). Plates were incubated overnight at 37°C before counting of colonies.

Generation of *stat6*$^{-/-}$ and *ifnar1*$^{-/-}$ iBMDMs

Tibias and femurs were obtained from *ifnar1*$^{-/-}$ and *stat6*$^{-/-}$ mice (C57BL/6 background) and the bone marrow extracted under sterile conditions flushing bones with complete medium (DMEM, GlutaMAX, supplemented with 10% heat-inactivated foetal calf serum (FCS) and 1% penicillin–streptomycin). Red blood cells were lysed via incubation with ammonium-chloride-potassium (ACK) lysis buffer (A1049201; Gibco) for 5 min at room temperature. Cells were then washed in 10 ml of complete medium and passed through a 70-mm cell strainer (2236348; Fisherbrand) prior to centrifugation. Cell pellet was dislodged before plating on 20-cm petri dishes (Sarstedt) in complete medium supplemented with 20% syringe-filtered L929 supernatant, as source of macrophage colony-stimulating factor, and maintained at 37°C in a humidified atmosphere of 5% CO2. Medium was replaced with fresh medium supplemented with L929 after 1 day of culture. After 5 days, BMDMs were immortalised by exposing them to the J2 CRE virus (carrying v-myc and v-Raf/v-Mil oncogenes, kindly donated by Avinash R. Shenoy, Imperial College London) for 24 h. This step was repeated 2 days later (day 7), followed by continuous culture in DMEM supplemented with 20% (vol/vol) filtered L929 cell supernatant for 4 to 6 weeks. The presence of a homogeneous population of macrophages was assessed by flow cytometry using antibodies for CD11b (1:200, clone M1/70; catalogue number 17-0112-82; eBioscience) and CD11c (1:200, clone N418; catalogue number 48-0114-82; eBioscience).

Culture of iBMDMs

Wild-type, *Tlr2*$^{-/-}$. *Tlr4*$^{-/-}$, *Tlr2/4*$^{-/-}$, *Myd88*$^{-/-}$, *Tram/Trif*$^{-/-}$ immortalised bone marrow-derived macrophages (iBMDMs) were obtained from BEI Resources (NIAID, NIH; repository numbers NR-9456, NR-9457. NR-9458, NR-19975, NR-15633 and NR-9568, respectively). *Il10*$^{-/-}$ iBMDMs were described previously (Bartholomew *et al*, 2019). iBMDMs were maintained in DMEN (Gibco 41965) supplemented with 10% heat-inactivated FCS, 100 U/ml of penicillin and 0.1 mg/ml of streptomycin (Sigma-Aldrich) at 37°C in a humidified 5% CO_2 incubator. Cells were routinely tested for *Mycoplasma* contamination.

Human PBMCs

PBMCs were isolated using density gradient media Ficoll-Paque PLUS (Cytiva) after centrifugation at 790 *g* for 30 min. Resulting buffy coats were extracted and treated with ACK Lysis buffer (Gibco) to remove red blood cells, prior to freezing cells at a density of 1×10^6 per cryovial and stored at −80°C. Cells were broken out via rapid thawing in 37°C water bath before removal of DMSO via suspension in complete medium and centrifugation. Cells were then plated at a density of 3×10^5/well in 12-well tissue culture plates (Sarstedt) in DMEM (Gibco 41965) supplemented with 10% FCS, 100 U/ml of penicillin and 0.1 mg/ml of streptomycin (Sigma−Aldrich) supplemented with 10 ng/ml of human M-CSF (Cat: 75057, Stemcell) at 37°C in a humidified 5% CO_2 incubator for 5 days to allow differentiation of macrophages.

Culture and differentiation of THP-1 cells

Human THP-1 monocytes (ATCC TIB-202) were maintained in complete medium (RPMI, supplemented with 10% FCS and 5% penicillin/streptomycin) before seeding for differentiation in the presence of PMA (5 ng/ml) for 48 h prior to infection at a density of 3×10^5 cells/well in 24- well plates (Sarstedt). Cells were routinely tested for *Mycoplasma* contamination.

Bacterial strains and culture conditions

Kp52145 is a clinical isolate (serotype O1:K2) previously described (Nassif *et al*, 1989; Lery *et al*, 2014). Strains NJST258-1, NJST258-2, KP35 and SGH10 are also clinical isolates previously characterised (Deleo *et al*, 2014; Ahn *et al*, 2016; Lam *et al*, 2018). 52145-Δwca_{K2} is an isogenic mutant of Kp52145 lacking the capsule polysaccharide (CPS), which has been previously described (Llobet *et al*, 2008). 52145-Δ*glf* lacks the LPS O-polysaccharide and expresses similar levels of CPS than Kp52145 (Sa-Pessoa *et al*, 2020). mCherry expressing strains were generated by electroporation of pUC18T-mini-Tn7T-Apr-mCherry plasmid (Ducas-Mowchun *et al*, 2019) to Kp52145, 52145-Δwca_{K2} and 52145-Δ*glf*. To construct a multidrug-resistant KP35 strain expressing mCherry, mCherry gene and its promoter region were excised from plasmid pUC18T-mini-Tn7T-Apr-mCherry (Ducas-Mowchun *et al*, 2019) by HindII-KpnI digestion and cloned into HindIII-KpnI digested pUC18T-mini-Tn7T-Zeo (Ducas-Mowchun *et al*, 2019) to obtain pUC18T-mini-Tn7T-Zeo-mCherry. This plasmid was electroporated into KP35. mCherry expression in the *Klebsiella* strains was confirmed by confocal microscopy and flow cytometry. pFPV25.1Cm plasmid (March *et al*, 2013) was conjugated to Kp52145 to generate bacteria expressing GFP constitutively. Strains were grown in LB medium at 37°C on an orbital shaker (180 rpm). When appropriate, the following antibiotics were added to the growth medium at the indicated concentrations: carbenicillin, 100 μg/ml; chloramphenicol, 25 μg/ml; zeocin 1,000 μg/ml.

Growth curve analysis

For growth kinetics analysis, 5 μl of overnight cultures were diluted in 250 μl of LB containing DMSO vehicle control or metabolic inhibitors, and incubated at 37°C with continuous, normal shaking in a Bioscreen Automated Microbial Growth Analyser (MTX Lab Systems, Vienna, VA, USA). Optical density (OD; 600 nm) was measured and recorded every 20 min.

Complementation of the capsule mutant

The *manC* gene was amplified from Kp52145 genomic DNA using primers Kp52_manCcomp_Fwd1_NdeI (5′-CAT ATG ATG TTG CTT CCT GTG ATC ATG GC-3′) and Kp52_manCcomp_Rvs1_EcoRI (5′-GAA TTC TTA GCA ACG ACC ATA CTG GTC-3′) and Taq polymerase (Promega). The PCR fragment was gel purified and cloned into pGEM-T Easy (Promega) plasmid to obtain pGEMTmanC. This plasmid was electroporated into 52145-Δwca_{K2}, and transformants were selected by plating on LB agar containing carbenicillin (100 μg/ml). mCherry expressing strain was generated by electroporation of pJT04Cherry plasmid (Cano *et al*, 2015) and selection on plates containing carbenicillin (50 μg/ml), and tetracycline (6.25 μg/ml).

Macrophage infections

iBMDMs were seeded into 24-well plates (1.6 × 10^5 cells/well) for microscopy, 12-well dishes (5 × 10^5 cells/well) for immunoblotting, and for assessing intracellular survival, and 6-wells (1 × 10^6 cells/well) for RNA and flow cytometry in complete media and allowed to adhere overnight. THP-1 cells were differentiated with PMA in 12-well dishes (3 × 10^5 cells/well). On the day of infection, cells were washed with 1 ml of PBS, and 1 ml of antibiotic-free media was added to the wells. To prepare the inoculum for infections, bacteria were grown until mid-exponential phase in 5 ml LB medium, supplemented with the appropriate antibiotics when required, at 37°C on an orbital shaker (180 rpm). Bacteria were recovered by centrifugation (3,220 *g*, 20 min, 22°C), washed once with PBS and diluted in PBS to an OD_{600} of 1.0, which corresponds to approximately 5 × 10^8 CFU/ml. A multiplicity of infection of 70 bacteria per cell was used in 1 ml of appropriate medium without antibiotics. To synchronise infection, plates were centrifuged at 200 *g* for 5 min. After 1 h post infection, media was removed, cells washed with 1 ml 1 PBS and 1 ml of antibiotic-free media supplemented with 100 μg/ml of gentamicin (Sigma-Aldrich) were added to the wells. At the indicated time points, supernatants were removed and cells processed for immunoblotting, RNA extraction or flow cytometry.

Blocking antibodies, cytokines stimulations and treatment with inhibitors

For cytokine stimulation experiments, cells were treated with recombinant mouse IFNβ (1,000 U/ml, Stratech) or IL-10 (250 ng/ml, catalogue 210–10 Peprotech) 3 h prior to collection for immunoblotting.

To inhibit STAT6, cells incubated with the chemical STAT6 inhibitor AS 1517499 (50 μg/ml, 919486-40-1, AXON) or DMSO as vehicle control for 2 h prior to infection and maintained throughout.

To inhibit cellular metabolism, macrophages were treated with DMSO vehicle control, or cells were treated with DMSO vehicle, or 2-deoxyglucose (2DG, 3 μM), oligomycin (3 μM), etomoxir (50 μM) 2 h before infection and maintained thought. All of them were purchased from Sigma-Aldrich.

For IL-10 neutralisation experiments, PMA differentiated THP-1 cells were incubated 2 h prior to infection and maintained throughout with either, 1 μg/ml of mouse monoclonal anti-human IL-10 antibody (R&D Systems. Ref: AF-217-NA), or equivalent concentrations of human IgG (Invitrogen) as control. To target IFNAR1/2, PMA differentiated THP-1 cells were treated with 10 μg/ml of mouse monoclonal anti-human IFNAR2 (Thermo Fisher Scientific, MMHAR-2), or human IgG (Invitrogen) as control. After 1 h of contact with bacteria, cells were washed once with PBS and complete medium without antibiotics supplemented with 100 μg/ml of gentamicin (Sigma−Aldrich) and with the relevant antibodies.

RNA extraction, and RT-qPCR

Lung tissue was homogenised using a VDI 12 tissue homogeniser (VWR) in 1 ml of TRizol reagent (Ambion) and incubated at room temperature for 5 min before storage at −80°C. Total RNA was extracted according to manufacturer's instructions. Five micrograms of total RNA were treated with recombinant DNase I (Roche Diagnostics Ltd) at 37°C for 30 min and then purified using a standard phenol–chloroform method. The RNA was precipitated with 20 μl of 3 M sodium acetate (pH 5.2) and 600 μl of 98% (v/v) ethanol at −20°C, washed twice in 75% (v/v) ethanol, dried and then resuspended in RNase-free H_2O.

To purify RNA from cells, they were washed once with 1 ml of PBS before lysis in TRizol reagent (Ambion) according to the manufacturer's instructions.

Duplicate cDNA preparations from each sample were generated from 1 μg of RNA using Moloney murine leukaemia virus (M-MLV) reverse transcriptase (Sigma−Aldrich) according to the manufacturer's instructions. qPCR analysis of gene expression was undertaken using the KAPA SYBR FAST qPCR kit and the Rotor-Gene Q5Plex qPCR system (Qiagen). Thermal cycling conditions were as follows: 95°C for 3 min for enzyme activation, 40 cycles of denaturation at 95°C for 10 s and annealing at 60°C for 20 s. Primers used in qPCRs are listed in Table EV1. cDNA samples were tested in duplicates, and relative mRNA quantity was determined by the comparative threshold cycle (ΔΔCT) method, using hypoxanthine phosphoribosyl transferase 1 gene normalisation for mouse and human samples.

Flow cytometry

Lung tissue was homogenised using a VDI 12 tissue homogeniser (VWR) in 1 m of sterile PBS and filtered through a 70-μm cell strainer (2236348 Fisherbrand) to generate single-cell suspension. Suspensions were centrifuged and red cells lysed using ACK lysis buffer (A1049201, Gibco), and washed once with 1 ml of PBS. Cell lines were washed once with 1 ml of ice cold PBS 5 h post infection and dislodged by scraping in a further 1 m of PBS. Murine samples were treated with Fc block (1:1000, clone 93, Ref: 101302, BioLegend) at 4°C for 15 min at 4°C. ~ 5 × 10^5 cells per tube were washed prior to incubation with combinations of the following rat anti-mouse antibodies against cell surface markers: Ly6G (1:800, Ref. 127616. BioLegend), Ly6C APC/Cy7 (1:600, clone HK1.4, Ref: 128026), CD11b APC (1:200, Ref: 101212), CD11c Pacific Blue (1:200, Ref: 117322), SiglecF FITC (1:200, Ref: 155504), BioLegend or SiglecF APCCy7 (1:200, Ref: 565527, Thermo Fisher), CD163 purified antibody (Ref: 155302) conjugated with FITC conjugation kit (Ref: ab102884, Abcam), CD206 FITC (Ref: 141704) according to manufacturer's instructions. Cells were incubated with antibodies

 ©2022 The Authors

for 15 min at 4°C prior to washing with 1 ml per tube FACS buffer (PBS with 2% FCS) prior to analysis by flow cytometry.

For experiments including the intracellular targets, this protocol was extended to include 10 min incubation at room temperature in 100 μl of fixative (eBiosciences FOXp3/Transcription factor staining buffer set, ref: 00-5523-00) followed by wash in 1 ml permeabilisation buffer (eBiosciences FOXp3/Transcription factor staining buffer set, ref: 00-5523-00) before incubation overnight with Arg1 FITC (1:200, Ref: 53-3697-82, Thermo Fisher), and iNOS PE (Ref: 12-3920-82, Thermo Fisher) antibodies in 100 μl of permeabilisation buffer at 4°C. Cells were then washed once more in 1 ml of permeabilisation buffer before flow cytometric analysis using the Canto II (BD).

THP-1 human macrophages were treated with mouse anti-human Fc block (1:1,000, Ref: 422302, BioLegend), for 15 min at 4°C prior to wash in 1 ml of PBS and centrifugation at 1,600 rpm for 5 min and surface molecule staining with CD206 APCCy7 (1:200, BioLegend), and intracellular staining of anti-human Arginase1 FITC (1:200, Ref: 53-3697-82, Thermo Fisher). Cell surface markers were stained directly while intracellular staining required additional processing using a cell fixation and permeabilisation kit as described above (eBiosciences FOXp3/Transcription factor staining buffer set, ref: 00-5523-00). Cell suspensions were analysed using CANTO-II analyser (BD). FlowJo V10 (Tree Star) software was used for data analysis and graphical representation.

Clodronate liposome-based reduction of alveolar and interstitial macrophages *in vivo*

To deplete resident alveolar macrophages in the lung, C56BL/6 mice received 50 μl of PBS or 50 μl of Clodronate-containing liposomes (stock solution 5 mg/ml) via intranasal application 24 and 4 h prior to infection. To deplete peripheral macrophages recruited to lung, wild-type mice received 200 μl of PBS or 200 μl of Clodronate-containing liposomes (stock solution 5 mg/ml) via retro orbital injection 24 and 4 h prior to infection. Twenty-four hours post-infection, bacterial burden was determined by serial dilutions of lung homogenates on *Salmonella-Shigella* agar. Lung homogenates were also processed for flow cytometry as previously described to assess the populations of neutrophils, interstitial macrophages and alveolar macrophages.

Cell sorting and scRNA-seq

C57BL/6 age and sex-matched animals (15 per group) were infected intranasally under isoflurane anaesthesia with mCherry expressing Kp52145 or PBS. After 24 h, lungs were homogenised, pooled and red cells lysed using ACK buffer (Gibco) for 5 min at room temperature. Cells were then washed in 10 ml of PBS before centrifugation at 1,600 rpm for 5 min. Supernatants were aspirated and cells were then treated with Fc block (1:1,000, clone 93, Ref: 101302, BioLegend,) at 4°C for 15 min. Cells were washed again prior to incubation with combinations of the following rat anti-mouse antibodies: against cell surface markers Ly6C APC/Cy7 (clone HK1.4, Ref: 128026, 1:600 dilution), CD11b APC (Ref: 101212, 1:200 dilution), CD11c Pacific Blue (Ref: 117322, 1:200 dilution), SiglecF FITC (Ref: 155504, BioLegend, 1:200 dilution). Using the FACS Aria II (BD Biosciences) $Ly6C^{+}CD11b^{+}CD11c^{+}SiglecF^{-}$ IMs and $Ly6C^{+}CD11b^{-}CD11c^{+}$Siglec F^{+} AMs were sorted from PBS control mice. From infected animals, four separate populations were retrieved, namely IMs infected with Kp52145 ($Ly6C^{+}CD11b^{+}CD11c^{+}SiglecF^{-}mCherry^{+}$), bystander IMs ($Ly6C^{+}CD11b^{+}CD11c^{+}SiglecF^{-}mCherry^{-}$), Kp52145-infected AMs ($Ly6C^{+}CD11b^{-}CD11c^{+}$ Siglec $F^{+}mCherry^{+}$) and bystander AMs ($Ly6C^{+}CD11b^{-}CD11c^{+}$ Siglec $F^{+/-}mCherry^{-}$) populations. Cells were collected into sterile 1× PBS and viability of all six populations was confirmed by trypan blue staining (Sigma-Aldrich) and found to be at or above 95% viable. Cells were sequenced using 10x Genomics by the Genomics Core Technology Unit, Queen's University Belfast.

scRNA-seq analysis

The data have been deposited to the NCBI Gene Expression Omnibus repository with the accession number GSE184290.

Cell Ranger (version 3.0.2) was used to process raw sequencing data. Using Mkfastq, Bcl files were converted to fastq format and demultiplexed into 6 libraries corresponding to the individual samples. Reads were quantitatively aligned to *Mus musculus* reference transcriptome (mm10) with Count. Cell Ranger was used to distinguish between data from viable cells and background signal, providing filtered gene-cell count matrices for downstream analysis.

QC and clustering: filtered count matrices were analysed in R (3.6.2) using Seurat 3.1.3 (Stuart *et al*, 2019). For each library, a further QC step was performed to remove genes expressed in < 3 cells, and cells with fewer than 200 genes or with > 25% counts mapping to mitochondrial genes. The libraries were then merged resulting in a dataset of 7,462 cells with 15,547 genes. Strong overlap of replicates within the alveolar and interstitial samples indicated the absence of batch effect, therefore library integration with batch correction was not required.

After log normalisation, SingleR 1.4.0 (Aran *et al*, 2019) was used to predict cell type in comparison to Immunological Genome Project (Heng *et al*, 2008) reference data, based on gene expression correlation. Predicted cell type was used to achieve *in silico* purification, keeping only those cells identified as macrophages/monocytes. After this step, the combined dataset contained 5,677 cells, with the libraries ranging between 422 and 1,927 cells. All other libraries were randomly down sampled to the lowest total (repeated ×3 for robustness), resulting in a final dataset of six libraries at 422 cells each, totalling 2,532 cells.

The resulting data were scaled to regress out cell variation attributed to mitochondrial and ribosomal gene expression and total counts per cell. The top 2,000 variable genes were identified and used as input for principal component analysis; 50 principal components (PCs) were tested in Seurat's JackStraw function, from which the first 38 PCs were identified as explaining more variability than expected by chance. These 38 PCs were used as input to SNN clustering (repeated at resolutions between 0.3 and 1.2) and UMAP generation.

Differential expression analysis: Marker gene detection and differential expression testing was performed in Seurat using the MAST package (version 1.12.0; Finak *et al*, 2015). Genes expressed in at least 10% of cells in either group being tested, with log fold-change > 0.25, and with adjusted *P*-value < 0.05 were considered significantly differentially expressed. Differentially expressed genes were displayed as volcano plots using EnhancedVolcano 1.4.0.

©2022 The Authors

Pseudotime analysis: Purified data were exported to Monocle 3.0 (Cao *et al*, 2019) for pseudotime analysis to model expression changes in pseudotime between control and infected cells. Inferred cell trajectories were calculated on UMAP embeddings as generated in Seurat, resulting in several distinct trajectory branches. Where possible, nodes relating to control cells were considered the 'start' of each trajectory, with each terminal node in the Bystander and Kp52145+ groups considered distinct endpoints. Each distinct trajectory was tested separately, with correlation between gene expression and pseudotime trajectory calculated using the Moran's I test in Monocle, filtered for significance on *P*- and *Q*-value < 0.05, and Moran's I test statistic > 0.2.

Pathway analysis and gene networks: Pathway enrichment analysis was performed using gProfiler. For each comparison, we created a list of genes as query, selecting only those genes where adjusted *P* value < 0.05. The analysis was performed using the g:SCS method for multiple testing correction, the Reactome database as a data source, and the default settings for the other parameters in g:Profiler. Results were exported to Cytoscape (version 3.8.2) and visualised using the AutoAnnotate application. The enrichment of transcriptional factors, their genomic binding sites and DNA binding profiles were analysed using TRANSFAC (20) included within g:Profiler. STRING database was used to predict protein–protein interactions using the clustering algorithm MCL with default parameters.

Seahorse analysis

iBMDMs were seeded in Seahorse XF Cell Culture Microplates at a density of 2×10^4 per well and allowed to adhere overnight in complete media at 37°C in a humidified 5% CO_2 incubator overnight. Seahorse cartridge was hydrated overnight in Seahorse XF Calibrant solution (Ref: 100840–000, Agilent) at 37°C overnight in a non-CO_2 incubator. iBMDMs were washed once with warmed PBS and maintained in 200 µl of complete media without antibiotics. When indicated, cells were treated with DMSO or STAT6 inhibitor AS1715499 (50 nM, Ref: 919486-40-1; AXON Medchem) 2 h prior to infection and maintained throughout. iBMDMs were infected at 70:1 multiplicity of infection. The infection was synchronised by centrifugation at 200 *g* for 5 min. After 1 h contact, cells were washed with PBS, and replenished with 180 µl of XF base medium (Ref 103334-100, Agilent) pH 7.4 supplemented with 1 mM of pyruvate, 2 mM of glutamine, 10 mM of glucose and 30 µg/ml of gentamicin to eliminate extracellular bacteria. Metabolic activity of cells was assessed using the Seahorse Mito Stress Test Kit (Ref: 103015–100, Agilent) according to manufacturer's instructions, and results were analysed with the Seahorse XFe96 analyser. Eight wells were used per condition in three independent experiments.

Immunoblotting

Macrophages were infected with *K. pneumoniae* strains for time points indicated in the figure legends. Cells were then washed in 1 ml of ice-cold PBS and lysed in 90 µl of Laemmli buffer (4% SDS, 10% 2-mercaptoehtanol, 20% glycerol, 0.004% bromophenol blue, 0.125 M Tris–HCl pH 6.8). The cell lysates were sonicated for 10 s at 10% amplitude (Branson Sonifier), boiled at 95°C for 5 min and centrifuged at 12,000 *g* for 1 min. Ten microlitres of the cell lysates were resolved by standard 10% SDS-PAGE gel and electroblotted onto 0.2 mm nitrocellulose membrane (Biotrace, VWR) using a semi-dry transfer unit (Bio-Rad). Membranes were blocked with 3% bovine serum albumin in Tris-buffered saline with Tween 20 (TBST).

Primary antibodies used were: p-Stat6 (Tyr641) (D8S9Y) (1:2,000, Ref: 56554), p-STAT1 (T701) (58D6) (1:2,000, Ref: 9167), p-STAT3 (Y705) (1:2,000, Ref: 9145), Total STAT3 (1:2,000, Ref: 12640), Arginase-1 (D4E3M) XP® (1:2,000, Ref: 93668), KLF4 (1:2,000, Ref: 4038) all from Cell Signalling Technologies. FIZZ-1/RELM (1:1,000, Ref: AF1523, R&D Systems), pCREB (1:1,000, Ref: sc-7978-R, Santa Cruz), Tubulin (1:4,000, Ref: T6074. Sigma-Aldrich). Immunoreactive bands were visualised by incubation with horseradish peroxidase-conjugated goat anti-rabbit immunoglobulins (1:5,000, Ref: 170-6515 Bio-Rad) or goat anti-mouse immunoglobulins (1:5,000, Ref: 170-6516 Bio-Rad). Protein bands were visualised using chemiluminescence reagents and a G:BOX Chemi XRQ chemiluminescence imager (Syngene).

To assess loading, membranes were stripped of previously used antibodies using a pH 2.2 glycine-HCl-SDS buffer and reprobed for tubulin (1:5,000, Ref: 6074 Sigma−Aldrich).

Knock-down of CREB using siRNA

Transfection of siRNAs was performed at the time of cell seeding in a 96-well plate format (2×10^4 cells/well). Lipofectamine 2000 transfection reagent (Invitrogen) was used following the manufacturer's instructions. Transfection experiments were carried out in Opti-MEM reduced serum medium (Invitrogen). siRNAs were used at a concentration of 20 nM, and experiments were carried out 48 h after transfection. The knockdown efficiency of the siRNA targeting murine CREB1 (Dharmacon, Ref: SO-2681745G) was quantified using RT-qPCR using conditions described above.

Adhesion, phagocytosis and intracellular survival assay

Intracellular survival experiments were carried out as previously described with minor modifications (Cano *et al*, 2015). Briefly, macrophages were seeded in 12-well tissue culture and allowed to adhere overnight at 37°C in a humidified 5% CO_2 incubator. Cells were infected at a multiplicity of infection of 70:1 in a final volume of 500 µl of antibiotic-free complete medium. To synchronise infection, plates were centrifuged at 200 *g* for 5 min. Plates were incubated at 37°C in a humidified 5% CO_2 atmosphere. After 1 h of contact, cells were washed twice with PBS and incubated for additional 30 min with 500 µl of complete medium containing gentamicin (100 µg/ml) to eliminate extracellular bacteria. For time course experiments, after 90 min, cells were washed with PBS and incubated with 500 µl of complete medium containing gentamicin (5 µg/ml). To determine intracellular bacterial load, cells were washed twice with prewarmed PBS and lysed with 300 µl of 0.05% saponin (Sigma-Aldrich) in PBS for 5 min at 37°C. Adhesion was determined at 60 min post infection, phagocytosis at 90 min post infections and survival at 330 min post infection. Serial dilutions were plated on LB to quantify the number of intracellular bacteria. All experiments were carried out in duplicate on at least three independent occasions.

©2022 The Authors

Confocal microscopy

iBMDMs were seeded in 24-well plates on 13-mm glass coverslips (VWR). Infections were performed at a multiplicity of infection of 100 bacteria per iBMDM in a final volume of 500 µl of complete medium. To synchronise infection, cells were centrifuged (200 *g* for 5 min). After 60 min contact, cells were washed with sterile PBS and incubated in 500 µl of complete medium containing gentamicin (100 µg/ml). Lysosomes were stained using cresyl violet (Ostrowski *et al*, 2016). Fifteen min before the end of the experiment, cresyl violet (Sigma-Aldrich) was added to the coverslips to achieve 5 µM of final concentration. Coverslips were washed in PBS and fixed with 4% paraformaldehyde (PFA; Sigma-Aldrich) for 20 min at room temperature. Coverslips were mounted with ProLong Gold antifade mountant (Invitrogen). Coverslips were visualised on a Leica SP5 Confocal microscope with the appropriate filter sets. Experiments were carried out in duplicate on three independent occasions.

Assessment of NF-κB and Irf3 activation

To quantify the activation of the NF-κB signalling pathway, we probed Raw-Blue cells (InvivoGen) derived from Raw 264.7 macrophages containing a chromosomal integration of a secreted embryonic alkaline phosphatase (SEAP) reported inducible by NF-κB and AP-1. Cells were maintained in DMEM supplemented with 10% heat-inactivated FBS, 50 U/ml of penicillin, 50 µg/ml of streptomycin. Two hundred micrograms per millilitre of Zeocin were added in alternate passages to maintain the reporter plasmid. Cells were seeded at a density of ~ 50,400 cells per well in a 96-well plate with complete medium without antibiotics, incubated overnight at 37°C in a humidified 5% CO_2 incubator. Cells were then infected at a multiplicity of infection of 100:1 in a final volume of 190 µl of antibiotic-free complete medium. To synchronise infection, plates were centrifuged at 200 *g* for 5 min. Plates were incubated at 37°C in a humidified 5% CO_2 atmosphere. After 1 h of contact, cells were washed twice with PBS and incubated for 5 h with 180 µl of complete medium containing gentamicin (100 µg/ml) to eliminate extracellular bacteria. Supernatants were then transferred to a new 96-well plate, and QUANTI-Blue (InvivoGen) was added in a 1:9 v/v ratio for 40 min. Absorbance was measured at 620 nm (POLARstar Omega).

To quantify the activation of Irf3 signalling pathway, Raw-Lucia ISG cells were probed. These cells are derived from Raw 264.7 macrophages after stable integration of an interferon regulatory factor (irf)-inducible Lucia luciferase reporter construct. Cells were cultured in DMEM supplemented with 10% heat-inactivated FBS, 50 U/ml of penicillin, 50 µg/ml of streptomycin. Two hundred micrograms per millilitre of Zeocin were added in alternate passages to maintain the reporter plasmid. Cells were seeded and infected as described for Raw-Blue cells. At the end of the experiment, 20 µl of media were transferred to flat, white bottom luminometer plates (LUMITRAC, Greiner), 50 µl of QUANTI-Luc (Invivogen) were added to the wells and luminescence was read immediately (Promega GloMax).

Statistics

No statistical method was used to calculate the sample size. Statistical analyses were performed with Prism (version 9.1.2) software (GraphPad Software) using one-way analysis of variance (ANOVA) with Bonferroni correction for multiple comparisons, or unpaired two-tailed Student's *t* test. Error bars indicate standard errors of the means (SEM). Statistical significance is indicated in figures as follows: ns, not significant ($P > 0.05$); *$P < 0.05$; **$P < 0.01$; ***$P < 0.001$ and ****$P < 0.0001$.

The paper explained

Problem

The emergence of multidrug-resistant *Klebsiella pneumoniae* is an important public health challenge worldwide. The World Health Organisation includes *K. pneumoniae* in the 'critical' group of pathogens for which new therapeutics are urgently needed. Although there is a wealth of knowledge on how *K. pneumoniae* develops resistance to different antibiotics, we still lack a complete understanding of what makes *K. pneumoniae* a successful pathogen. Of particular interest is to uncover whether *K. pneumoniae* deploys any strategy to manipulate macrophage function. These cells play a central role in host defence against infections and, therefore, pathogens such as *Klebsiella* should overcome them to survive in our tissues.

Results

Here, by probing the mouse pneumonia pre-clinical model, we demonstrate that alveolar macrophages restrict *Klebsiella* infection, whereas interstitial macrophages are needed for survival of the pathogen *in vivo*. We show that *Klebsiella* rewires macrophages to a new polarisation state we termed M(Kp). This state is controlled by the transcriptional factor STAT6, and *Klebsiella* hijacks type I IFN and IL10 following the activation of innate receptors to activate STAT6. These results illustrate how the co-evolution of *K. pneumoniae* with the immune system has resulted in the pathogen exploiting immune effectors and receptors sensing infections to thwart innate immune defences. Absence of STAT6 results in clearance of *K. pneumoniae*, illustrating the importance of M(Kp) in *K. pneumoniae* infection biology. Importantly, *K. pneumoniae* also rewires human macrophages towards M(Kp) in a STAT6-dependent manner.

Impact

Our findings explain why alcoholism or trauma favours *K. pneumoniae* infections as these factors will facilitate the rewire of macrophages by the pathogen. The fact that absence of STAT6 results in increased clearance of *K. pneumoniae* strongly supports that STAT6 is a target to boost human defence mechanisms against *K. pneumoniae*. Host-directed therapeutics aiming to interfere with host factors required by pathogens to counter the immune system are emerging as untapped opportunities to combat infections. There is research to develop STAT6 inhibitors, we propose that these drugs shall show a beneficial effect to treat *K. pneumoniae* infections alone or as a synergistic add-on to antibiotic treatment.

Data availability

All data needed to evaluate the conclusions in the study are present in the manuscript, the supplemental materials or uploaded in a public repository (EBI BioStudies, accession number S-BSST892). scRNA-seq data has been deposited to the NCBI Gene Expression Omnibus repository with the accession number GSE184290. The datasets produced in this study are available in the following databases:

scRNA-seq data: NCBI Gene Expression Omnibus, GSE184290 (https://www.ncbi.nlm.nih.gov/geo/query/acc.cgi?acc=GSE184290).

©2022 The Authors

Additional supportive figures: EBI BioStudies; S-BSST892 (https://www.ebi.ac.uk/biostudies/studies/S-BSST892query=S-BSST892%20).

Expanded View for this article is available online.

Acknowledgements
The authors thank the members of the J.A.B. laboratory for their thoughtful discussions and support with this project. This work was supported by Biotechnology and Biological Sciences Research Council (BBSRC, BB/P006078/1, BB/P020194/1), and Medical Research Council (MRC, MR/V032496/1) funds to J.A.B.

Author contributions
Amy Dumigan: Conceptualization; investigation; writing—original draft; writing—review and editing. **Oisin Cappa:** Investigation. **Brenda Morris:** Investigation. **Joana Sa-Pessoa:** Resources; investigation. **Ricardo Calderon-Gonzalez:** Investigation. **Grant Mills:** Investigation. **Rebecca Lancaster:** Investigation. **David A Simpson:** Investigation; writing—original draft; writing—review and editing. **Adrien Kissenpfennig:** Conceptualization; supervision; funding acquisition; writing—original draft; writing—review and editing. **Jose A Bengoechea:** Conceptualization; supervision; funding acquisition; writing—original draft; writing—review and editing.

Disclosure and competing interests statement
The authors declare that they have no conflict of interest.

References

Aegerter H, Kulikauskaite J, Crotta S, Patel H, Kelly G, Hessel EM, Mack M, Beinke S, Wack A (2020) Influenza-induced monocyte-derived alveolar macrophages confer prolonged antibacterial protection. *Nat Immunol* 21: 145–157

Ahn D, Penaloza H, Wang Z, Wickersham M, Parker D, Patel P, Koller A, Chen EI, Bueno SM, Uhlemann AC *et al* (2016) Acquired resistance to innate immune clearance promotes *Klebsiella pneumoniae* ST258 pulmonary infection. *JCI insight* 1: e89704

Ahn D, Bhushan G, McConville TH, Annavajhala MK, Soni RK, Wong Fok Lung T, Hofstaedter CE, Shah SS, Chong AM, Castano VG *et al* (2021) An acquired acyltransferase promotes *Klebsiella pneumoniae* ST258 respiratory infection. *Cell Rep* 35: 109196

Arafa EI, Shenoy AT, Barker KA, Etesami NS, Martin IM, Lyon De Ana C, Na E, Odom CV, Goltry WN, Korkmaz FT *et al* (2022) Recruitment and training of alveolar macrophages after pneumococcal pneumonia. *JCI Insight* 7: e150239

Aran D, Looney AP, Liu L, Wu E, Fong V, Hsu A, Chak S, Naikawadi RP, Wolters PJ, Abate AR *et al* (2019) Reference-based analysis of lung single-cell sequencing reveals a transitional profibrotic macrophage. *Nat Immunol* 20: 163–172

Arpaia N, Godec J, Lau L, Sivick KE, McLaughlin LM, Jones MB, Dracheva T, Peterson SN, Monack DM, Barton GM (2011) TLR signaling is required for *Salmonella typhimurium* virulence. *Cell* 144: 675–688

Bartholomew TL, Kidd TJ, Sa Pessoa J, Conde Alvarez R, Bengoechea JA (2019) 2-hydroxylation of *Acinetobacter baumannii* lipid A contributes to virulence. *Infect Immun* 87: e00066-19

Bengoechea JA, Sa Pessoa J (2019) *Klebsiella pneumoniae* infection biology: living to counteract host defences. *FEMS Microbiol Rev* 43: 123–144

Benoist C (2016) Open-source ImmGen: mononuclear phagocytes. *Nat Immunol* 17: 741–742

Biswas SK, Mantovani A (2012) Orchestration of metabolism by macrophages. *Cell Metab* 15: 432–437

Broug-Holub E, Toews GB, van Iwaarden JF, Strieter RM, Kunkel SL, Paine R 3rd, Standiford TJ (1997) Alveolar macrophages are required for protective pulmonary defenses in murine Klebsiella pneumonia: elimination of alveolar macrophages increases neutrophil recruitment but decreases bacterial clearance and survival. *Infect Immun* 65: 1139–1146

Cano V, March C, Insua JL, Aguilo N, Llobet E, Moranta D, Regueiro V, Brennan GP, Millan-Lou MI, Martin C *et al* (2015) *Klebsiella pneumoniae* survives within macrophages by avoiding delivery to lysosomes. *Cell Microbiol* 17: 1537–1560

Cao J, Spielmann M, Qiu X, Huang X, Ibrahim DM, Hill AJ, Zhang F, Mundlos S, Christiansen L, Steemers FJ *et al* (2019) The single-cell transcriptional landscape of mammalian organogenesis. *Nature* 566: 496–502

Chakarov S, Lim HY, Tan L, Lim SY, See P, Lum J, Zhang XM, Foo S, Nakamizo S, Duan K *et al* (2019) Two distinct interstitial macrophage populations coexist across tissues in specific subtissular niches. *Science* 363: eaau0964

Cortes G, Borrell N, de Astorza B, Gomez C, Sauleda J, Alberti S (2002) Molecular analysis of the contribution of the capsular polysaccharide and the lipopolysaccharide O side chain to the virulence of *Klebsiella pneumoniae* in a murine model of pneumonia. *Infect Immun* 70: 2583–2590

Deleo FR, Chen L, Porcella SF, Martens CA, Kobayashi SD, Porter AR, Chavda KD, Jacobs MR, Mathema B, Olsen RJ *et al* (2014) Molecular dissection of the evolution of carbapenem-resistant multilocus sequence type 258 *Klebsiella pneumoniae*. *Proc Natl Acad Sci U S A* 111: 4988–4993

Dolgachev VA, Yu B, Reinke JM, Raghavendran K, Hemmila MR (2012) Host susceptibility to gram-negative pneumonia after lung contusion. *J Trauma Acute Care Surg* 72: 614–622; discussion 622–613

Ducas-Mowchun K, De Silva PM, Crisostomo L, Fernando DM, Chao TC, Pelka P, Schweizer HP, Kumar A (2019) Next generation of Tn7-based single-copy insertion elements for use in multi- and Pan-drug-resistant strains of *Acinetobacter baumannii*. *Appl Environ Microbiol* 85: e00066-19

Dumigan A, Fitzgerald M, Santos JSG, Hamid U, O'Kane CM, McAuley DF, Bengoechea JA (2019) A porcine *ex vivo* lung perfusion model to investigate bacterial pathogenesis. *mBio* 10: e02802-19

Evrard B, Balestrino D, Dosgilbert A, Bouya-Gachancard JL, Charbonnel N, Forestier C, Tridon A (2010) Roles of capsule and lipopolysaccharide O antigen in interactions of human monocyte-derived dendritic cells and *Klebsiella pneumoniae*. *Infect Immun* 78: 210–219

Fairbairn L, Kapetanovic R, Sester DP, Hume DA (2011) The mononuclear phagocyte system of the pig as a model for understanding human innate immunity and disease. *J Leukoc Biol* 89: 855–871

Farlik M, Reutterer B, Schindler C, Greten F, Vogl C, Müller M, Decker T (2010) Nonconventional initiation complex assembly by STAT and NF-κB transcription factors regulates nitric oxide synthase expression. *Immunity* 33: 25–34

Finak G, McDavid A, Yajima M, Deng J, Gersuk V, Shalek AK, Slichter CK, Miller HW, McElrath MJ, Prlic M *et al* (2015) MAST: a flexible statistical framework for assessing transcriptional changes and characterizing heterogeneity in single-cell RNA sequencing data. *Genome Biol* 16: 278

Frank CG, Reguerio V, Rother M, Moranta D, Maeurer AP, Garmendia J, Meyer TF, Bengoechea JA (2013) *Klebsiella pneumoniae* targets an EGF receptor-dependent pathway to subvert inflammation. *Cell Microbiol* 15: 1212–1233

Frantz R, Teubner L, Schultze T, La Pietra L, Muller C, Gwozdzinski K, Pillich H, Hain T, Weber-Gerlach M, Panagiotidis GD *et al* (2019) The secRNome of listeria monocytogenes harbors small noncoding RNAs that are potent inducers of Beta interferon. *mBio* 10: e01223-19

 ©2022 The Authors

Gibbings SL, Thomas SM, Atif SM, McCubbrey AL, Desch AN, Danhorn T, Leach SM, Bratton DL, Henson PM, Janssen WJ *et al* (2017) Three unique interstitial macrophages in the murine lung at steady state. *Am J Respir Cell Mol Biol* 57: 66 – 76

Giske CG, Monnet DL, Cars O, Carmeli Y, ReAct-Action on Antibiotic Resistance (2008) Clinical and economic impact of common multidrug-resistant gram-negative bacilli. *Antimicrob Agents Chemother* 52: 813 – 821

Gleissner CA, Shaked I, Little KM, Ley K (2010) CXC chemokine ligand 4 induces a unique transcriptome in monocyte-derived macrophages. *J Immunol* 184: 4810 – 4818

Goenka S, Kaplan MH (2011) Transcriptional regulation by STAT6. *Immunol Res* 50: 87 – 96

Greenberger MJ, Strieter RM, Kunkel SL, Danforth JM, Goodman RE, Standiford TJ (1995) Neutralization of IL-10 increases survival in a murine model of Klebsiella pneumonia. *J Immunology* 155: 722 – 729

Heng TS, Painter MW, Immunological Genome Project Consortium (2008) The immunological genome project: networks of gene expression in immune cells. *Nat Immunol* 9: 1091 – 1094

Holt KE, Wertheim H, Zadoks RN, Baker S, Whitehouse CA, Dance D, Jenney A, Connor TR, Hsu LY, Severin J *et al* (2015) Genomic analysis of diversity, population structure, virulence, and antimicrobial resistance in *Klebsiella pneumoniae*, an urgent threat to public health. *Proc Natl Acad Sci U S A* 112: E3574 – E3581

Huang L, Nazarova EV, Tan S, Liu Y, Russell DG (2018) Growth of mycobacterium tuberculosis *in vivo* segregates with host macrophage metabolism and ontogeny. *J Exp Med* 215: 1135 – 1152

Ivin M, Dumigan A, de Vasconcelos FN, Ebner F, Borroni M, Kavirayani A, Przybyszewska KN, Ingram RJ, Lienenklaus S, Kalinke U *et al* (2017) Natural killer cell-intrinsic type I IFN signaling controls *Klebsiella pneumoniae* growth during lung infection. *PLoS Pathog* 13: e1006696

Jha AK, Huang SC, Sergushichev A, Lampropoulou V, Ivanova Y, Loginicheva E, Chmielewski K, Stewart KM, Ashall J, Everts B *et al* (2015) Network integration of parallel metabolic and transcriptional data reveals metabolic modules that regulate macrophage polarization. *Immunity* 42: 419 – 430

Lam MMC, Wyres KL, Duchene S, Wick RR, Judd LM, Gan YH, Hoh CH, Archuleta S, Molton JS, Kalimuddin S *et al* (2018) Population genomics of hypervirulent *Klebsiella pneumoniae* clonal-group 23 reveals early emergence and rapid global dissemination. *Nat Commun* 9: 2703

Lery LM, Frangeul L, Tomas A, Passet V, Almeida AS, Bialek-Davenet S, Barbe V, Bengoechea JA, Sansonetti P, Brisse S *et al* (2014) Comparative analysis of *Klebsiella pneumoniae* genomes identifies a phospholipase D family protein as a novel virulence factor. *BMC Biol* 12: 41

Liao X, Sharma N, Kapadia F, Zhou G, Lu Y, Hong H, Paruchuri K, Mahabeleshwar GH, Dalmas E, Venteclef N (2011) Krüppel-like factor 4 regulates macrophage polarization. *J Clin Invest* 121: 2736 – 2749

Liu G, Zhang H, Zhao C, Zhang H (2020) Evolutionary history of the toll-like receptor gene family across vertebrates. *Genome Biol Evol* 12: 3615 – 3634

Llobet E, Tomas JM, Bengoechea JA (2008) Capsule polysaccharide is a bacterial decoy for antimicrobial peptides. *Microbiology* 154: 3877 – 3886

Malyshev I, Lyamina S (2014) Imbalance of M1/M2 alveolar macrophages phenotype in bronchial asthma (LB506). *FASEB J* 28: LB506

March C, Cano V, Moranta D, Llobet E, Perez-Gutierrez C, Tomas JM, Suarez T, Garmendia J, Bengoechea JA (2013) Role of bacterial surface structures on the interaction of *Klebsiella pneumoniae* with phagocytes. *PloS one* 8: e56847

Martinez FO, Gordon S, Locati M, Mantovani A (2006) Transcriptional profiling of the human monocyte-to-macrophage differentiation and polarization: new molecules and patterns of gene expression. *J Immunol* 177: 7303 – 7311

Mayr B, Montminy M (2001) Transcriptional regulation by the phosphorylation-dependent factor CREB. *Nat Rev Mol Cell Biol* 2: 599 – 609

Misharin AV, Morales-Nebreda L, Reyfman PA, Cuda CM, Walter JM, McQuattie-Pimentel AC, Chen CI, Anekalla KR, Joshi N, Williams KJN *et al* (2017) Monocyte-derived alveolar macrophages drive lung fibrosis and persist in the lung over the life span. *J Exp Med* 214: 2387 – 2404

Murray PJ, Allen JE, Biswas SK, Fisher EA, Gilroy DW, Goerdt S, Gordon S, Hamilton JA, Ivashkiv LB, Lawrence T *et al* (2014) Macrophage activation and polarization: nomenclature and experimental guidelines. *Immunity* 41: 14 – 20

Nagashima S, Yokota M, Nakai E, Kuromitsu S, Ohga K, Takeuchi M, Tsukamoto S, Ohta M (2007) Synthesis and evaluation of 2-{[2-(4-hydroxyphenyl)-ethyl]amino}pyrimidine-5-carboxamide derivatives as novel STAT6 inhibitors. *Bioorg Med Chem* 15: 1044 – 1055

Nassif X, Fournier JM, Arondel J, Sansonetti PJ (1989) Mucoid phenotype of *Klebsiella pneumoniae* is a plasmid-encoded virulence factor. *Infect Immun* 57: 546 – 552

Ohama H, Asai A, Ito I, Suzuki S, Kobayashi M, Higuchi K, Suzuki F (2015) M2b macrophage elimination and improved resistance of mice with chronic alcohol consumption to opportunistic infections. *Am J Pathol* 185: 420 – 431

O'Neill LA, Bowie AG (2007) The family of five: TIR-domain-containing adaptors in toll-like receptor signalling. *Nature Rev Immunol* 7: 353 – 364

Ostrowski PP, Fairn GD, Grinstein S, Johnson DE (2016) Cresyl violet: a superior fluorescent lysosomal marker. *Traffic* 17: 1313 – 1321

Pagliuso A, Tham TN, Allemand E, Robertin S, Dupuy B, Bertrand Q, Becavin C, Koutero M, Najburg V, Nahori MA *et al* (2019) An RNA-binding protein secreted by a bacterial pathogen modulates RIG-I signaling. *Cell Host Microbe* 26: 823 – 835.e11

Pålsson-McDermott EM, O'Neill LA (2020) Targeting immunometabolism as an anti-inflammatory strategy. *Cell Res* 30: 300 – 314

Pan YJ, Lin TL, Hsu CR, Wang JT (2011) Use of a Dictyostelium model for isolation of genetic loci associated with phagocytosis and virulence in *Klebsiella pneumoniae*. *Infect Immun* 79: 997 – 1006

Pauleau A-L, Rutschman R, Lang R, Pernis A, Watowich SS, Murray PJ (2004) Enhancer-mediated control of macrophage-specific arginase I expression. *J Immunol* 172: 7565 – 7573

Penalva G, Hogberg LD, Weist K, Vlahovic-Palcevski V, Heuer O, Monnet DL, ESAC-Net Study Group, EARS-Net Study Group (2019) Decreasing and stabilising trends of antimicrobial consumption and resistance in *Escherichia coli* and *Klebsiella pneumoniae* in segmented regression analysis, European Union/European economic area, 2001 to 2018. *Euro Surveill* 24: 1900656

Piazzon MC, Lutfalla G, Forlenza M (2016) IL10, a tale of an evolutionarily conserved cytokine across vertebrates. *Crit Rev Immunol* 36: 99 – 129

Pisu D, Huang L, Narang V, Theriault M, Le-Bury G, Lee B, Lakudzala AE, Mzinza DT, Mhango DV, Mitini-Nkhoma SC *et al* (2021) Single cell analysis ofM. Tuberculosisphenotype and macrophage lineages in the infected lung. *J Exp Med* 218: e20210615

Puthenveetil A, Dubey S (2020) Metabolic reprograming of tumor-associated macrophages. *Ann Transl Med* 8: 1030

Regueiro V, Campos MA, Pons J, Alberti S, Bengoechea JA (2006) The uptake of a *Klebsiella pneumoniae* capsule polysaccharide mutant triggers an inflammatory response by human airway epithelial cells. *Microbiology* 152: 555 – 566

Regueiro V, Moranta D, Campos MA, Margareto J, Garmendia J, Bengoechea JA (2009) *Klebsiella pneumoniae* increases the levels of toll-like receptors 2 and 4 in human airway epithelial cells. *Infect Immun* 77: 714 – 724

van Rooijen N, Hendrikx E (2010) Liposomes for specific depletion of macrophages from organs and tissues. *Methods Mol Biol* 605: 189 – 203

Rosenberg G, Yehezkel D, Hoffman D, Mattioli CC, Fremder M, Ben-Arosh H, Vainman L, Nissani N, Hen-Avivi S, Brenner S *et al* (2021) Host succinate is an activation signal for salmonella virulence during intracellular infection. *Science* 371: 400 – 405

Sa-Pessoa J, Przybyszewska K, Vasconcelos FN, Dumigan A, Frank CG, Hobley L, Bengoechea JA (2020) *Klebsiella pneumoniae* reduces SUMOylation to limit host defense responses. *mBio* 11: e01733-20

Saraiva M, O'Garra A (2010) The regulation of IL-10 production by immune cells. *Nature Rev Immunol* 10: 170 – 181

Schyns J, Bai Q, Ruscitti C, Radermecker C, De Schepper S, Chakarov S, Farnir F, Pirottin D, Ginhoux F, Boeckxstaens G *et al* (2019) Non-classical tissue monocytes and two functionally distinct populations of interstitial macrophages populate the mouse lung. *Nat Commun* 10: 3964

Standiford LR, Standiford TJ, Newstead MJ, Zeng X, Ballinger MN, Kovach MA, Reka AK, Bhan U (2012) TLR4-dependent GM-CSF protects against lung injury in gram-negative bacterial pneumonia. *Am J Physiol Lung Cell Mol Physiol* 302: L447 – L454

Stuart T, Butler A, Hoffman P, Hafemeister C, Papalexi E, Mauck WM 3rd, Hao Y, Stoeckius M, Smibert P, Satija R (2019) Comprehensive integration of single-cell data. *Cell* 177: 1888 – 1902.e21

Szanto A, Balint BL, Nagy ZS, Barta E, Dezso B, Pap A, Szeles L, Poliska S, Oros M, Evans RM *et al* (2010) STAT6 transcription factor is a facilitator of the nuclear receptor PPARgamma-regulated gene expression in macrophages and dendritic cells. *Immunity* 33: 699 – 712

Tsuchimoto Y, Asai A, Tsuda Y, Ito I, Nishiguchi T, Garcia MC, Suzuki S, Kobayashi M, Higuchi K, Suzuki F (2015) M2b monocytes provoke bacterial pneumonia and gut bacteria-associated sepsis in alcoholics. *J Immunol* 195: 5169 – 5177

Wang Y, Levy DE (2012) Comparative evolutionary genomics of the STAT family of transcription factors. *JAKSTAT* 1: 23 – 33

Wen AY, Sakamoto KM, Miller LS (2010) The role of the transcription factor CREB in immune function. *J Immunol* 185: 6413 – 6419

Wingender E, Dietze P, Karas H, Knüppel R (1996) TRANSFAC: a database on transcription factors and their DNA binding sites. *Nucleic Acids Res* 24: 238 – 241

Xiong H, Carter RA, Leiner IM, Tang YW, Chen L, Kreiswirth BN, Pamer EG (2015) Distinct contributions of neutrophils and $CCR2^+$ monocytes to pulmonary clearance of different *Klebsiella pneumoniae* strains. *Infect Immun* 83: 3418 – 3427

Xiong H, Keith JW, Samilo DW, Carter RA, Leiner IM, Pamer EG (2016) Innate lymphocyte/Ly6C(hi) monocyte crosstalk promotes *Klebsiella pneumoniae* clearance. *Cell* 165: 679 – 689

Yang FL, Yang YL, Liao PC, Chou JC, Tsai KC, Yang AS, Sheu F, Lin TL, Hsieh PF, Wang JT *et al* (2011) Structure and immunological characterization of the capsular polysaccharide of a pyrogenic liver abscess caused by *Klebsiella pneumoniae*: activation of macrophages through toll-like receptor 4. *J Biol Chem* 286: 21041 – 21051

License: This is an open access article under the terms of the Creative Commons Attribution License, which permits use, distribution and reproduction in any medium, provided the original work is properly cited.

Expanded View Figures

Figure EV1. Analysis of *Klebsiella pneumoniae*-induced transcriptome in IMs.

A Diagram of the experimental approach to generate the different IMs and AMs samples for single-cell RNA sequencing (scRNAseq). C57BL/6 mice ($n = 17$ per group) were infected intranasally with mCherry tagged Kp52145, after 24 h, lungs were excised, and processed for cell sorting. From pooled samples AM and IM populations were sorted from PBS controls and infected mice. In the latter group, cells were sorted to separate bystander cells and cells with associated bacteria. The viability of each of the samples was determined to be higher than 95% before carrying out 10× genomics single-cell RNA sequencing.

B Marker gene detection and differential expression testing was performed in Seurat using the MAST package. Higher resolution clustering using uniform manifold approximation and projection (UMAP) dimensionality reduction analysis showing selected genes, *cx3cr1*, IM marker and *siglecF*, AM marker.

C UMAP of clustering within cells from PBS mock-infected mice (control), bystander and Kp52145-associated IMs and AM populations.

D Network enrichment mapping generated from significantly upregulated genes of IMs with associated bacteria. Analysis was performed using the g:SCS method for multiple testing correction (gProflier), the Reactome database as a data source and the default settings for the other parameters in gProflier. Results were exported to Cytoscape and visualised using the AutoAnnotate plug.

E Network enrichment mapping generated from significantly upregulated genes of bystander IMs. Analysis was performed using the g:SCS method for multiple testing correction (gProflier), the Reactome database as a data source and the default settings for the other parameters in gProflier. Results were exported to Cytoscape and visualised using the AutoAnnoate application.

F Monocle analysis to determine the temporal pattern of gene expression over pseudotime in bystander and Kp52145-associated IMs from infected animals compared to PBS controls. Monocle analysis revealed 7 modules of genes showing similar pattern of expression.

G Heat map showing relative expression of the 7 modules found in IMs.

H Pathway analysis of modules 3 and 4 corresponding to Kp52145-infected IMs. Analysis was performed using the g:SCS method for multiple testing correction, the Reactome database as a data source and the default settings for the other parameters in G:profiler.

I STRING database was used to predict protein–protein interactions using the clustering algorithm MCL with default parameters using as data source the genes within modules 3 and 4.

J Pathway analysis of module 6 corresponding to bystander IMs. Analysis was performed using the g:SCS method for multiple testing correction, the Reactome database as a data source and the default settings for the other parameters in G:profiler.

©2022 The Authors EMBO Molecular Medicine 14: e16888 | 2022 27 of 26

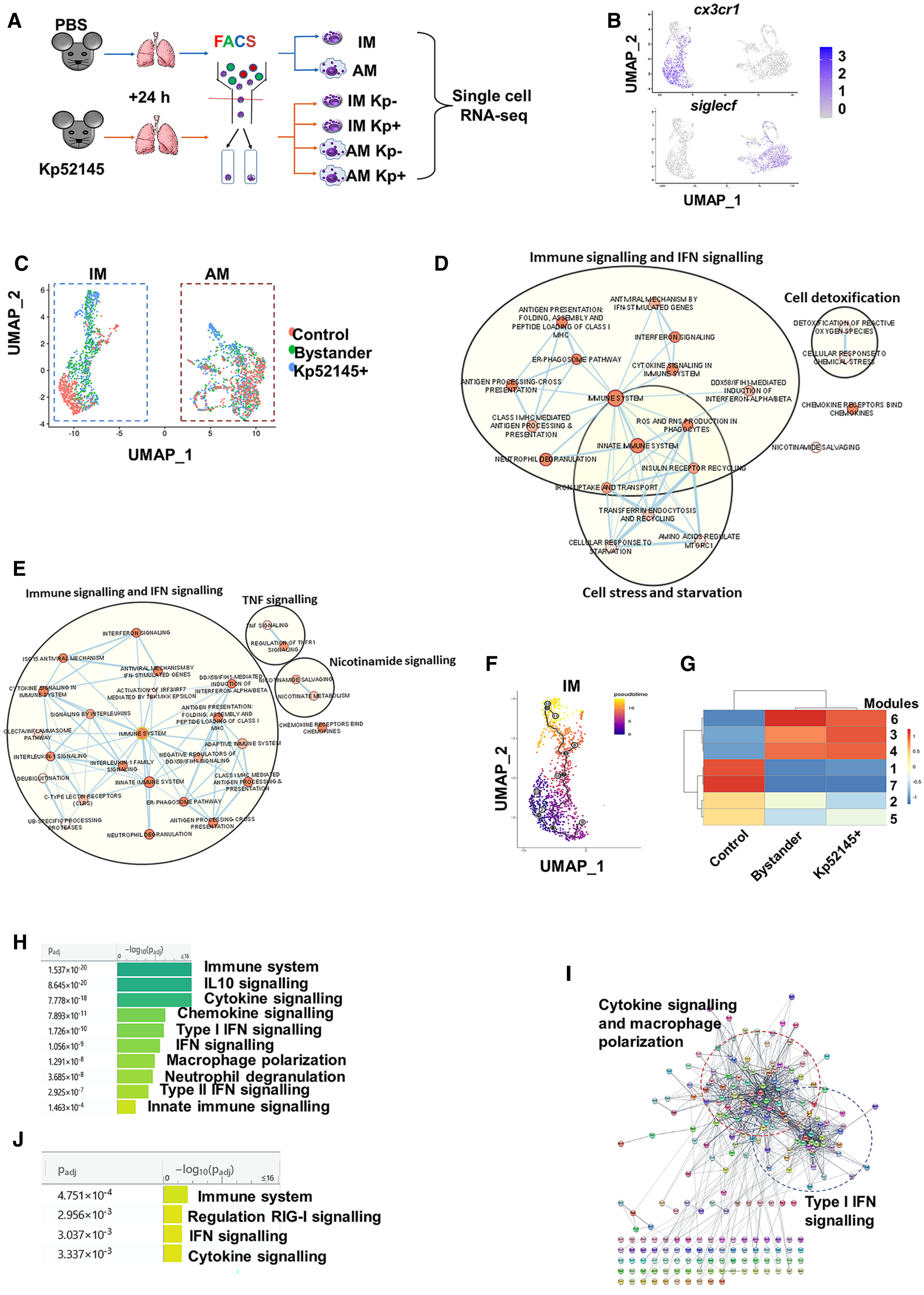
A
PBS
+24 h
Kp52145
FACS
IM
AM
IM Kp-
IM Kp+
AM Kp-
AM Kp+
Single cell RNA-seq
B
cx3cr1
siglecf
UMAP_2
UMAP_1
C
IM
AM
Control
Bystander
Kp52145+
D
Immune signalling and IFN signalling
Cell detoxification
Cell stress and starvation
E
Immune signalling and IFN signalling
TNF signalling
Nicotinamide signalling
F
IM
UMAP_2
UMAP_1
G
Modules
Control
Bystander
Kp52145+
H
Immune system
IL10 signalling
Cytokine signalling
Chemokine signalling
Type I IFN signalling
IFN signalling
Macrophage polarization
Neutrophil degranulation
Type II IFN signalling
Innate immune signalling
I
Cytokine signalling and macrophage polarization
Type I IFN signalling
J
Immune system
Regulation RIG-I signalling
IFN signalling
Cytokine signalling

Figure EV1.

Figure EV2. *Klebsiella pneumoniae* skews macrophage polarisation towards a singular state termed M(Kp).

A Heat map presents relative expression of the indicated genes between IMs from non-infected mice (control), and bystander and Kp52145-associated IMs from infected mice. Selected genes are related to M1 and M2 macrophage polarisation.

B Expression of M1 and M2 genes (shown in panel A) calculated as the average log-normalised expression. Each dot represents a cell, and the graph shows the mean and SEM per group of M1 and M2 genes. Statistical analysis were carried out using unpaired *t* test. The *P* values are indicated in the figure.

C–E Analysis by flow cytometry of the levels of M(Kp) markers expressed by cells from PBS mock infected mice (black dots), and by cells from infected mice (blue dots) with and without associated Kp52145. (C) Percentage of positive cells for Arg1. (D) Percentage of positive cells for Fizz1. (E) Percentage of positive cells for CD163.

Data information: Values in panel C-E are presented as the mean ± SEM whereby each dot represents an individual animal. ****$P \leq 0.0001$; **$P \leq 0.01$; ns, $P > 0.05$ for the indicated comparisons using one way-ANOVA with Bonferroni contrast for multiple comparisons test.

©2022 The Authors

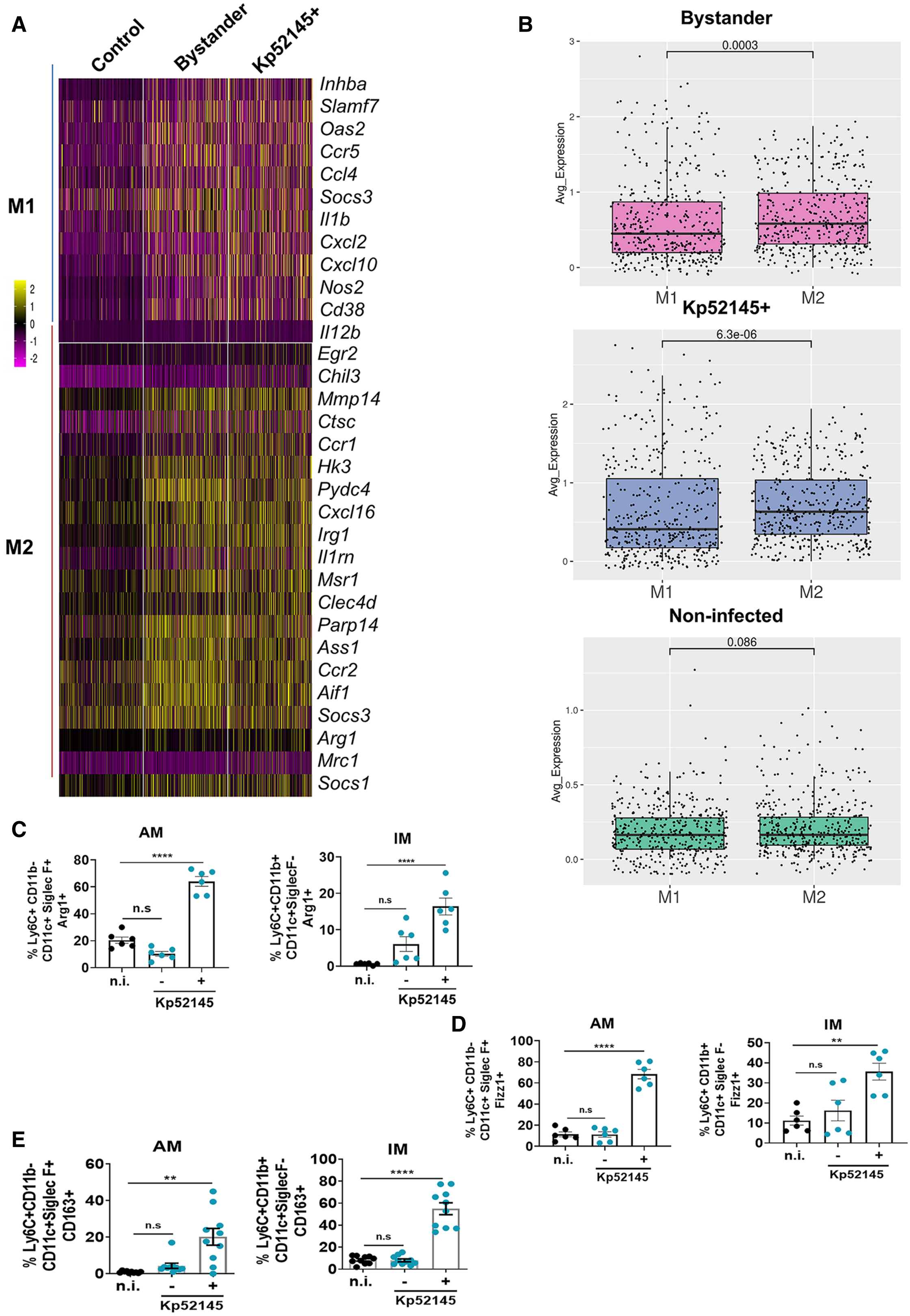

Figure EV2.

©2022 The Authors

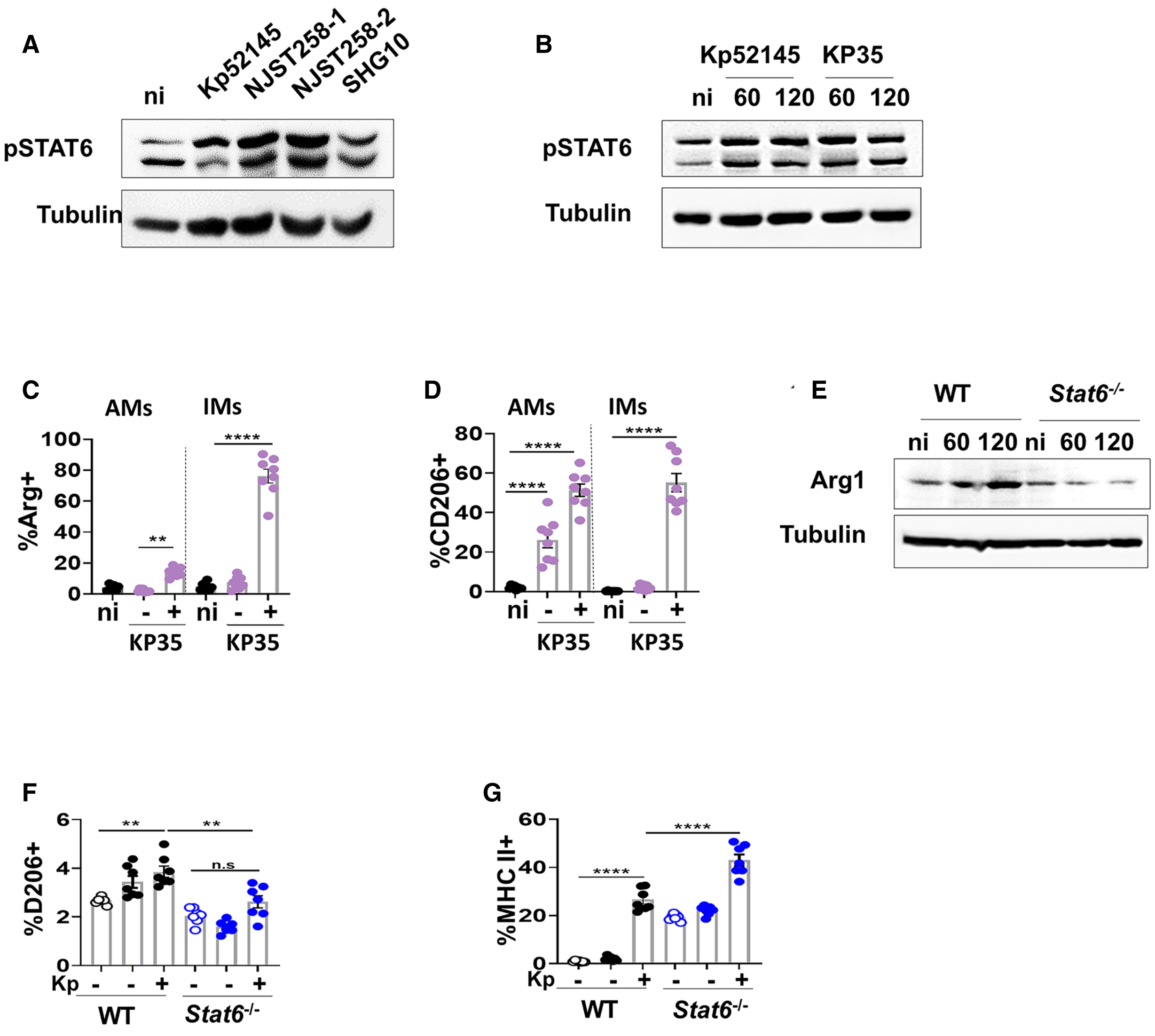

Figure EV3. ST258 *Klebsiella pneumoniae* strains induced M(Kp) polarisation.

- A Immunoblot analysis of phospho-STAT6 (pSTAT6) and tubulin levels in lysates from non-infected (ni) and infected with different *K. pneumoniae* strains, Kp52145, NJST258-1, NJST258-2 or SHG10, for 60 min.
- B Immunoblot analysis of phospho-STAT6 (pSTAT6) and tubulin levels in lysates from non-infected (ni) and infected with Kp52145 or KP35 for 60 or 120 min.
- C, D Analysis by flow cytometry of the levels of M(Kp) markers expressed by IMs and AMs from PBS-mock-infected mice (black dots), and by IMs and AMs from infected mice (purple dots) with and without associated KP35 24 h post infection. (C) Percentage of positive cells for Arg1. (D) Percentage of positive cells for CD206.
- E Immunoblot analysis of Arg1 and tubulin levels in lysates from wild-type and *stat6*$^{-/-}$ iBMDMs non-infected (ni) and infected with KP35 for 60 min or 120 min.
- F Percentage of wild-type (WT) and *stat6*$^{-/-}$ iBMDMs with and without associated KP35 positive for CD206 5 h post infection. KP35 was tagged with mCherry.
- G Percentage of wild-type (WT) and *stat6*$^{-/-}$ iBMDMs with and without associated KP35 positive for MCH-II 5 h post infection. KP35 was tagged with mCherry.

Data information: For all infections, after 1 h contact, medium replaced with medium containing gentamycin (100 μg/ml) to kill extracellular bacteria. Images are representative of three independent experiments. Error bars are presented as the mean ± SEM of three independent experiments in duplicate. Statistical analysis were carried out using one-way ANOVA with Bonferroni contrast for multiple comparisons test. ****$P \leq 0.0001$; **$P \leq 0.01$; ns, $P > 0.05$ for the indicated comparisons.

Figure EV4. Glycolysis characterises *Klebsiella pneumoniae*-induced M(Kp) polarisation.

A Dot Plot analysis of the expression levels of genes related to fatty acid oxidation (FAO) and glycolysis from the scRNAseq data set of PBS-infected IMs (control), and bystander and Kp52145-associated IMs. Dot size reflects percentage of cells in a cluster expressing each gene; dot colour intensity reflects expression level as indicated on legend.

B Extracellular acidification rate (ECAR, in mpH/min) of non-infected (ni) and Kp52145-infected iBMDMS (Kp52145) measured using Mito-stress test kit and the Seahorse XF analyser. When indicated oligomycin (2.5 μM), FCCP (2 μM), antimycin and roteanone (0.5 μM) were added to the cells.

C Oxygen consumption rates (OCR, in pMoles/min) of non-infected (ni) and Kp52145-infected iBMDMS (Kp52145) measured using Mito-stress test kit and the Seahorse XF analyser. When indicated oligomycin (2.5 μM), FCCP (2 μM), antimycin and roteanone (0.5 μM) were added to the cells.

D Basal respiration of non-infected (ni) and Kp52145-infected iBMDMs.

E Maximal respiration of non-infected (ni) and Kp52145-infected iBMDMs.

F Spare respiratory capacity of non-infected (ni) and Kp52145-infected iBMDMs.

G ATP production by non-infected (ni) and Kp52145-infected iBMDMs.

H Non mitochondrial O_2 consumption by non-infected (ni) and Kp52145-infected iBMDMs.

I Kp52145 intracellular survival in wild-type iBMDMs 5 h after addition of gentamycin (30 min of contact). Results are expressed as % of survival (CFUs at 5 h versus 30 min in *stat6*$^{-/-}$ cells normalised to the results obtained in wild-type macrophages set to 100%). Cells were treated with DMSO vehicle, or 2-deoxyglucose (2DG, 3 μM), oligomycin (3 μM), etomoxir (50 μM) 2 h before infection and maintained thought.

J Immunofluorescence confocal microscopy of the co-localisation of Kp52145 harbouring pFPV25.1Cm, and cresyl violet dye in wild-type macrophages treated with DMSO vehicle solution (control) or 2DG. The images were taken 90 min post infection. Images are representative of duplicate coverslips of three independent experiments.

K Percentage of Kp52145 harbouring pFPV25.1Cm co-localisation with cresyl violet over a time course. Wild-type iBMDMs treated with DMSO vehicle solution (control) or 2DG. were infected; coverslips were fixed and stained at the indicated times. Values are given as mean percentage of Kp52145 co-localising with the marker ± SEM. The number of infected cells counted per time in three independent experiments are indicated in the figure.

Data information: Error bars are presented as the mean ± SEM of three independent experiments in duplicate. Images are representative of three independent experiments. In panels (D–H) and (K), unpaired *t* test was used to determine statistical significance. In all the other panels, statistical analysis were carried out using one-way ANOVA with Bonferroni contrast for multiple comparisons test. ****$P \leq 0.0001$; ***$P \leq 0.001$; **$P \leq 0.01$; ns, $P > 0.05$ for the indicated comparisons.

 ©2022 The Authors

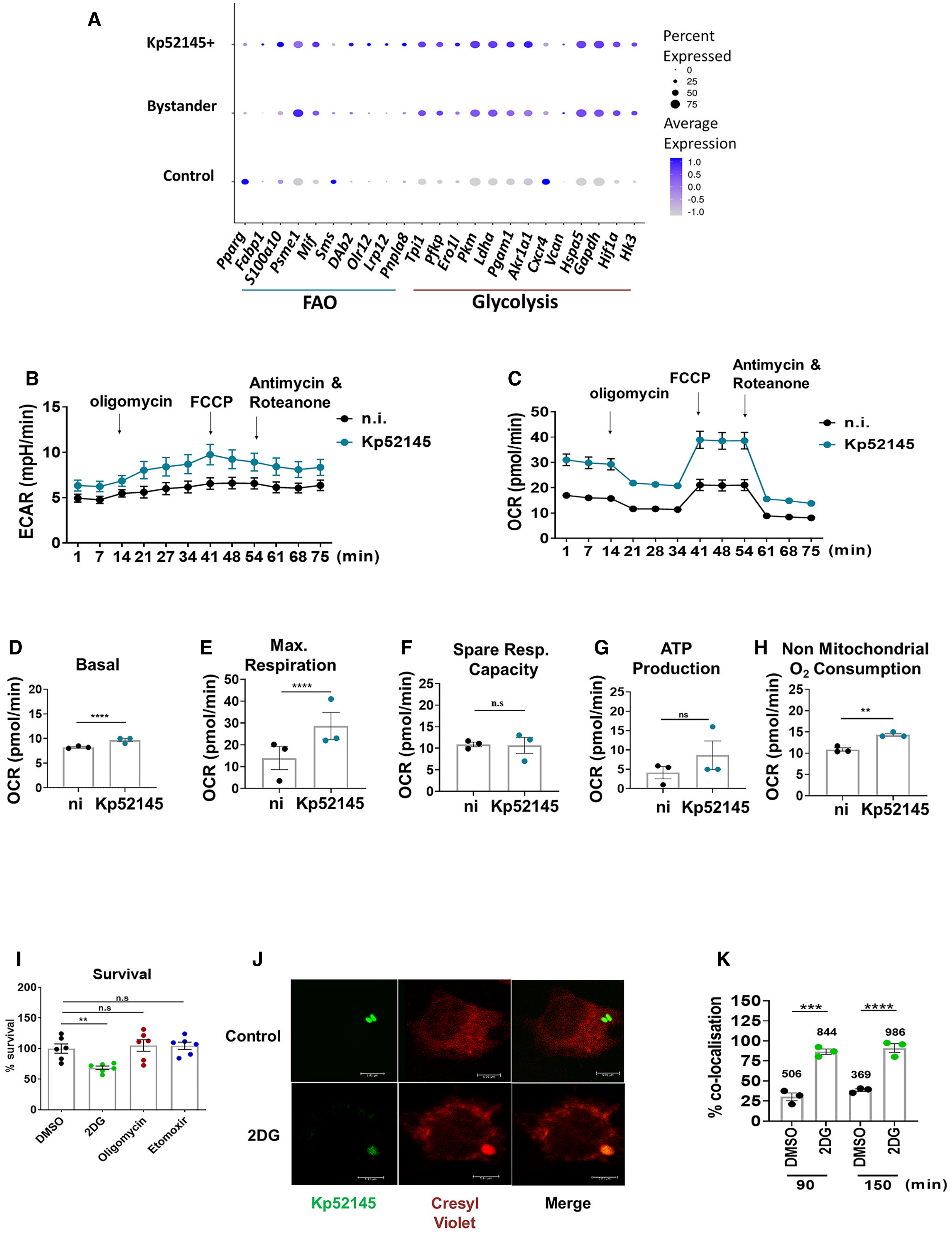
A
Kp52145+
Bystander
Control
Percent Expressed
0
25
50
75
Average Expression
1.0
0.5
0.0
-0.5
-1.0
Pparg Fabp1 S100a10 Psme1 Mif Sms DAb2 Olr12 Lrp12 Pnpla8 Tpi1 Pfkp Ero1l Pkm Ldha Pgam1 Akr1a1 Cxcr4 Vcan Hspa5 Gapdh Hif1a Hk3
FAO
Glycolysis
B
oligomycin
FCCP
Antimycin & Roteanone
n.i.
Kp52145
ECAR (mpH/min)
1 7 14 21 27 34 41 48 54 61 68 75 (min)
C
oligomycin
FCCP
Antimycin & Roteanone
n.i.
Kp52145
OCR (pmol/min)
1 7 14 21 28 34 41 48 54 61 68 75 (min)
D
Basal
E
Max. Respiration
F
Spare Resp. Capacity
G
ATP Production
H
Non Mitochondrial O2 Consumption
OCR (pmol/min)
ni Kp52145
****
n.s
ns
**
I
Survival
% survival
DMSO 2DG Oligomycin Etomoxir
J
Control
2DG
Kp52145
Cresyl Violet
Merge
K
% co-localisation
***
****
506 844 369 986
DMSO 2DG DMSO 2DG
90 150 (min)

Figure EV4.

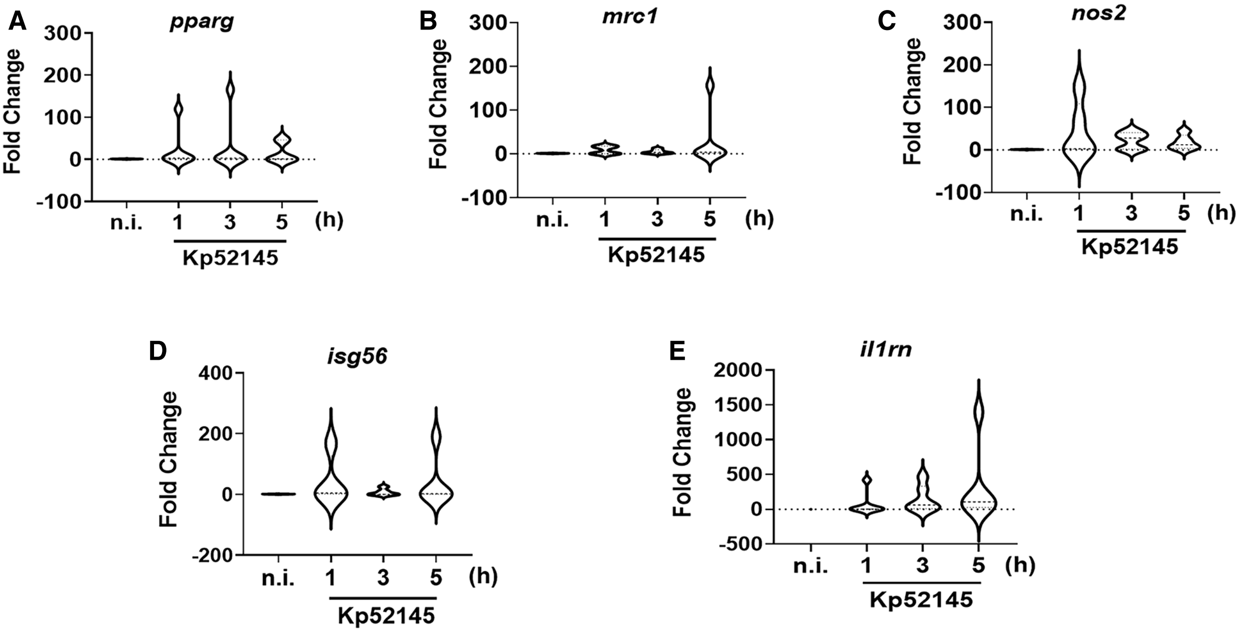

Figure EV5. *Klebsiella pneumoniae* induces M(Kp) polarisation in human macrophages.

A *pparg* mRNA levels were assessed by qPCR in hM-CSF-treated PBMCs from 6 donors non-infected (ni) or infected Kp52145 for 1, 3 or 5 h.
B *mrc1* mRNA levels were assessed by qPCR in hM-CSF-treated PBMCs from 6 donors non-infected (ni) or infected Kp52145 for 1, 3 or 5 h.
C *nos2* mRNA levels were assessed by qPCR in hM-CSF-treated PBMCs from 6 donors non-infected (ni) or infected Kp52145 for 1, 3 or 5 h.
D *isg56* mRNA levels were assessed by qPCR in hM-CSF-treated PBMCs from 6 donors non-infected (ni) or infected Kp52145 for 1, 3 or 5 h.
E *il1rn* mRNA levels were assessed by qPCR in hM-CSF-treated PBMCs from 6 donors non-infected (ni) or infected Kp52145 for 1, 3 or 5 h.

Data information: For all infections, after 1 h contact, medium replaced with medium containing gentamycin (100 μg/ml) to kill extracellular bacteria. Error bars are presented as the mean ± SEM of three independent experiments in duplicate.

 ©2022 The Authors
